# Child Maltreatment Education: Utilizing an Escape Room Activity to Engage Learners on a Sensitive Topic

**DOI:** 10.21980/J84H1C

**Published:** 2023-01-31

**Authors:** Shelley Brukman, Makenzie J Ferguson, Kimberly D Zaky, Chloe Knudsen-Robbins, Theodore W Heyming

**Affiliations:** * Children’s Hospital of Orange County, Department of Trauma Surgery, Orange, CA; ^ University of Pittsburgh, School of Medicine, Pittsburgh, PA; † Children’s Hospital of Orange County, Department of Emergency Medicine, Orange, CA

## Abstract

**Audience:**

Emergency medical service (EMS) providers and other health care professionals.

**Introduction:**

In 2019 alone, 656,000 children in the United States were victims of child abuse and neglect.^1^ The medical community has historically struggled with the identification of child maltreatment. In one study, 33% of abused children had a previous visit with a medical provider in which the abuse was found to have been missed.^2^ Many voices in the healthcare community have advocated for the implementation of routine screening, and studies have demonstrated the implementation of such screening in the emergency department (ED) increases the detection of child maltreatment.^3^–^7^ Child maltreatment screening tools are increasingly utilized in primary care and ED settings, but one has yet to be adapted or designed for universal use by emergency medical services (EMS) professionals in the prehospital care context. Because EMS providers are uniquely positioned to assess for maltreatment, they have traditionally been the only provider to interact with families in the home environment. Unfortunately, EMS rates of documentation of maltreatment is quite low. A recent study using the National Emergency Medical Services Information System database to evaluate EMS documentation of child maltreatment in patients ≤3 years of age compared to the national incidence of known maltreatment found an almost 15-fold discrepancy.^8^ There have been several attempts to elucidate the difficulties of and barriers to reporting by EMS providers. Markenson et al and Tiyyagura et al outlined several areas that potentially contribute to a lack of reporting: minimal continuing medical education (CME) on child maltreatment, knowledge of physical and historical details suspicious for abuse, knowledge of child development, limited clinical evaluation time in a fast-paced work environment, understanding of how to appropriately interact with families, and fear of being wrong.^9^,^10^ This class/escape room activity was developed to directly address several of these areas. Emergency medical service providers participate in traditional didactics (in the form of a short lecture), followed by an escape room activity in which they further explore and reinforce learning in a fun and memorable environment. This activity also promotes teamwork, an especially important skill in potentially complex and difficult situations such as those surrounding suspected child maltreatment.

**Educational Objectives:**

By the end of the escape room, the learner should be able to: 1) understand the national and local prevalence of child maltreatment; 2) understand the different types of child maltreatment and common associated presentations; 3) know the local EMS agency reporting requirements; 4) understand when to make base hospital contact with respect to concern for maltreatment; 5) collaborate effectively as a team.

**Educational Methods:**

Child maltreatment can be a sensitive and challenging topic. In this class, we presented learners with a short, 15-minute lecture (see Pre-Escape Room Lecture PowerPoint) followed by an escape room activity. The Pre-Escape Room Lecture PowerPoint includes suggestions on the type of image and/or statistics to include on each slide, which can be taken from your site’s available de-identified photos and information. The lecture included material describing national and local statistics on child maltreatment, definitions of abuse, and techniques to help identify concern for maltreatment. Learners were free to ask questions following lecture. They were then divided into their assigned crews/teams for the escape room activity. The puzzles in the escape room served to reinforce concepts and details presented in lecture. We held a debrief after the escape room activity to discuss puzzle answers and address any follow-up questions.

**Research Methods:**

Learners completed a program evaluation after the activity. These questions assessed the learners’ perception of the importance and applicability of the content presented, the escape room format, and what they felt was the most significant and helpful to their practice.

**Results:**

Learners reported enjoying the activity and felt the escape room-based approach allowed for deeper engagement with the topic since the serious nature of child maltreatment can sometimes make this difficult.

**Discussion:**

Pediatric abuse and neglect is a serious and often heavy topic to present to healthcare providers. While we took into consideration that presenting a sensitive topic such as child abuse in an escape room format may be perceived as insensitive or display a lack of insight or respect for the topic, we also understood that the way we built out the clues and puzzles would be important in how the game was perceived by the participants. By building the puzzles to be factual and not overly excessive, we allowed the learners to interact with the information and practice identifying possible cases of abuse and how and when to report suspicions in a manner that did not trivialize the seriousness of the topic or take away from the fact that they were competing in a game. We used a PowerPoint lecture to present the foundation of the content and then lightened the learning session with the use of the escape room activity. The level of competition and comradery lightened the overall mood, and the learners left the class on a high note.

**Topics:**

Child abuse recognition, escape room activity, small-group activity, prehospital, neglect, physical abuse, emotional abuse, sexual abuse, mandated reporter.

## USER GUIDE

**Table t1-jetem-8-1-sg1:** 

List of Resources:	
Abstract	1
User Guide	3
Small Groups Learning Materials	7
Appendix A: Pre-Escape Room Lecture PowerPoint	7
Appendix B: Instructions for the Escape Room	8
Appendix C: Escape Room Answers	20
Appendix D: Escape Room Debrief PowerPoint	21

[Table t1-jetem-8-1-sg1] **Learner Audience:**

EMT and Paramedic

**Time Required for Implementation:**

Lecture preparation: Adapting the Pre-Escape Room Lecture PowerPoint will depend on your access to images and case studies from your site. We suggest reaching out to your child abuse specialists, utilizing journal articles, and an internet search for such images recommended in the PowerPoint. Supply preparation: Initially our team borrowed boxes and locks from our educational department. We then purchased our own boxes, locks, UV lights, blank puzzle kit, and invisible ink pens from Amazon which shipped within the week. We utilized the emergency department laminator to laminate some of our clues. The initial build of the escape room puzzles took approximately one day of preparation, and we created two identical escape rooms to run simultaneously. The two escape rooms took between 30–45 minutes to set up on the day of the session.

The lecture prior to the escape room was 15 minutes in duration. The run time of the escape room is approximately 20 minutes. The debrief takes approximately 10 minutes.

**Recommended Number of Learners per Instructor:**

One instructor per room. The instructor should have basic knowledge of prehospital county-specific policy with regards to online medical control (consultation between EMS and a physician to guide care) access and child abuse policies. The maximum number of learners in the escape room should not be more than six. A typical fire-based EMS crew is four members with potentially two ambulance providers.

**Topics:**

Child abuse recognition, escape room activity, small-group activity, prehospital, neglect, physical abuse, emotional abuse, sexual abuse, mandated reporter.

**Objectives:**

By the end of the escape room the learner should be able to:

1. Understand the national and local prevalence of child maltreatment.
2. Understand the different types of child maltreatment and common associated presentations.
3. Know the local EMS agency reporting requirements.
4. Understand when to make base hospital contact with respect to concern for maltreatment.
5. Collaborate effectively as a team.

### Linked objectives and methods

Puzzle number 1: Kid Doe’s Suspected Child Abuse Report. This puzzle exposes the learner to the documentation submitted when reporting suspected abuse to Child Protective Services (CPS). This puzzle meets objectives 3 and 5. Puzzle number 2: Child Abuse Stats and the Hidden Answers. This puzzle helps the learner understand the scope of the problem by reviewing statistics on child abuse. This puzzle meets objectives 1 and 5 (Figures 1 and 2). Puzzle number 3: Local EMS Policy for prehospital providers. This puzzle details the local EMS policy on mandated reporting of suspected abuse. The puzzle helps the learner identify the number to call, the timeframe, and who is required to submit the report. This puzzle meets objectives 3 and 5. Puzzle number 4: Shield Protector Case Studies. This puzzle presents 6 cases and questions the learner must answer correctly to solve the wheel decoder. Puzzle number 4 meets objectives 2, 4, and 5. Puzzle number 5: Play-Doh Puzzle. This puzzle requires the key found in the previous puzzle to be used to open the last box that contains a puzzle that must be assembled. This puzzle reinforces online medical control in suspected abuse cases. Knowledge is strengthened through the debriefing session after the escape room.

### Recommended pre-reading for facilitator

The local EMS Agency policy on managing pediatric abuse cases and when online medical control is required. Our recommendation for instructors is also to be familiar with all content included in the supplemental PowerPoint presentations prior to giving the presentation and conducting the escape room activity.

### Learner responsible content (LRC)

The learners are mandated child abuse reporters and should have a basic understanding of abuse recognition and reporting.

### Small group application exercise (sGAE)

The escape room puzzles are placed on a desk in the room. The child abuse statistics are individually printed and posted on the walls around the room. We created two duplicate escape rooms to run simultaneously.

### Materials List

If budget is a factor, and purchasing locks and boxes are not an option, the box and lock items on this list can be exchanged for envelopes that the escape room facilitator can provide to the learners as they solve the clues. We also recommend laminating the paper clues the learners will interact with to improve the longevity of the game pieces.

1. Dry storage locking box. We used the Plano 131252 Dry Storage Emergency Marine Box. At: https://www.amazon.com/Plano-131252-Storage-Emergency-Marine/dp/B009YSFT7S/ref=pd_lpo_1?pd_rd_i=B08PL8248S&psc=1
2. 4-digit lock. We used Puroma 2 pack combination lock. At: https://www.amazon.com/Puroma-Combination-Padlock-Toolbox-Storage/dp/B075DFPR2W/ref=sr_1_1?crid=1PRQXNL0I54LA&dchild=1&keywords=puroma%2B2%2Bpack%2Bcombination%2Block%2B4%2Bdigit%2Bpadlock&qid=1635194958&sprefix=puroma%2B2%2Bpack%2B%2Caps%2C114&sr=8-1&th=1
3. Unfinished wooden boxes. We used Juvale boxes. At: https://www.amazon.com/Juvale-Wooden-Boxes-Hinged-Lid-Unfinished/dp/B07C2K4G4P/ref=sr_1_5?crid=1C5YZ9AE0OEEH&dchild=1&keywords=juvale+unfinished+boxes&qid=1635195100&sprefix=juvale+unfinished+boxes%2Caps%2C118&sr=8-5
4. Pack of mini screw hooks – we used these to be able to attach locks to the wooden boxes. At: https://www.amazon.com/Ceiling-Pieces-Screw-Hanging-Decorations/dp/B081T1Z8C3/ref=sr_1_3?crid=133BVKAFL9E1B&dchild=1&keywords=mini%2Bceiling%2Bscrew%2Bhooks&qid=1635195281&sprefix=mini%2Bceiling%2Bscew%2Bhooks%2Caps%2C122&sr=8-3&th=1
5. 3-digit lock. Any lock brand will work for the escape room. At: https://www.amazon.com/Eilin-Combination-School%E3%80%81Home%E3%80%81Office%E3%80%81Storage-Lockers%E3%80%81Gym-Lockers%E3%80%81Drawers%E3%80%81Cabinets%E3%80%81Toolboxes%E3%80%81Luggage/dp/B07L6XGWC4/ref=sr_1_16?crid=OIBQHZT25BGR&dchild=1&keywords=3-digit+combination+lock+pack&qid=1635195417&sprefix=3-digit+combination+lock+packs%2Caps%2C135&sr=8-16
6. Lock box with key. Any brand will work for the escape room. At: https://www.amazon.com/Vaultz-Locking-Supply-Inches-VZ03708/dp/B015MPUBEM/ref=sr_1_26?crid=1OBX0MAUWMH12&dchild=1&keywords=kids%2Block%2Bbox%2Bwith%2Bkey&qid=1635195603&sprefix=kids%2Block%2Bbox%2Caps%2C150&sr=8-26&th=1
7. Blank puzzle pieces. We used Hygloss 12-piece puzzles. At: https://www.amazon.com/dp/B00I7DUEQ2/ref=redir_mobile_desktop?_encoding=UTF8&aaxitk=eab7ecdcfd63f528fc919a1674258132&hsa_cr_id=9121159900501&pd_rd_plhdr=t&pd_rd_r=2ba60569-ac99-4dc3-90f7-82b0902fb590&pd_rd_w=SxAO7&pd_rd_wg=gY6Lx&ref_=sbx_be_s_sparkle_mcd_asin_1_img&th=1
8. Invisible ink pens with UV light. Any brand will work for the escape room. At: https://www.amazon.com/STENDA-Invisible-Blacklight-Christmas-Thanksgiving/dp/B08XW8L2NX/ref=sr_1_4?crid=2UHCL09IJ1TV6&dchild=1&keywords=invisible+ink+pens+with+uv+light+for+kids&qid=1635195889&sprefix=ivisible+ink+pens%2Caps%2C124&sr=8-4
9. Play-doh assorted colors. We used the party pack containing 12 different colors. At: https://www.amazon.com/Play-Doh-Party-Dough-assortedcolors/dp/B01J7WB866/ref=sr_1_23?crid=1XVVRP43V7BLP&dchild=1&keywords=small%2Bplaydough%2Btubs&qid=1635196035&sprefix=small%2Bplay%2Bough%2Btubs%2Caps%2C123&sr=8-23&th=1
10. Decoder wheel template. At: https://frugalfun4boys.com/code-activity-kids-make-spy-decoder/
11. Hobby knife. Any brand will work to create the cutouts for the first puzzle. At: https://www.amazon.com/Stainless-Cutting-Carving-Scrapbooking-Creation/dp/B0899VKWMB/ref=asc_df_B0899VKWMB/?tag=hyprod-20&linkCode=df0&hvadid=475811287390&hvpos=&hvnetw=g&hvrand=17695763987983061778&hvpone=&hvptwo=&hvqmt=&hvdev=c&hvdvcmdl=&hvlocint=&hvlocphy=9031600&hvtargid=pla-1027999032707&th=1
12. Letter-sized card stock colored paper, three sheets.
13. Two letter-sized envelopes.
14. Printer to print clues. Scotch tape. Laminator with pouches.

### Results and tips for successful implementation

This escape room was administered to 130 EMTs and paramedics in 12 sessions. Each learner was given an evaluation form to complete at the end of the class. Learners were asked to rate three questions using the following scale:

1 = should improve
2 = satisfactory
3 = average
4= excellent

Question 1: Information presented was important and pertinent to my practice: 95% of learners rated this as excellent (4). Less than 1% of learners rated this question as average (3) and less than 1% rated this question as satisfactory (2). Question 2: Forum provided good information and communication: 95% of learners rated this as excellent (4) and less than 1% rated this question as satisfactory (2). Question 3: Program met stated objectives: 95% of learners rated this as excellent (4) and less than 1% rated this question as satisfactory (2). Learners were able to write in comments and 48 of them felt that the information presented on recognizing child abuse was the most significant item in the class. Thirty-one participants commented “great class” and that the escape room was fun. One of the learners appreciated the team-building aspect of the escape room.

### Pearls

**Figure:**
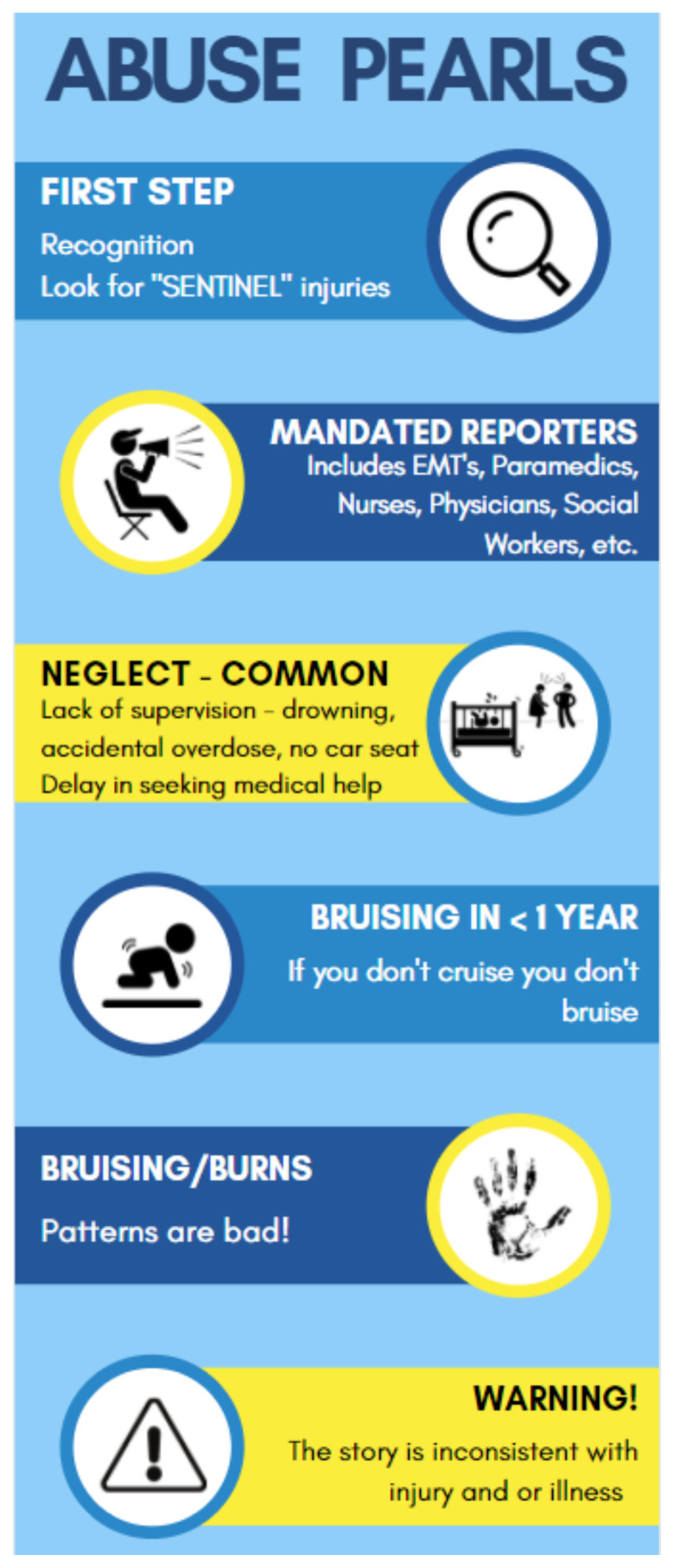


Reference tool used to summarize important information covered in the escape room activity.

## Figures and Tables

**Image 1 and 2:**
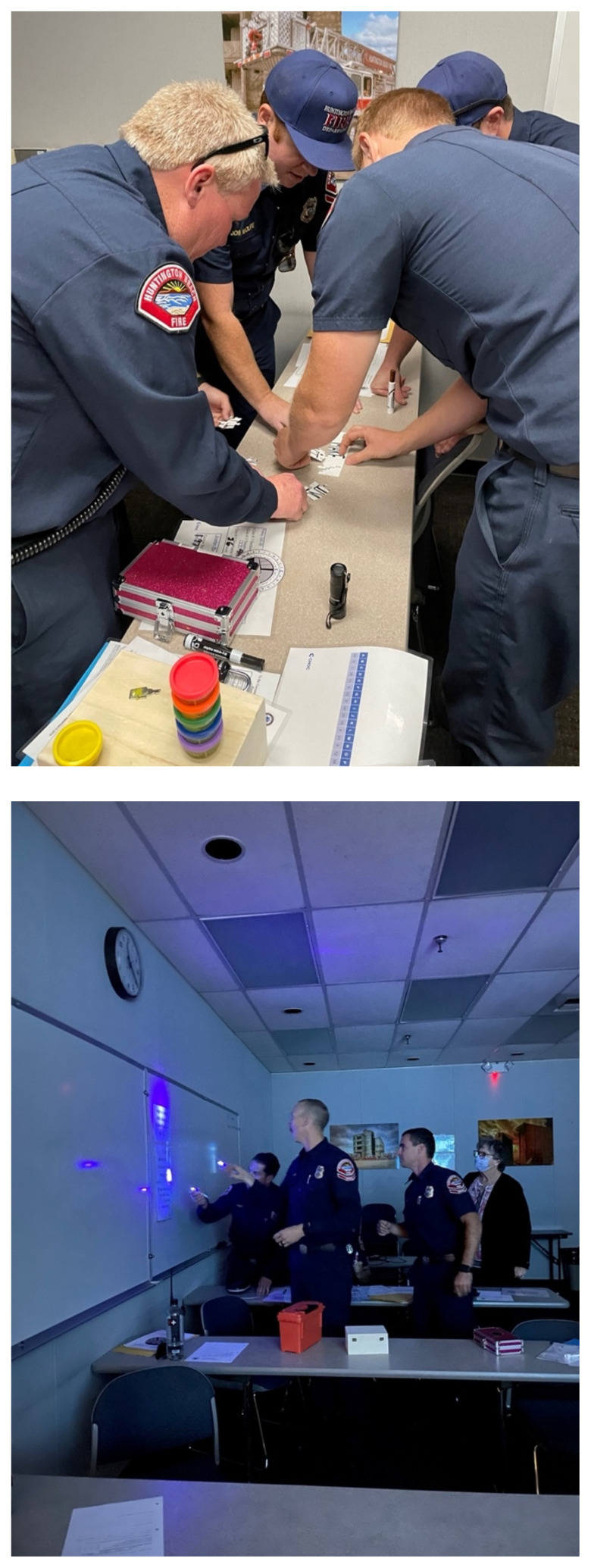
Fire department personnel interacting with puzzle number 5 and 2.
