# Supplementary material for: Child Maltreatment Education: Utilizing an Escape Room Activity to Engage Learners on a Sensitive Topic
Source: J Educ Teach Emerg Med. 2023 Jan 31;8(1):SG1–SG21. doi: 10.21980/J84H1C (PMC10332768; doi:10.21980/J84H1C)
Supplement: Supplementary file 1 [file jetem-8-1-sg1-appendixA.pptx]

## Slide 1
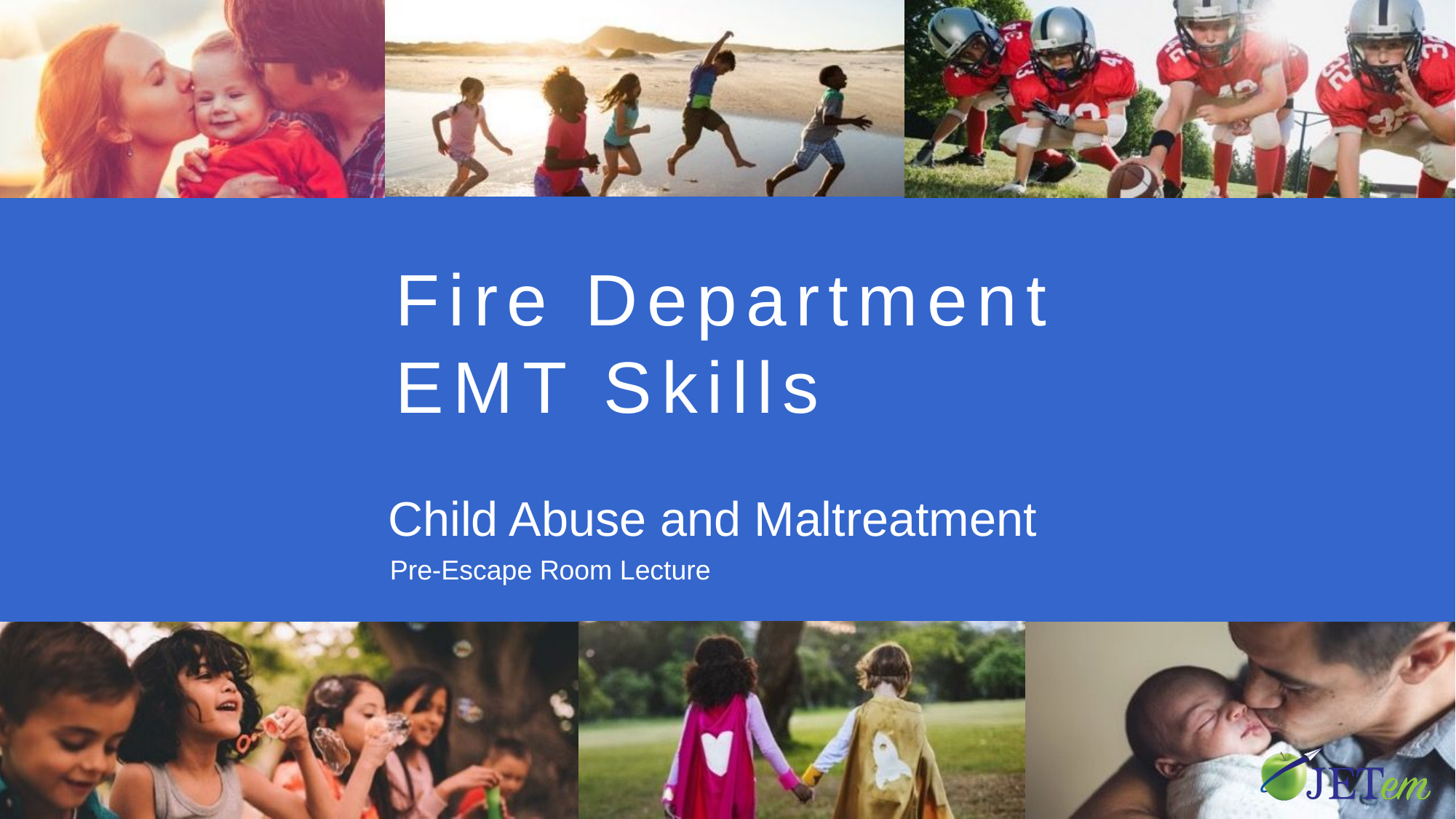

# Fire Department EMT Skills
Child Abuse and Maltreatment
Pre-Escape Room Lecture

## Slide 2
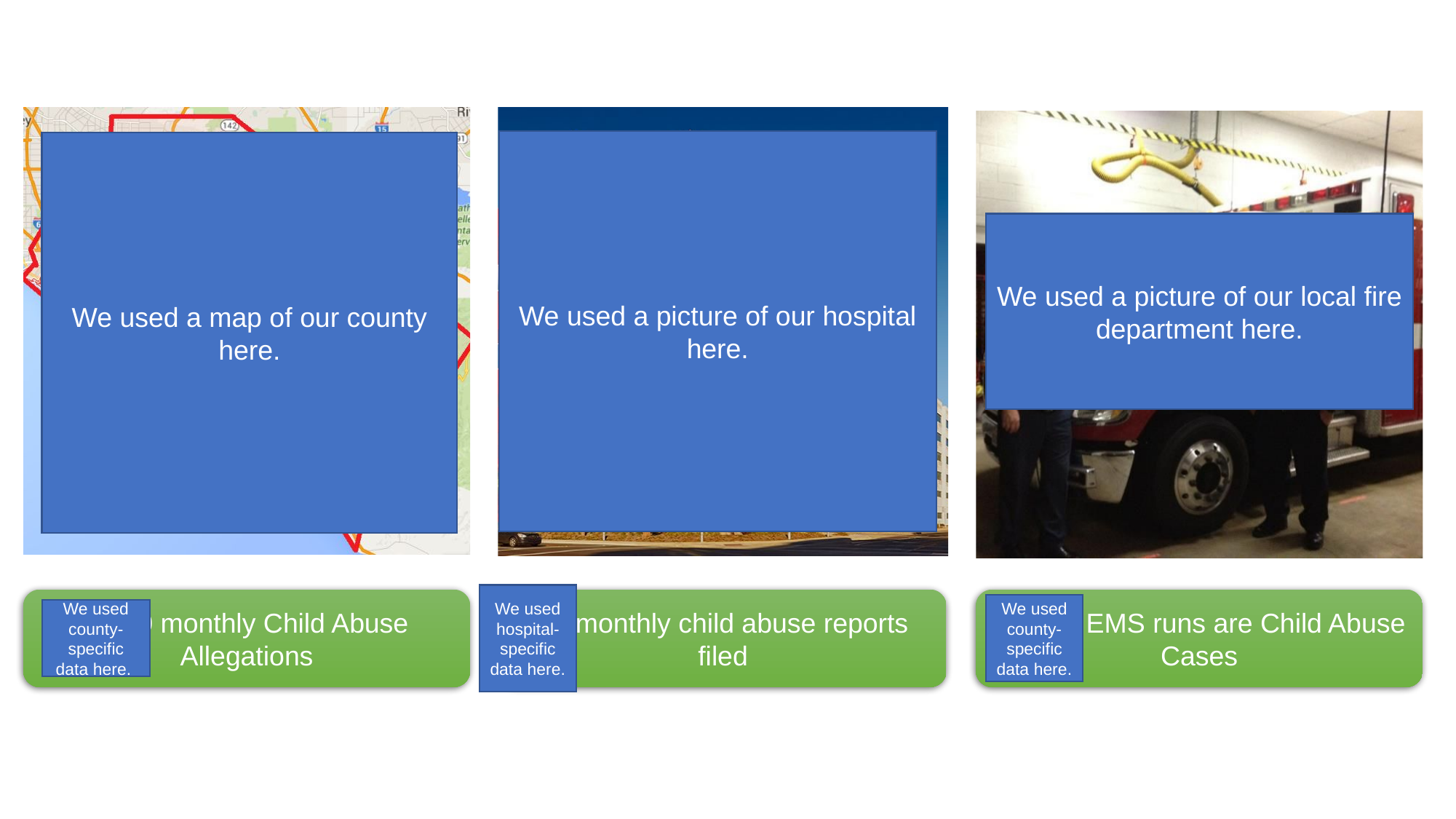

We used a picture of our hospital here.
We used a map of our county here.
We used a picture of our local fire department here.
We used hospital-specific data here.
2,500 monthly Child Abuse Allegations
80 monthly child abuse reports filed
~10-15 EMS runs are Child Abuse Cases
We used county-specific data here.
We used county-specific data here.

## Slide 3
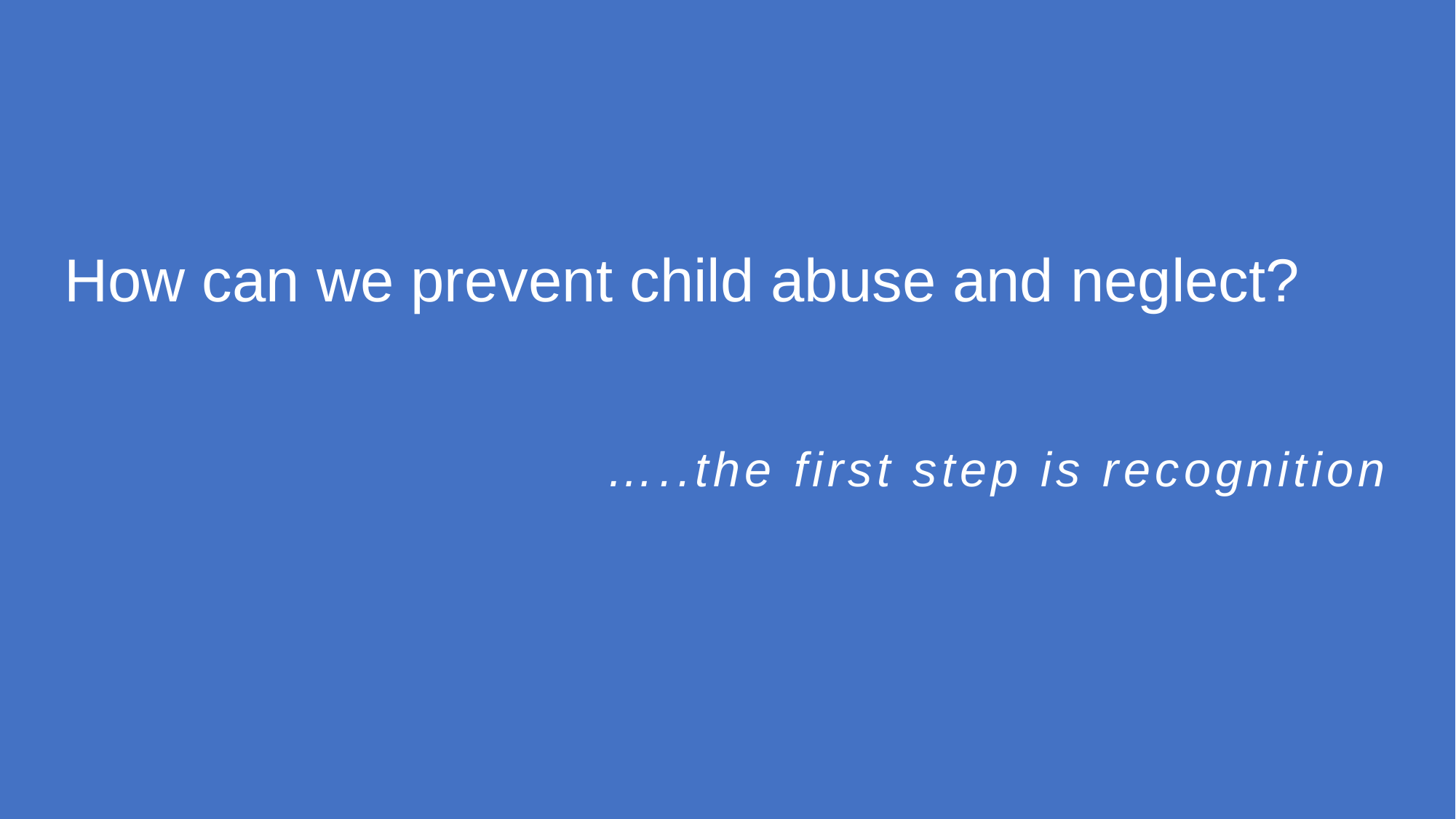

# How can we prevent child abuse and neglect?
…..the first step is recognition

## Slide 4
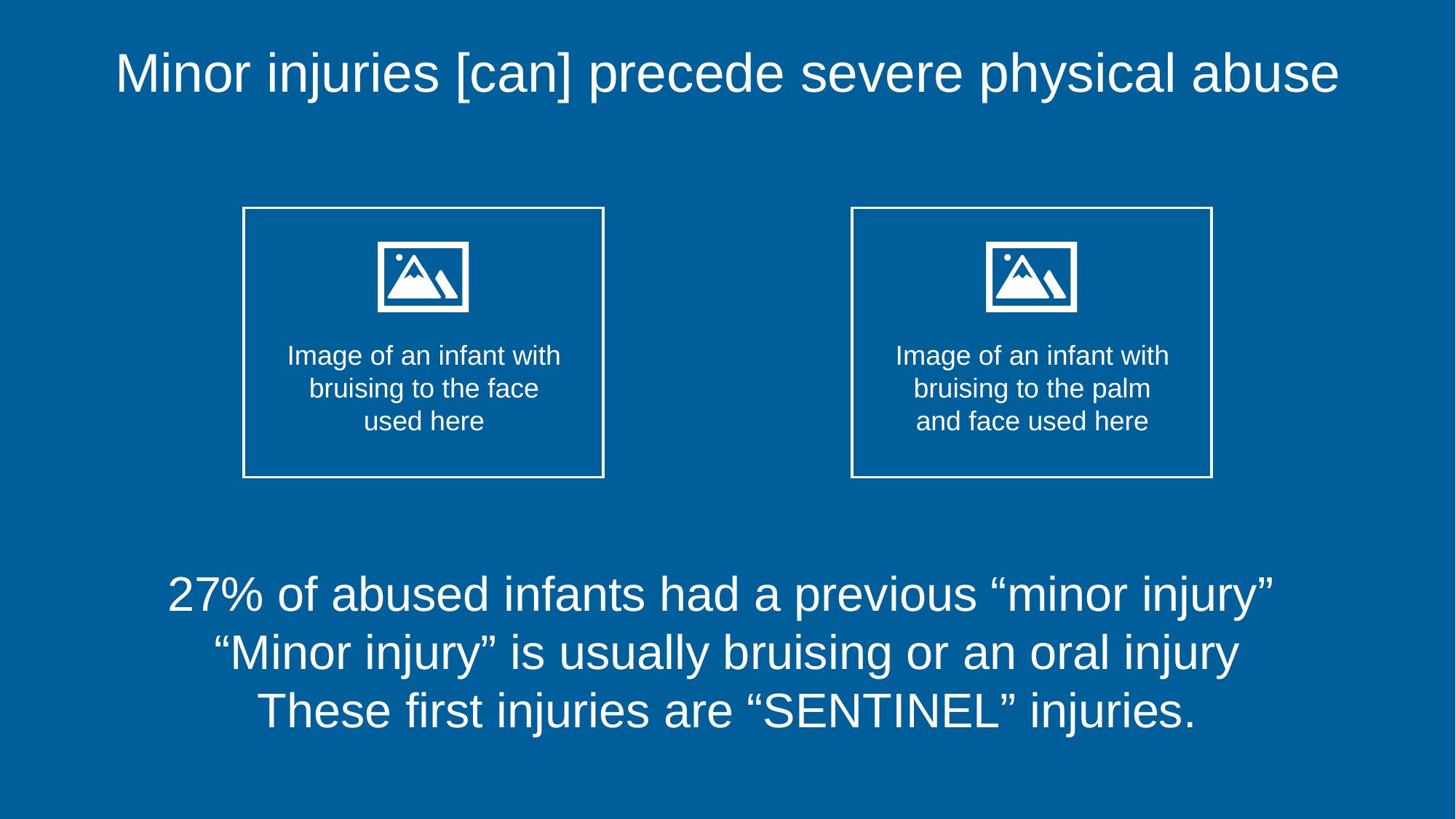

Minor injuries [can] precede severe physical abuse
Image of an infant with bruising to the face used here
Image of an infant with bruising to the palm and face used here
27% of abused infants had a previous “minor injury”
“Minor injury” is usually bruising or an oral injury
These first injuries are “SENTINEL” injuries.

## Slide 5
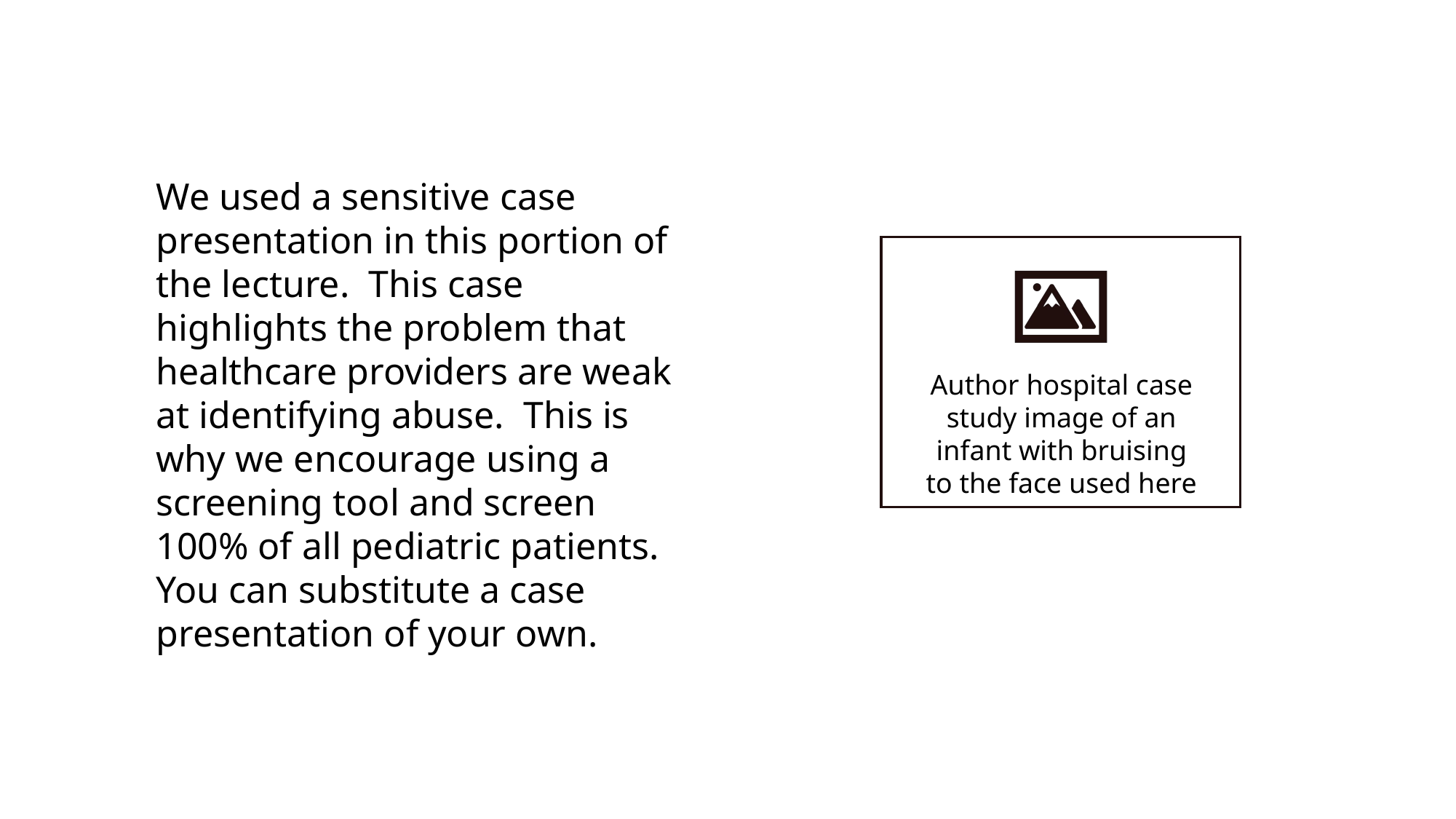

We used a sensitive case presentation in this portion of the lecture. This case highlights the problem that healthcare providers are weak at identifying abuse. This is why we encourage using a screening tool and screen 100% of all pediatric patients. You can substitute a case presentation of your own.
Author hospital case study image of an infant with bruising to the face used here

## Slide 6
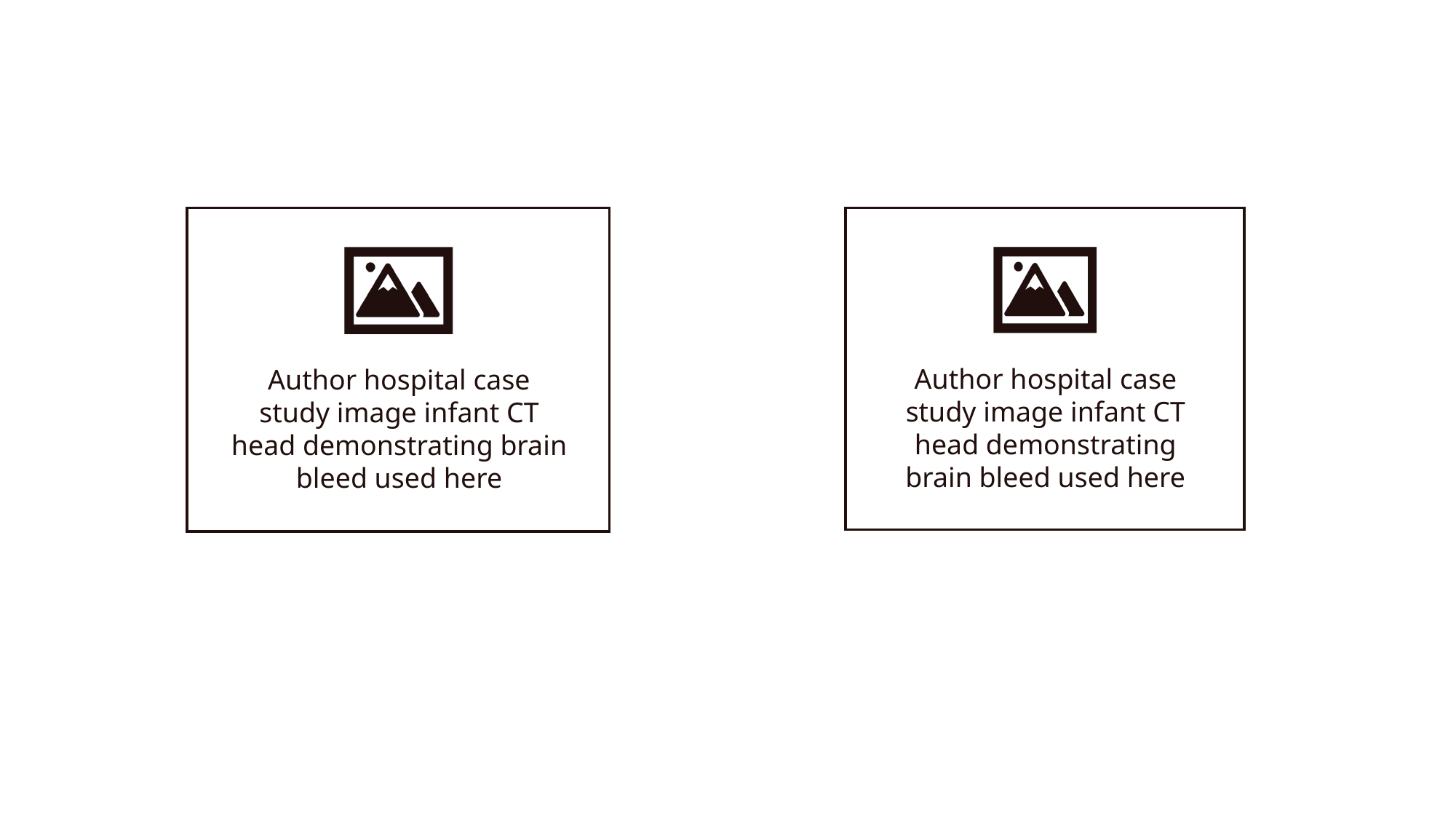

Author hospital case study image infant CT head demonstrating brain bleed used here
Author hospital case study image infant CT head demonstrating brain bleed used here
Source: [Hospital files]

## Slide 7
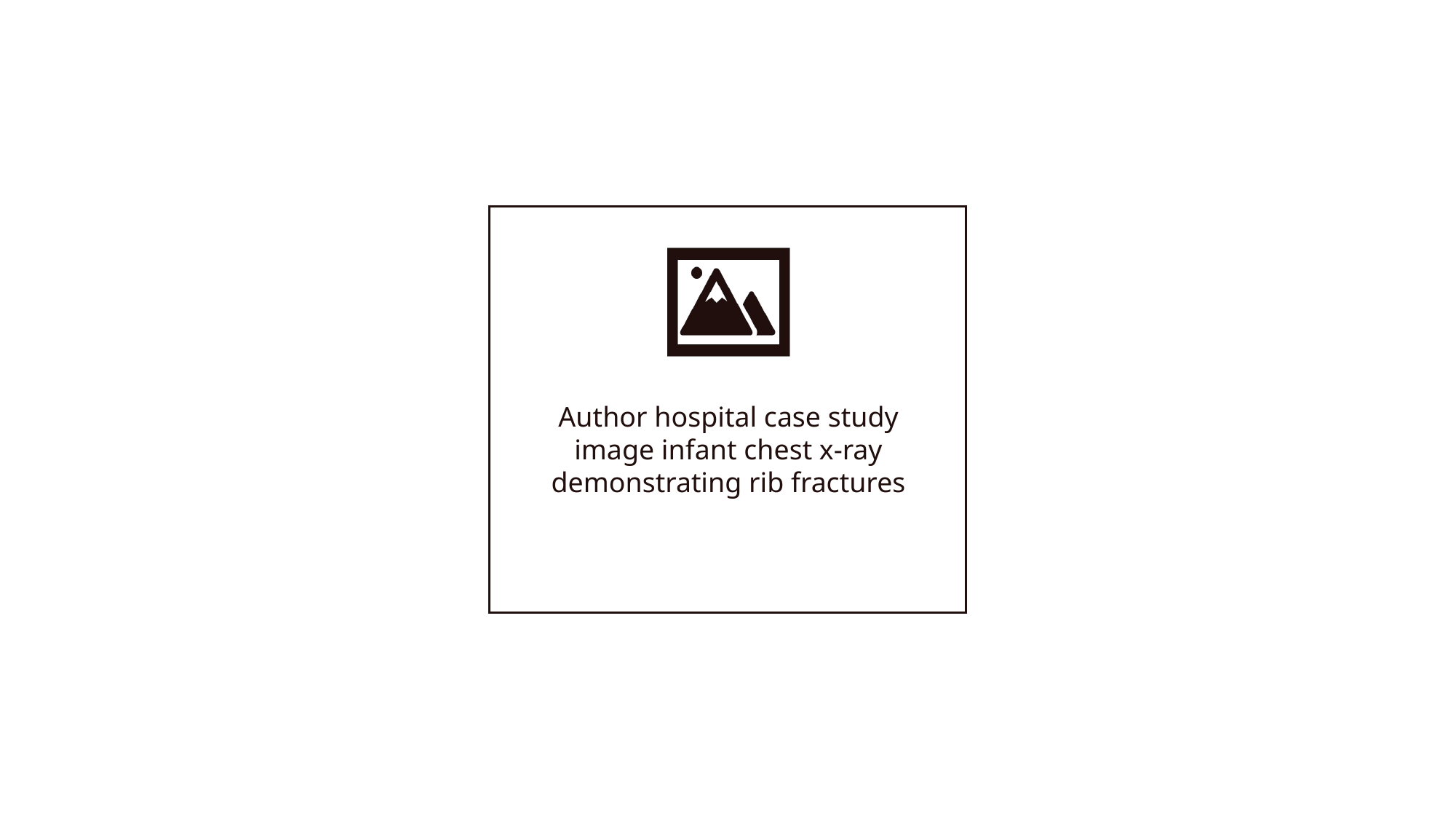

Author hospital case study image infant chest x-ray demonstrating rib fractures

## Slide 8
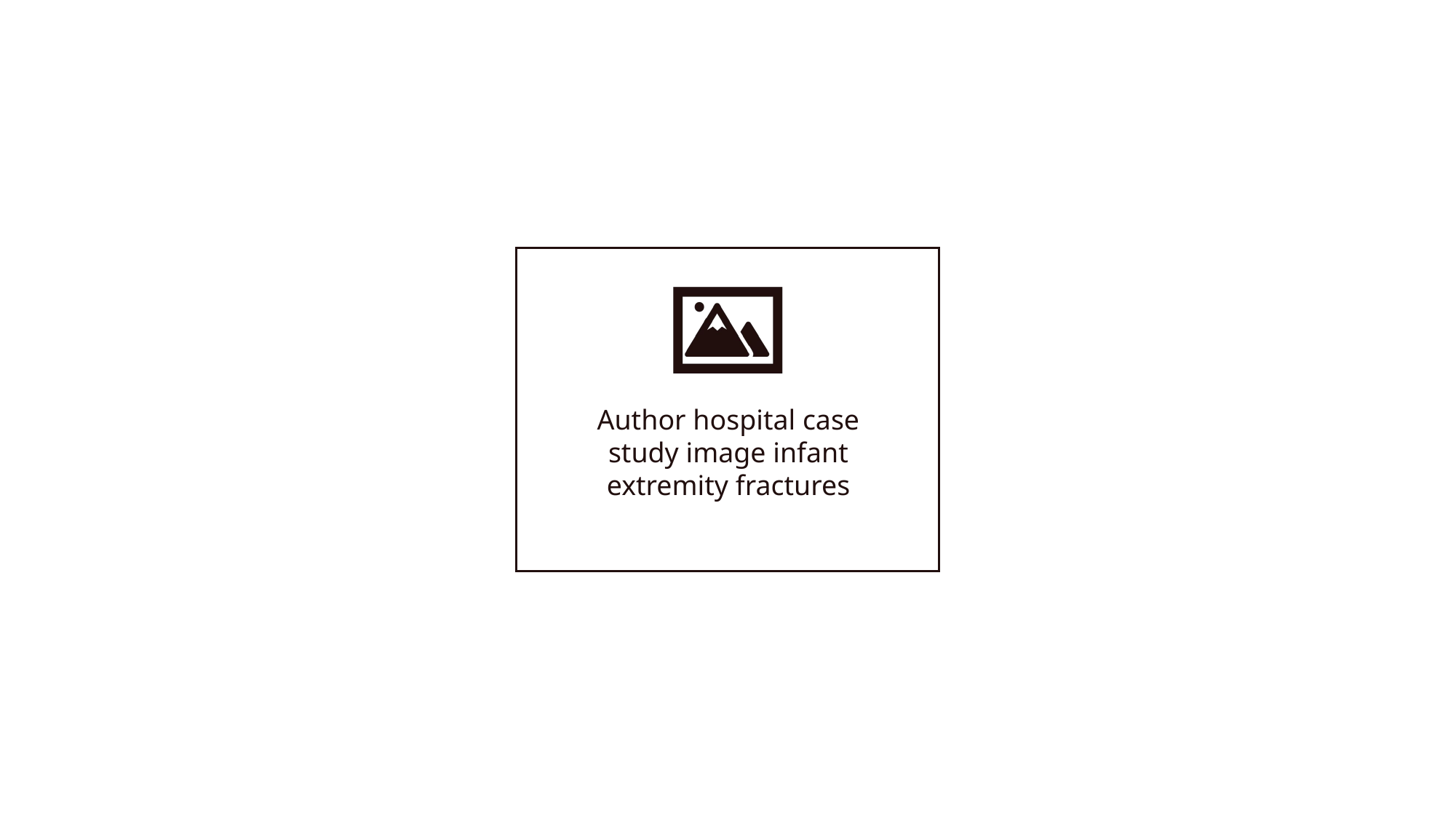

Author hospital case study image infant extremity fractures

## Slide 9
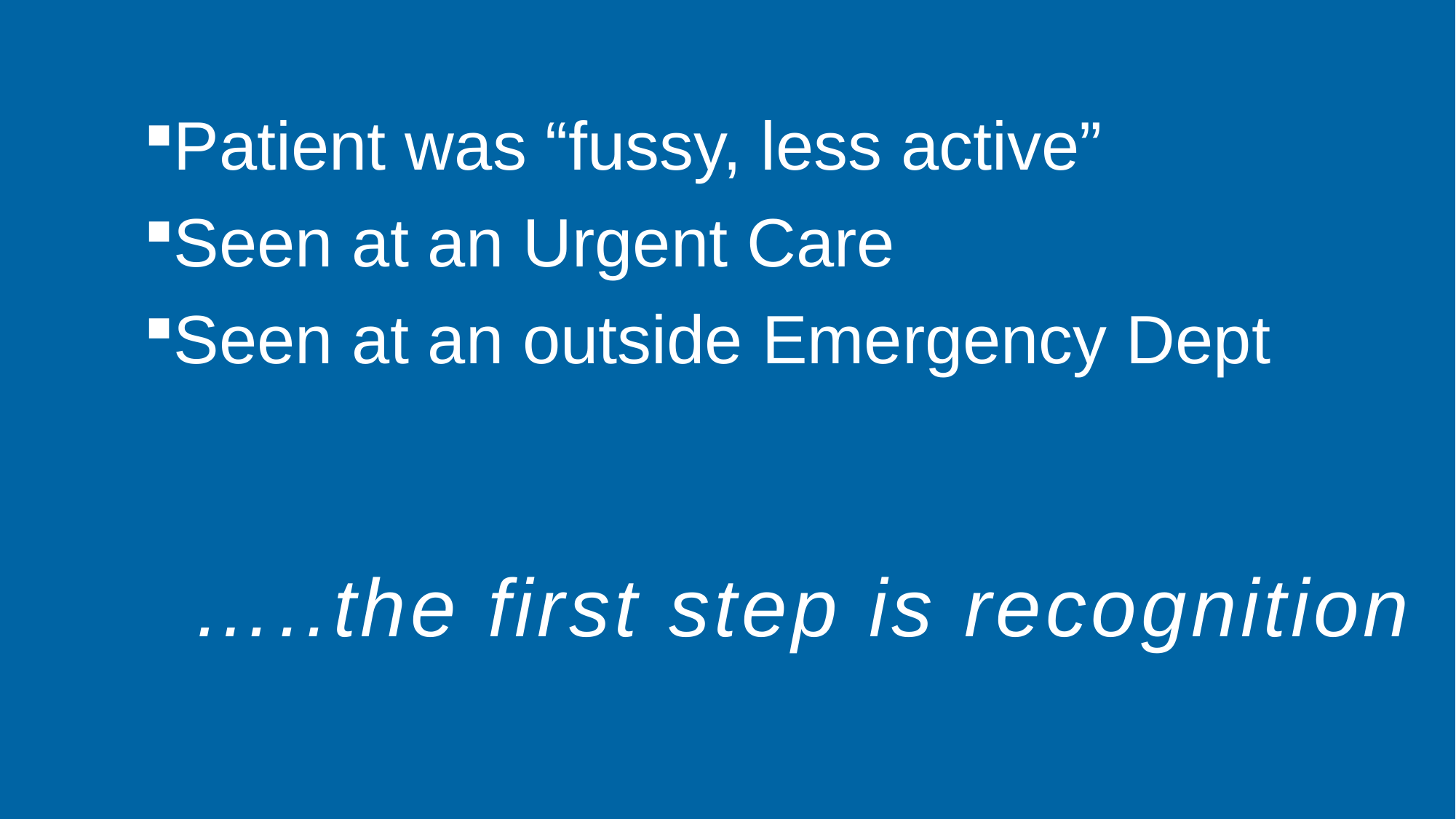

Patient was “fussy, less active”
Seen at an Urgent Care
Seen at an outside Emergency Dept
…..the first step is recognition

## Slide 10
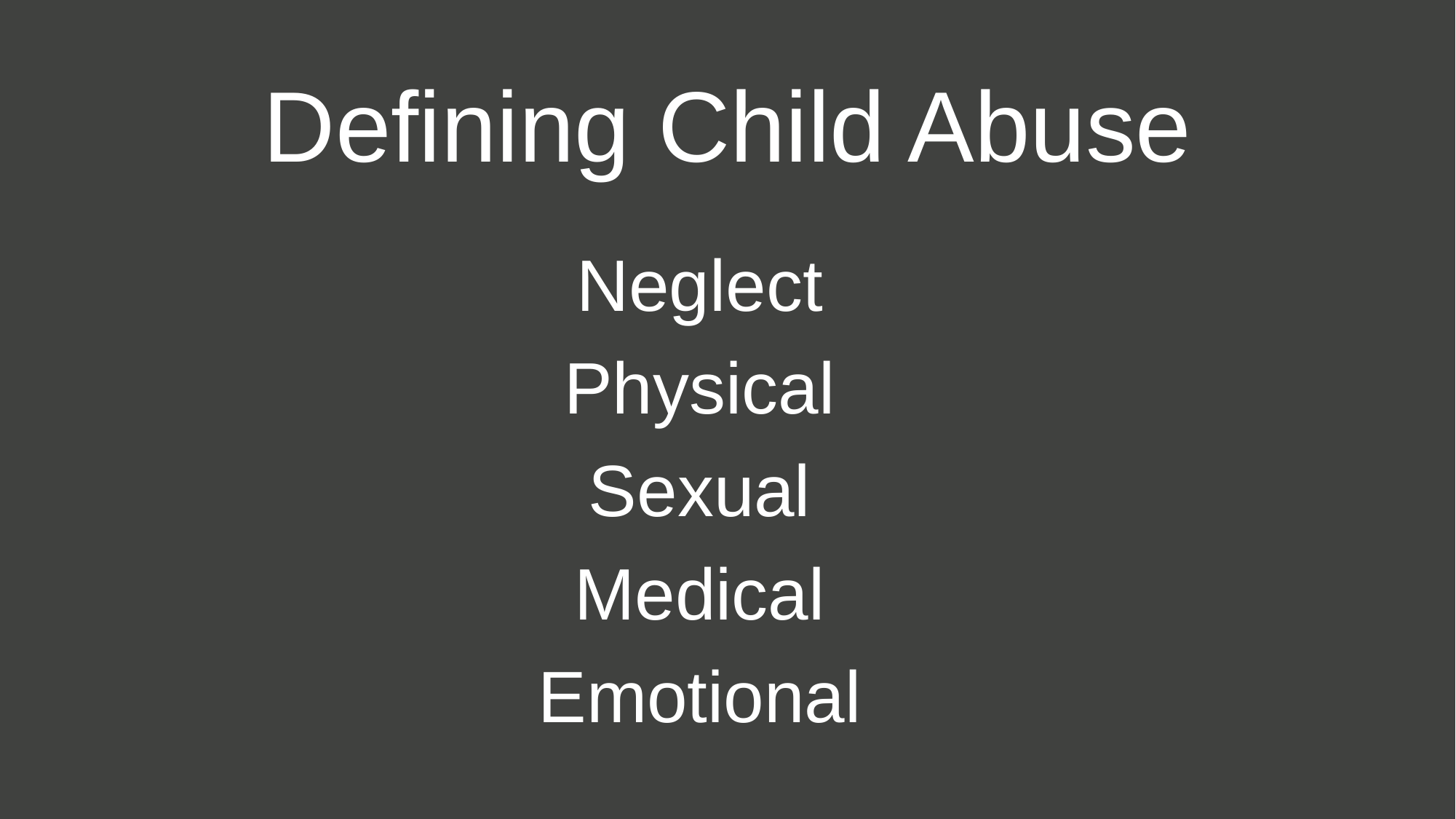

Defining Child Abuse
Neglect
Physical
Sexual
Medical
Emotional

## Slide 11
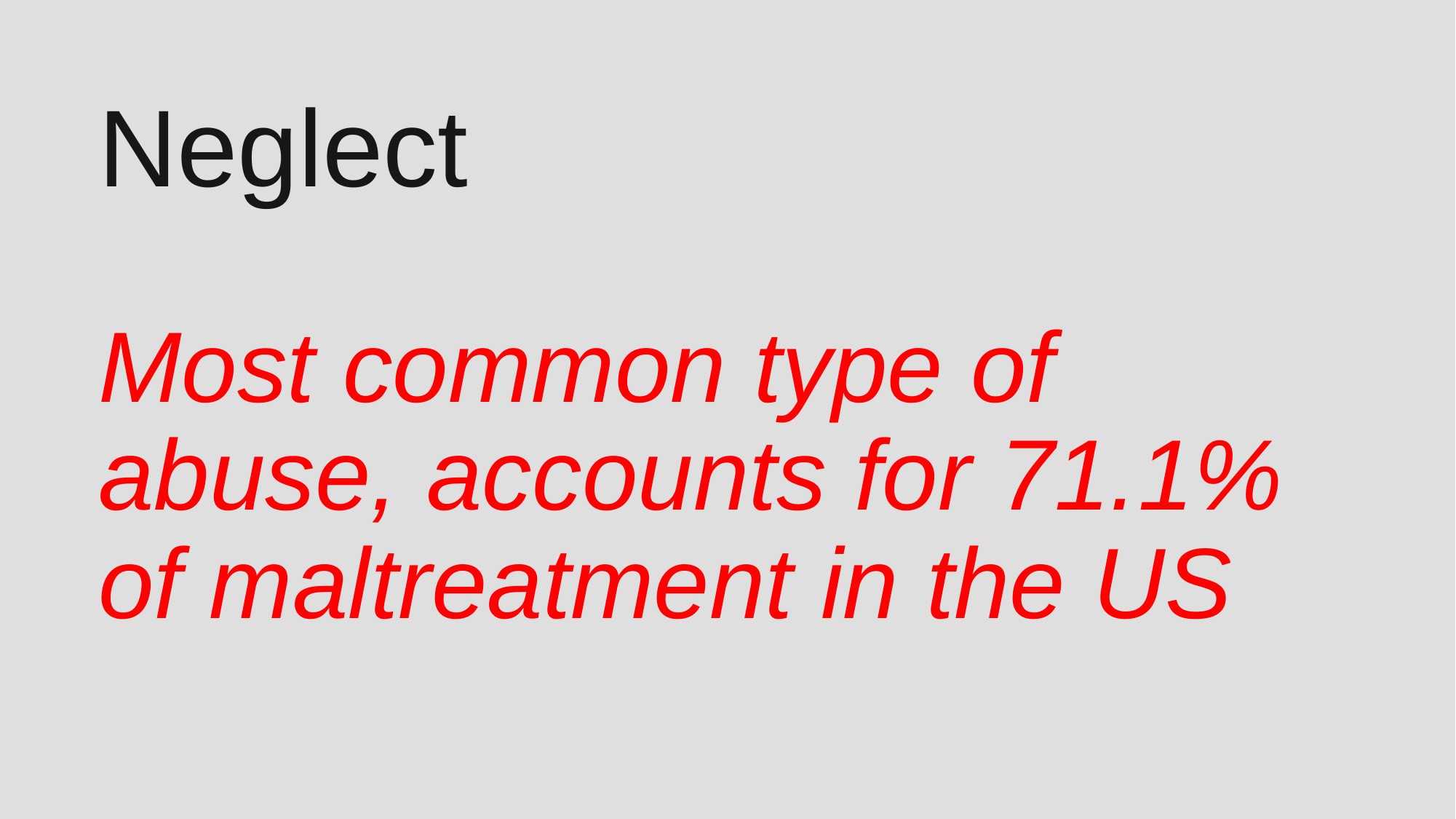

Neglect
Most common type of abuse, accounts for 71.1% of maltreatment in the US

## Slide 12
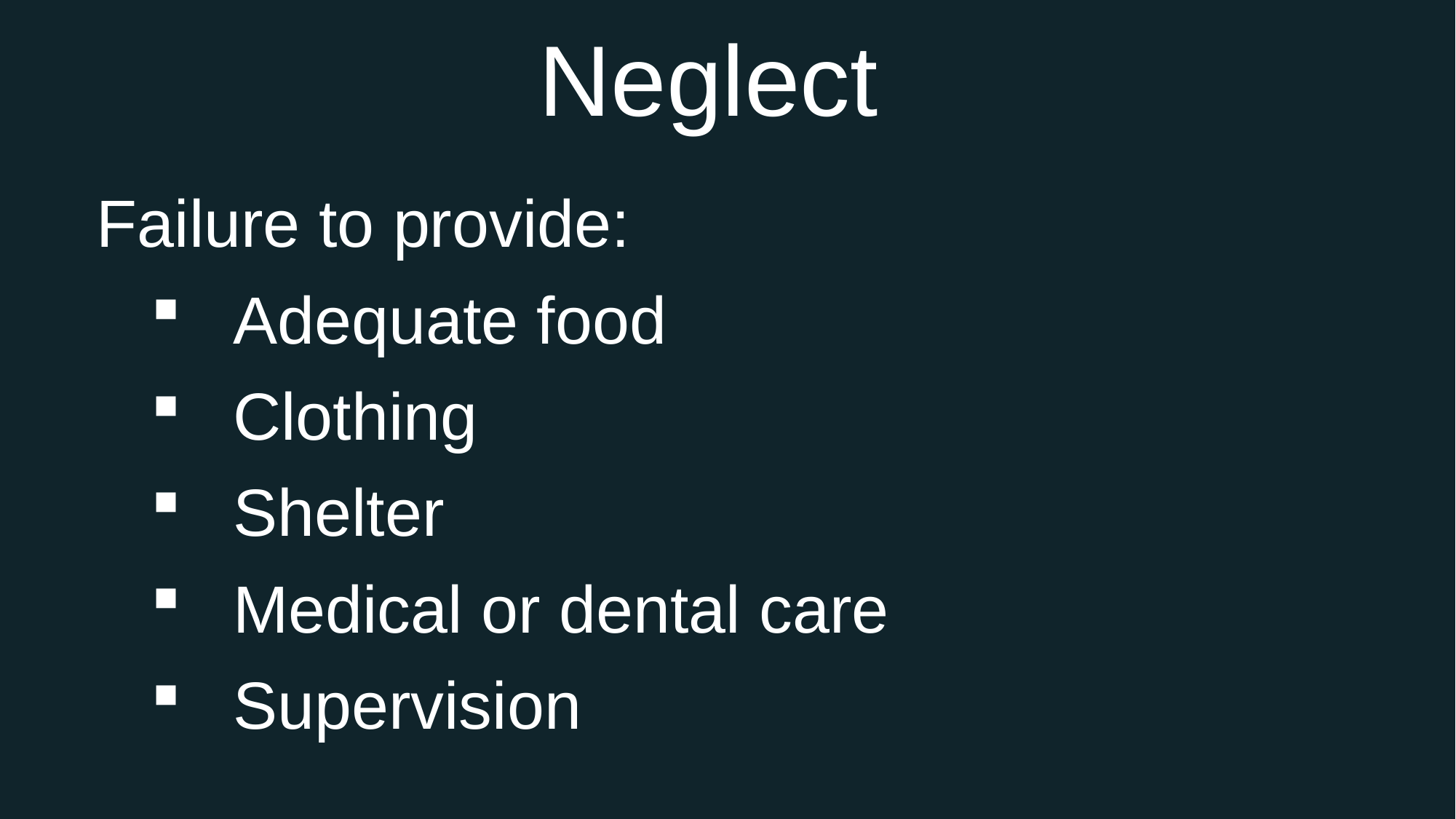

Neglect
Failure to provide:
Adequate food
Clothing
Shelter
Medical or dental care
Supervision

## Slide 13
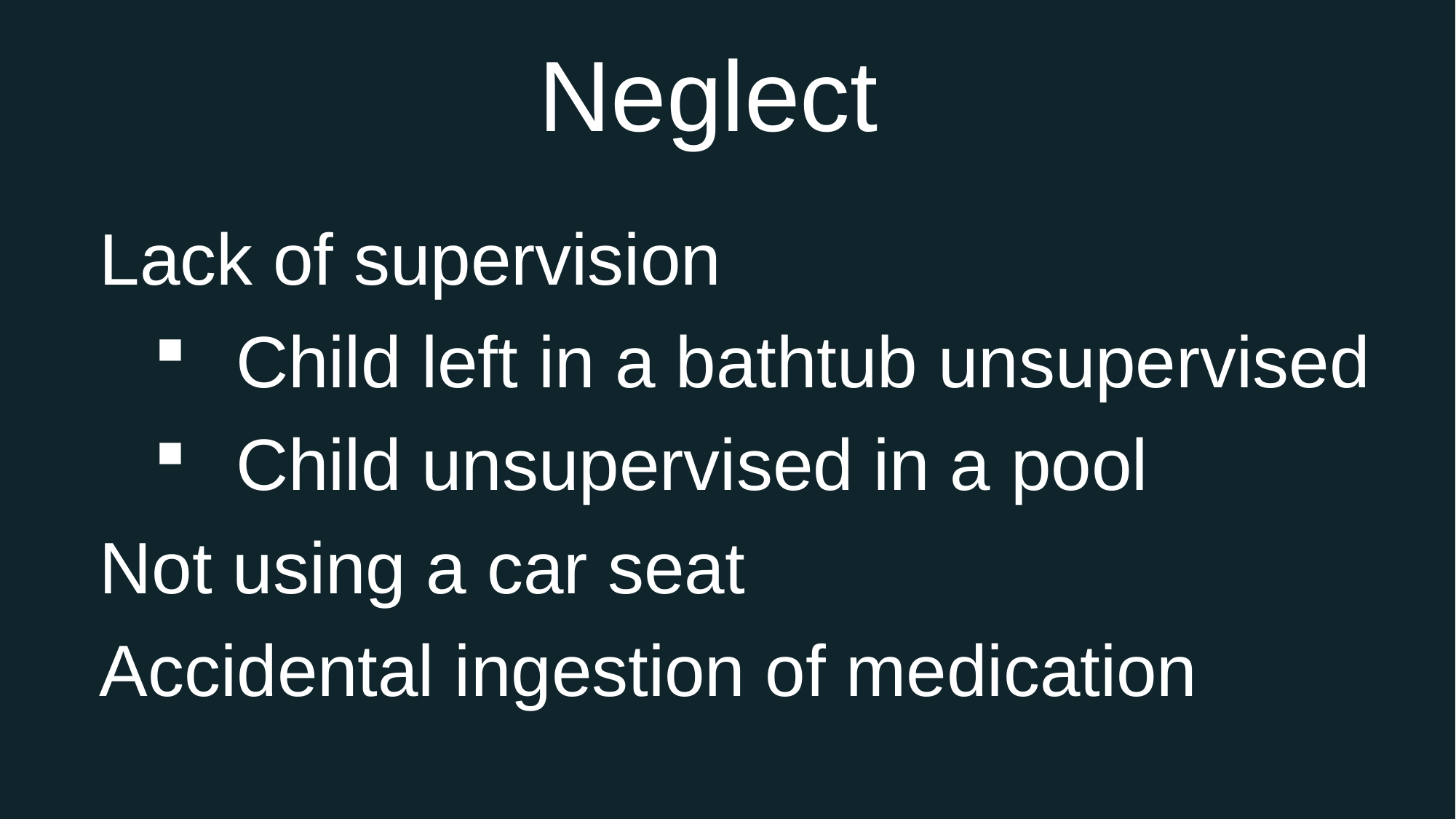

Neglect
Lack of supervision
Child left in a bathtub unsupervised
Child unsupervised in a pool
Not using a car seat
Accidental ingestion of medication

## Slide 14
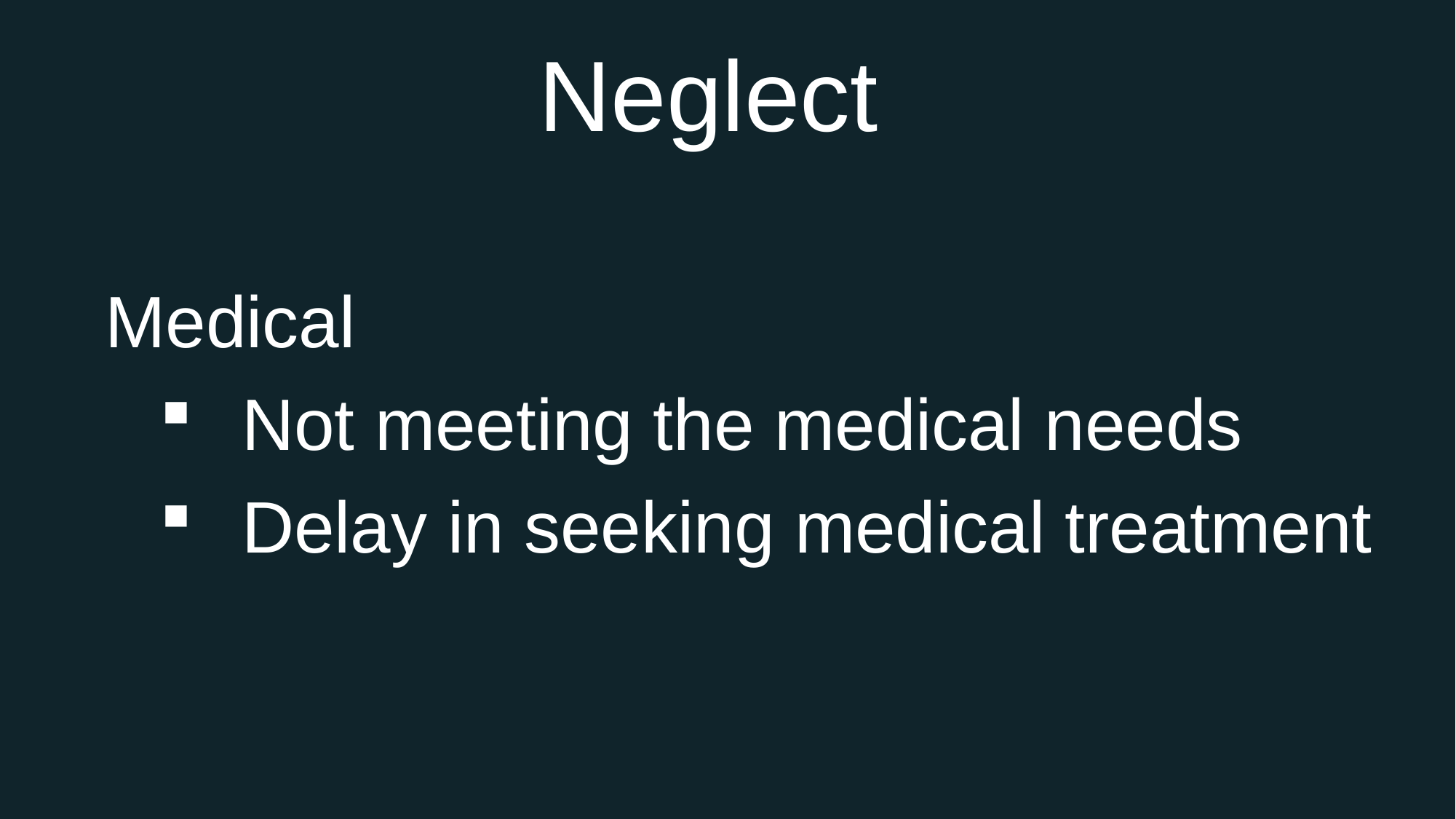

Neglect
Medical
Not meeting the medical needs
Delay in seeking medical treatment

## Slide 15
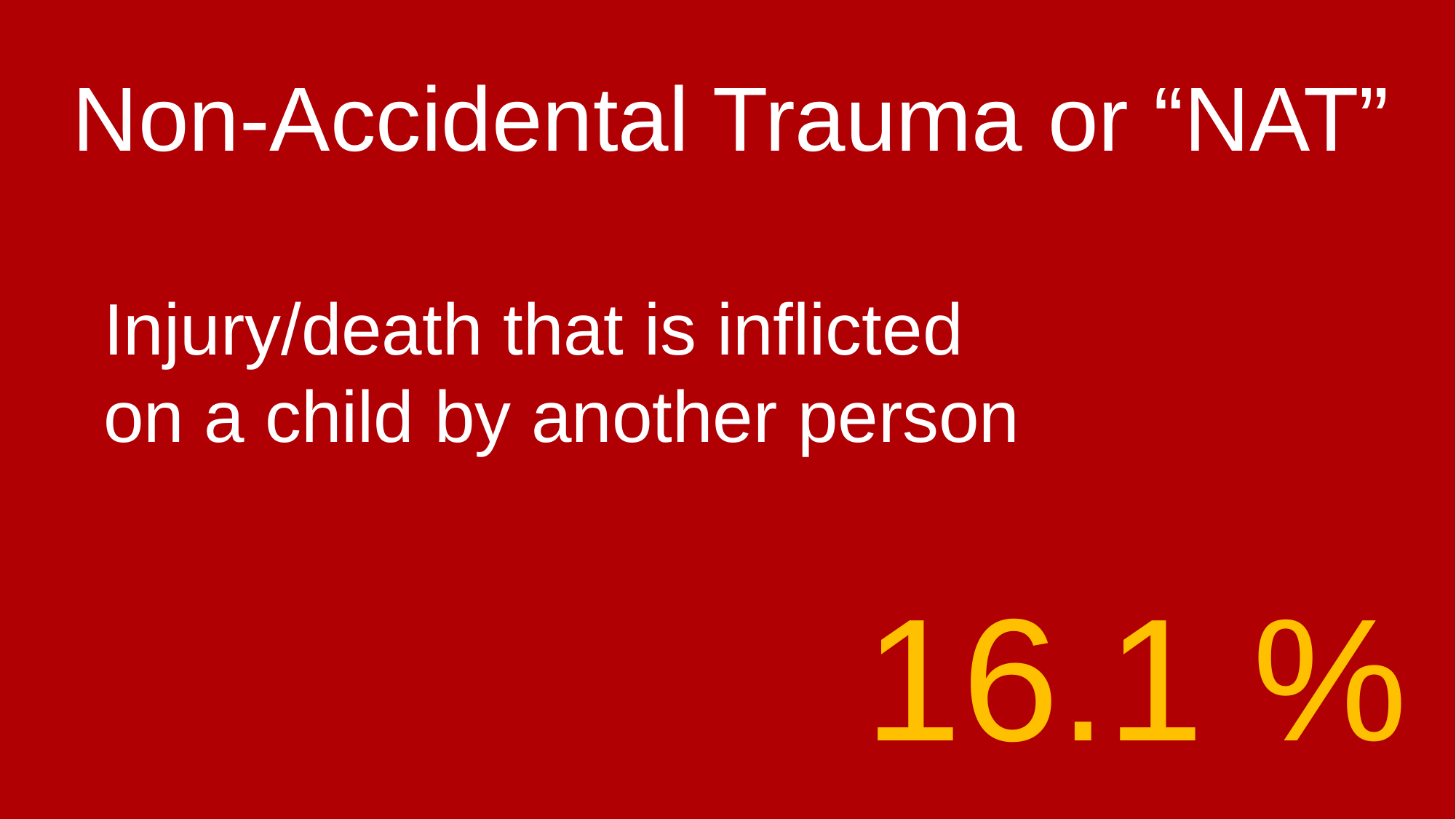

Non-Accidental Trauma or “NAT”
Injury/death that is inflicted on a child by another person
16.1 %

## Slide 16
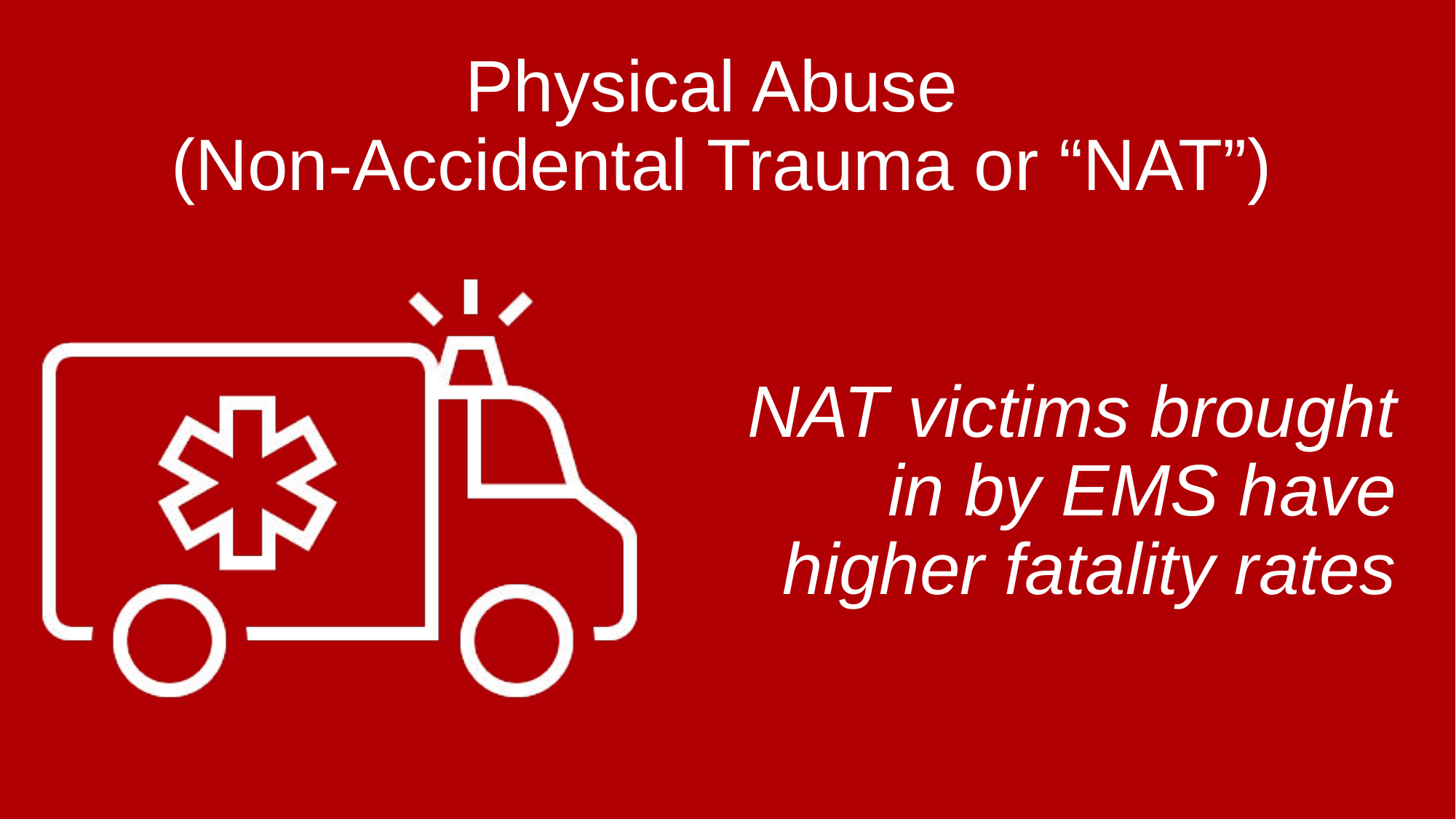

Physical Abuse
(Non-Accidental Trauma or “NAT”)
NAT victims brought in by EMS have higher fatality rates

## Slide 17
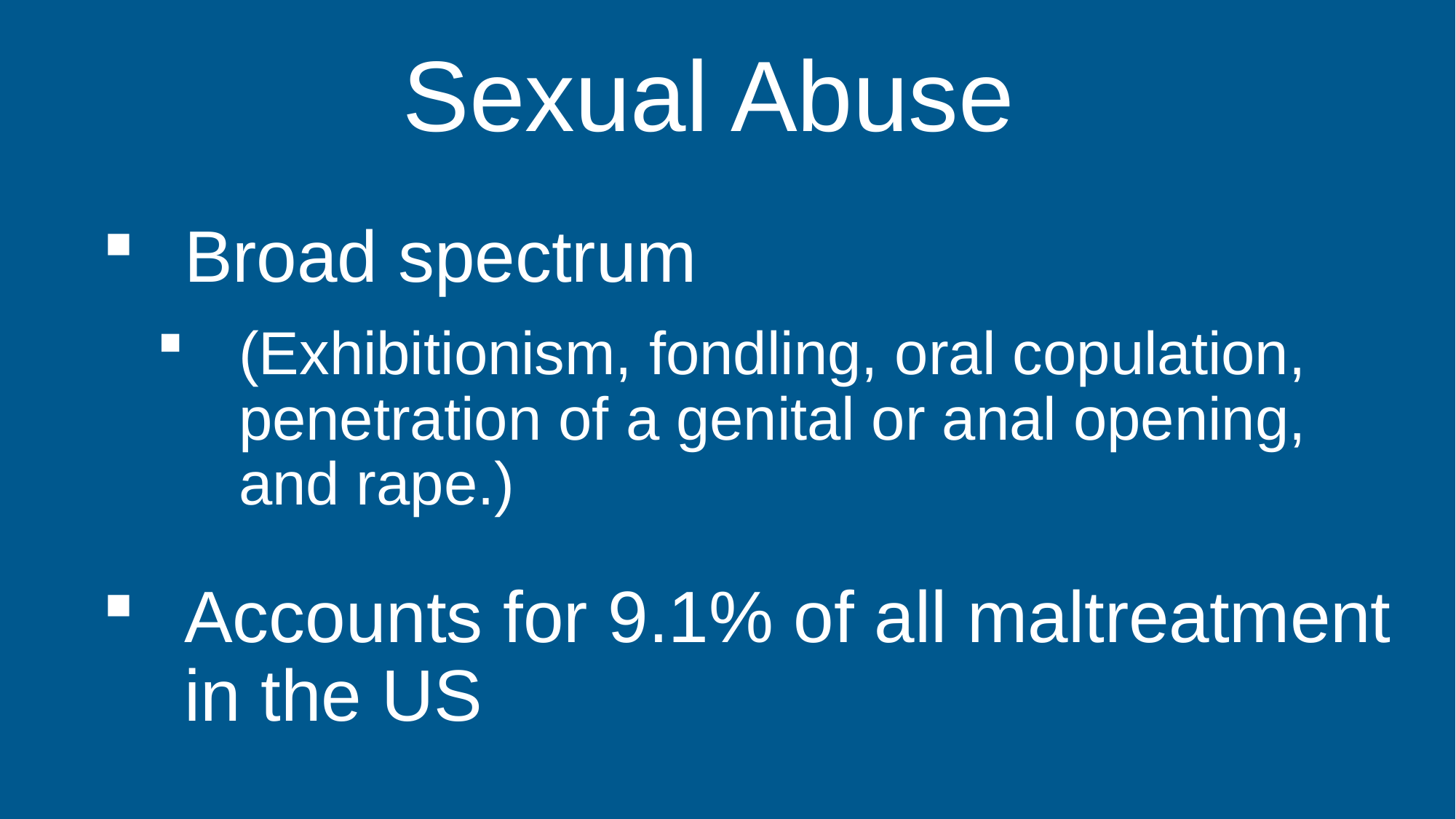

Sexual Abuse
Broad spectrum
(Exhibitionism, fondling, oral copulation, penetration of a genital or anal opening, and rape.)
Accounts for 9.1% of all maltreatment in the US

## Slide 18
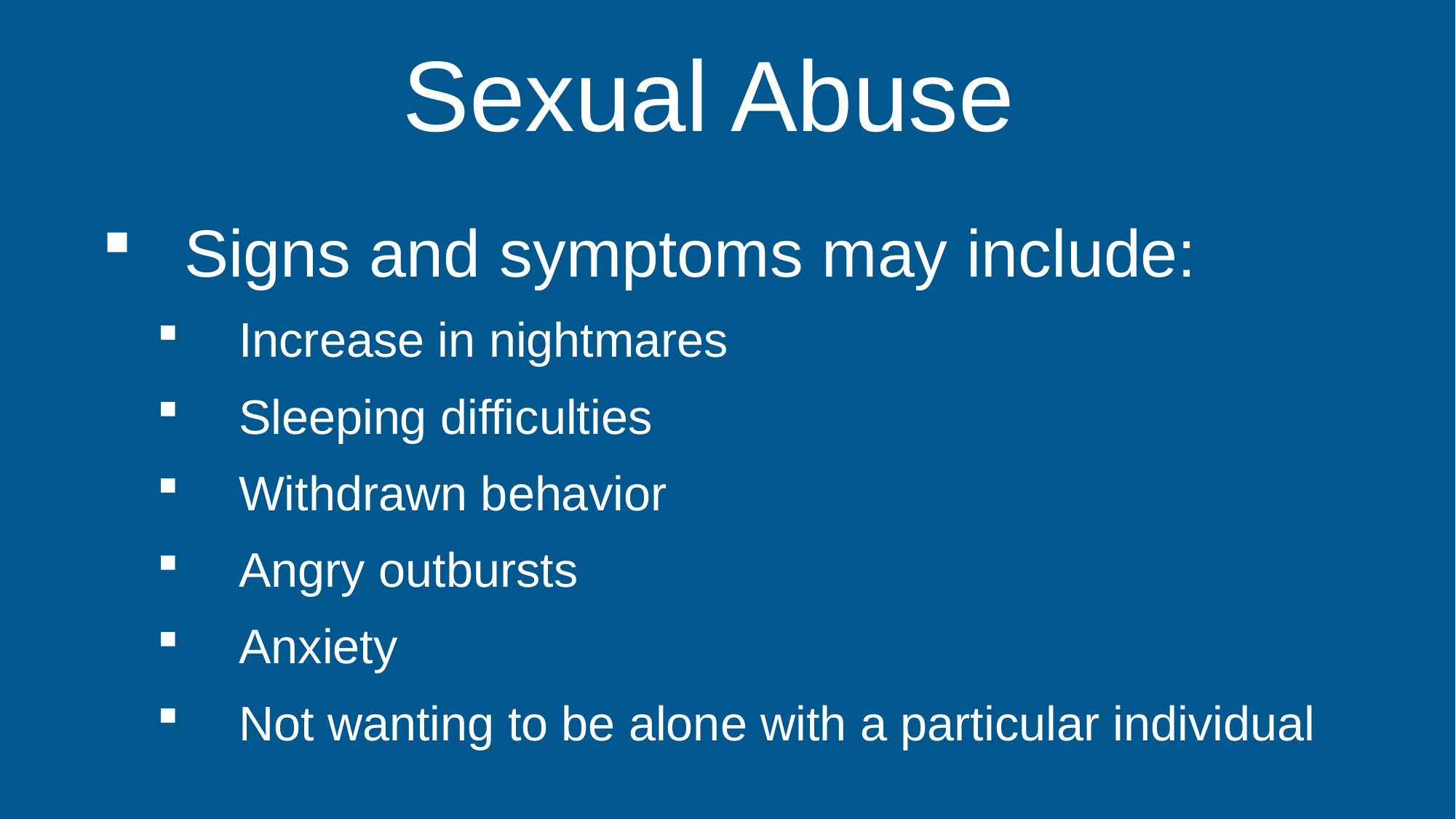

Sexual Abuse
Signs and symptoms may include:
Increase in nightmares
Sleeping difficulties
Withdrawn behavior
Angry outbursts
Anxiety
Not wanting to be alone with a particular individual

## Slide 19
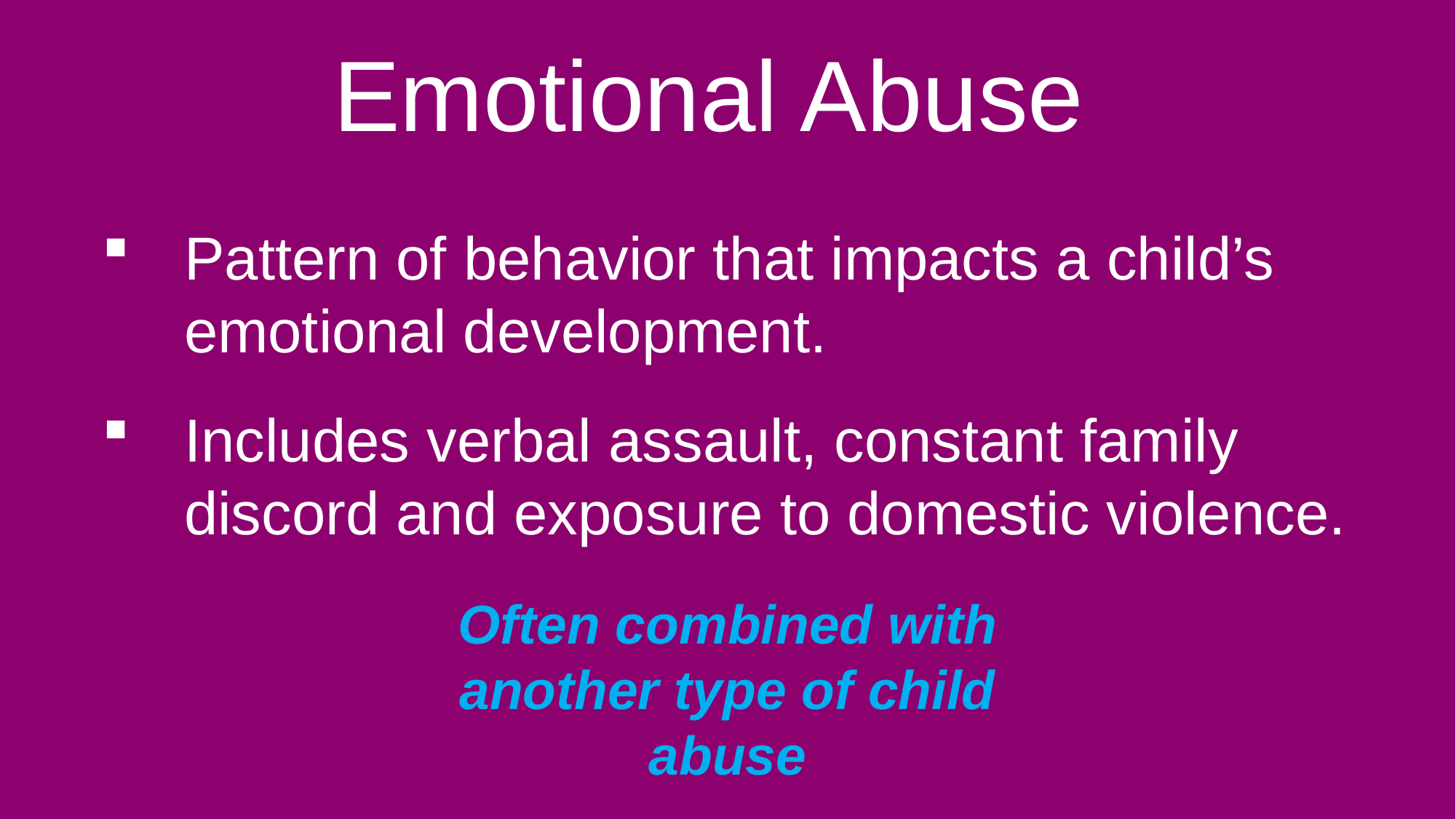

Emotional Abuse
Pattern of behavior that impacts a child’s emotional development.
Includes verbal assault, constant family discord and exposure to domestic violence.
Often combined with another type of child abuse

## Slide 20
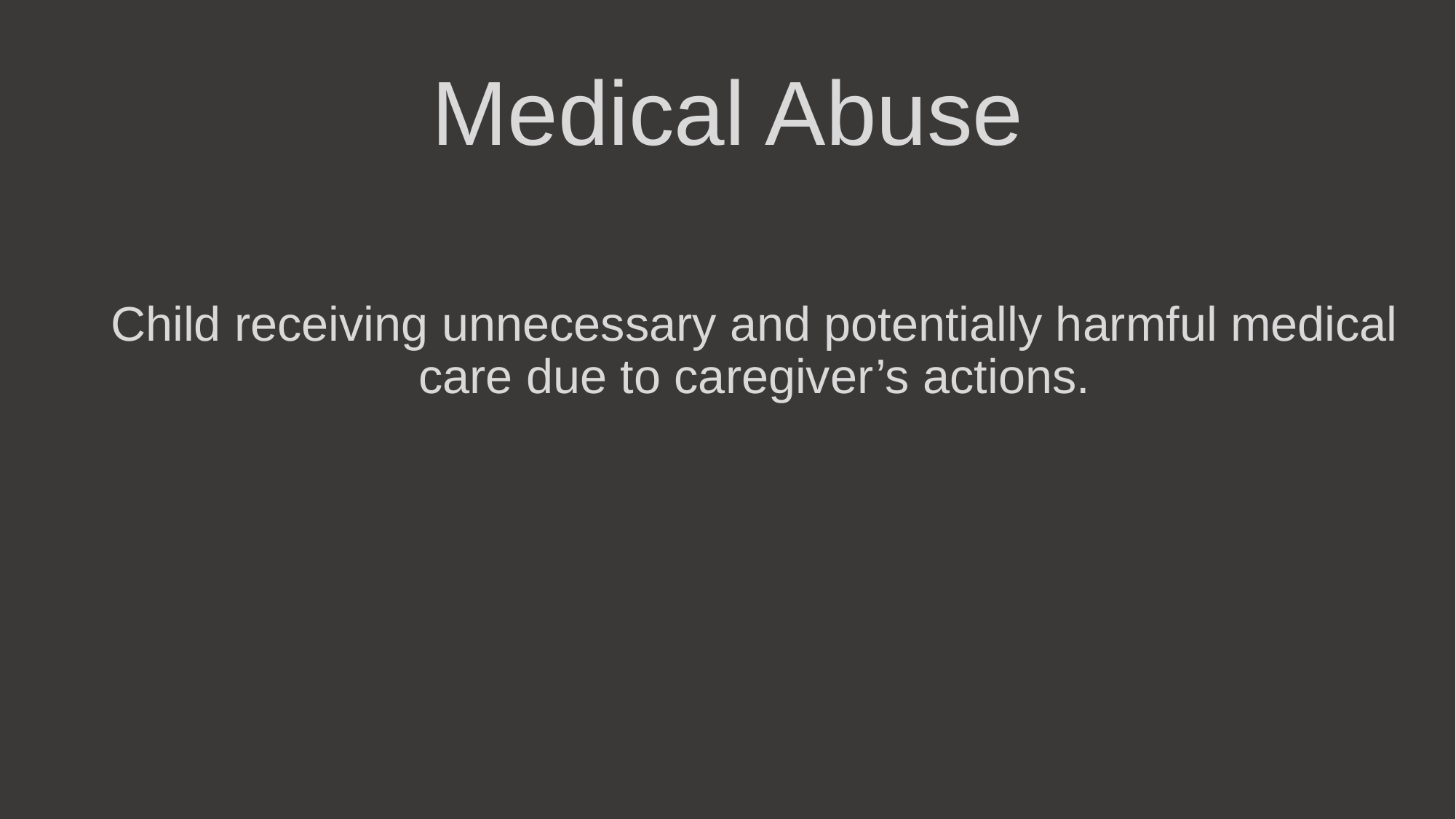

# Medical Abuse
Child receiving unnecessary and potentially harmful medical care due to caregiver’s actions.

## Slide 21
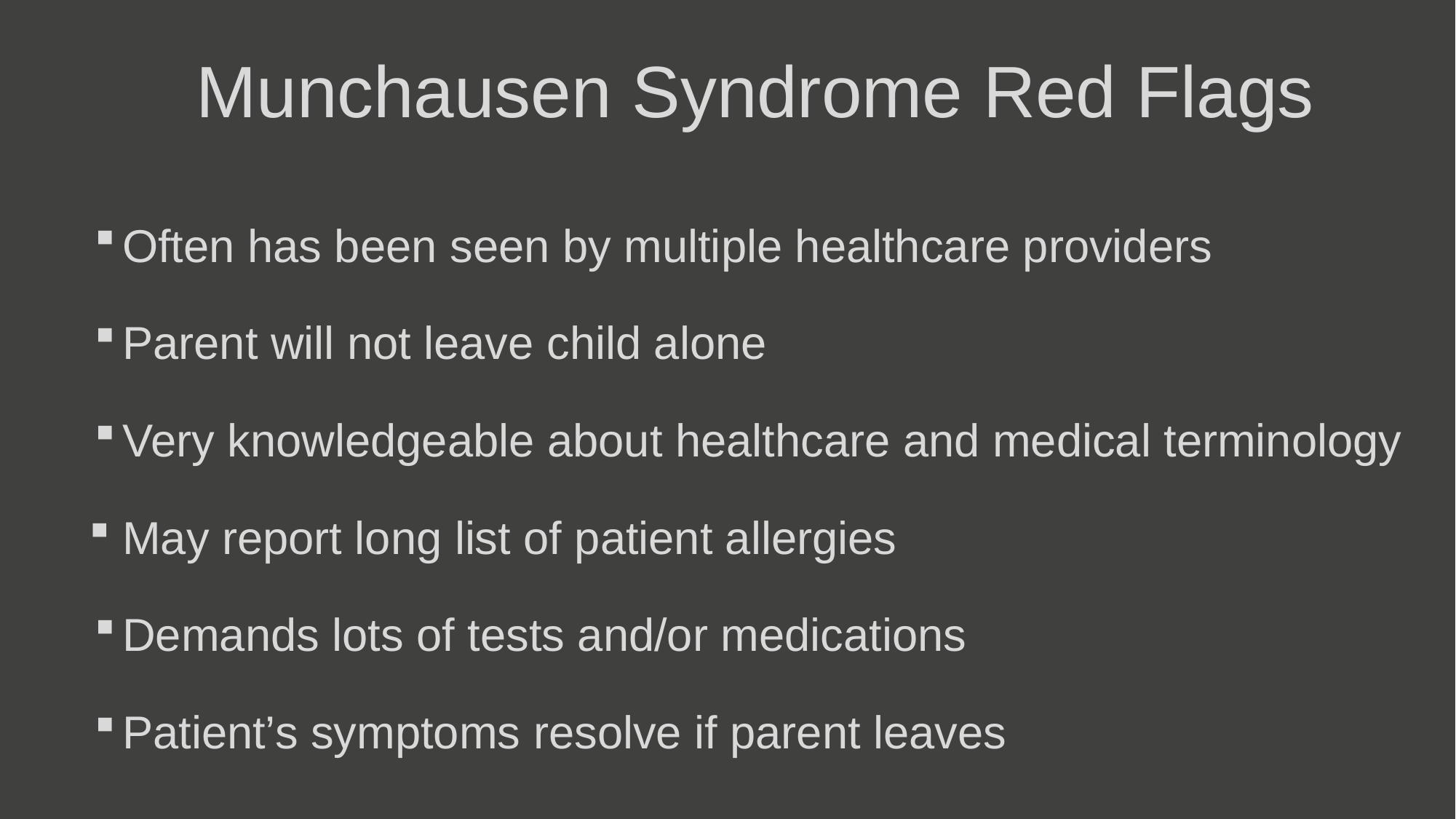

Munchausen Syndrome Red Flags
Often has been seen by multiple healthcare providers
Parent will not leave child alone
Very knowledgeable about healthcare and medical terminology
May report long list of patient allergies
Demands lots of tests and/or medications
Patient’s symptoms resolve if parent leaves

## Slide 22
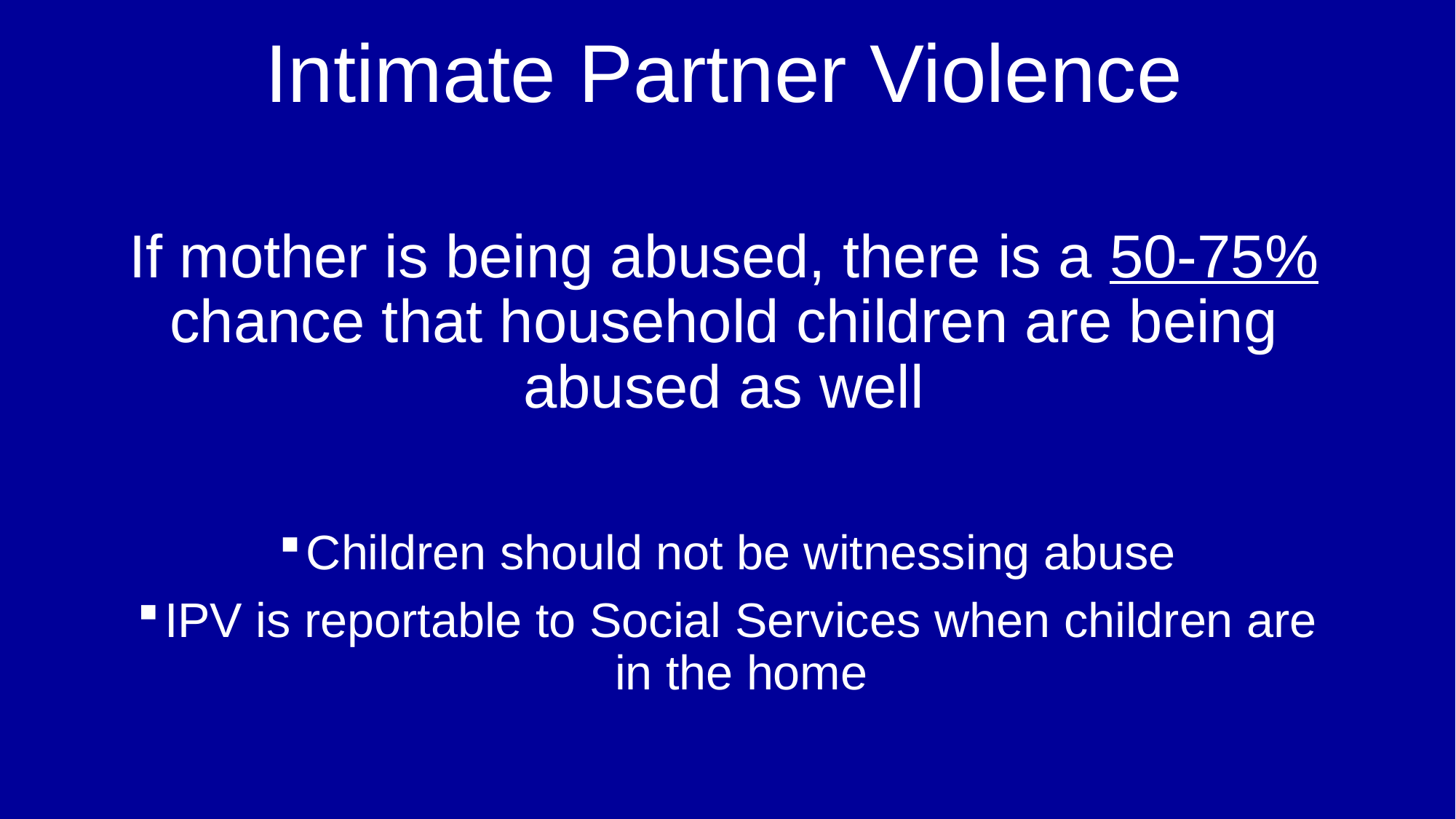

# Intimate Partner Violence
If mother is being abused, there is a 50-75% chance that household children are being abused as well
Children should not be witnessing abuse
IPV is reportable to Social Services when children are in the home

## Slide 23
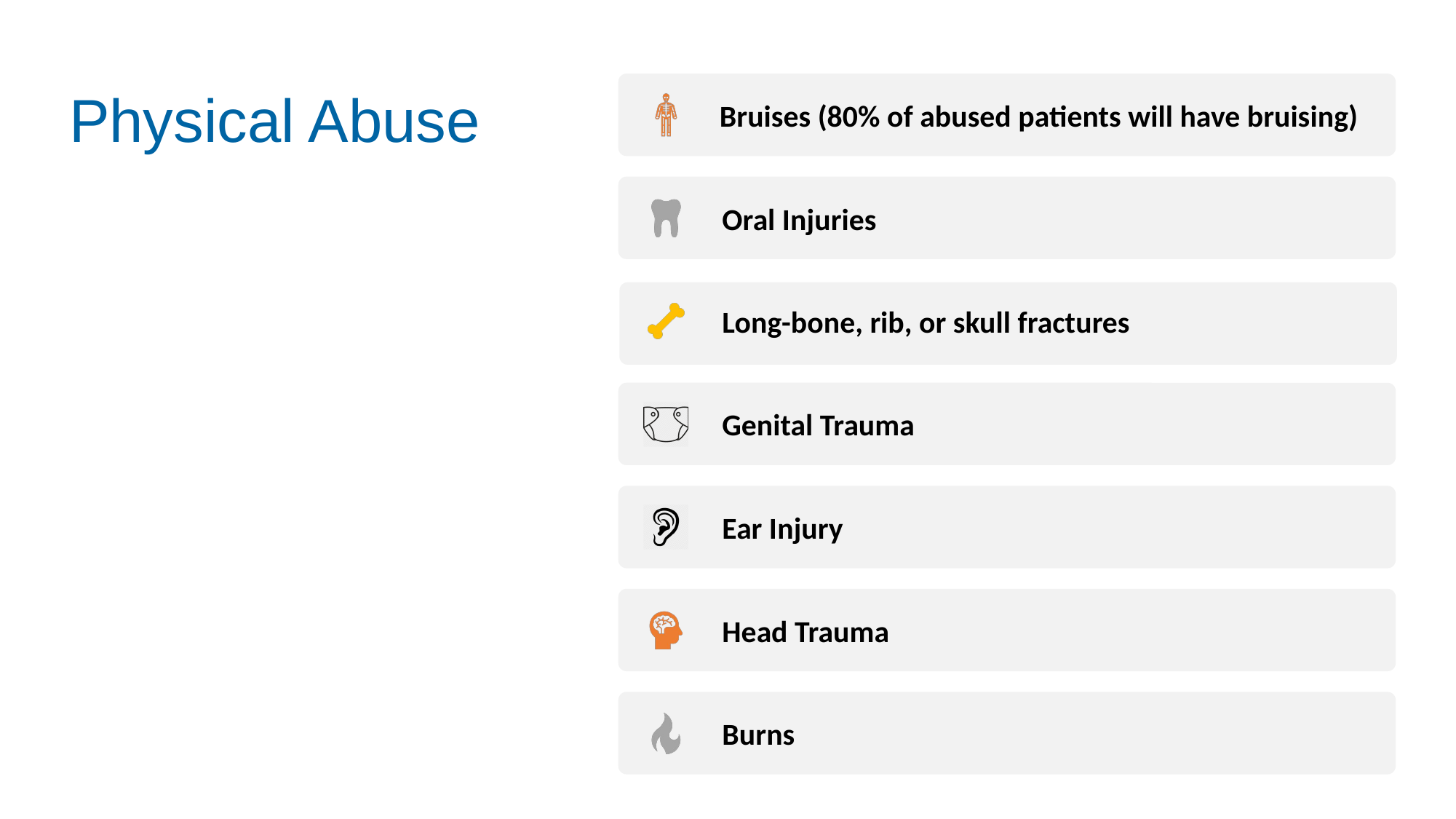

# Physical Abuse

## Slide 24
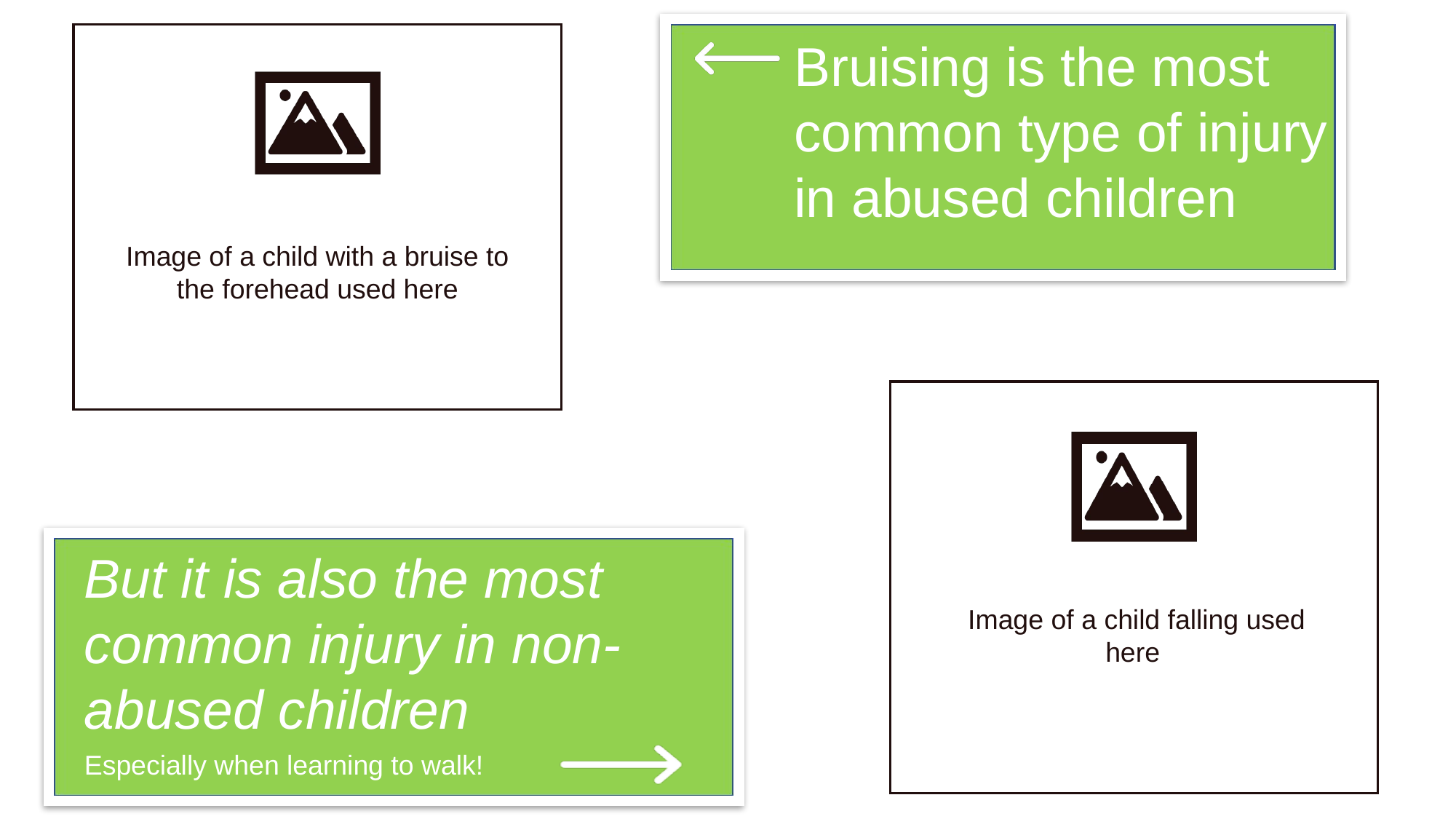

Bruising is the most common type of injury in abused children
Image of a child with a bruise to the forehead used here
Image of a child falling used here
But it is also the most common injury in non-abused children
Especially when learning to walk!

## Slide 25
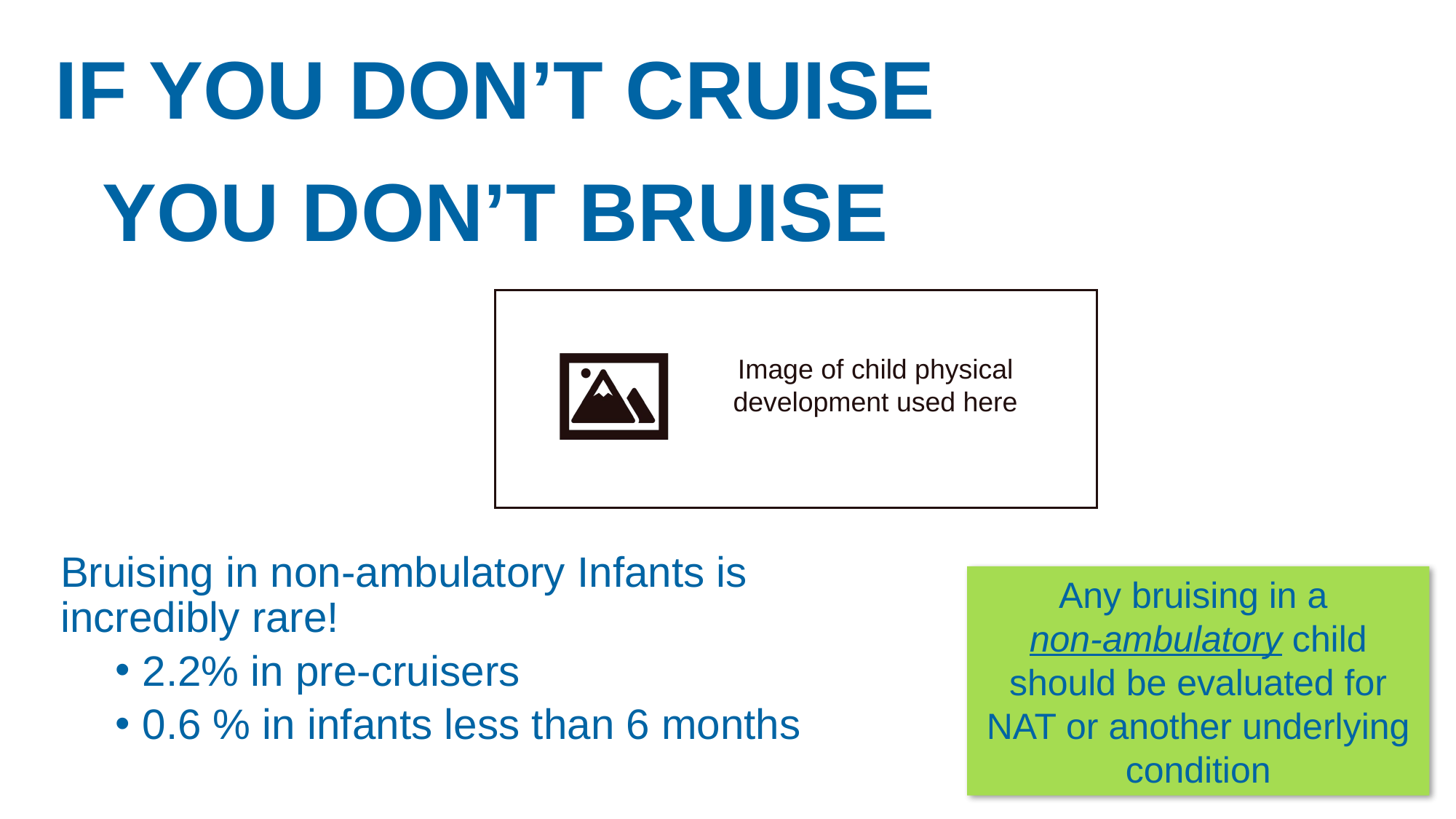

IF YOU DON’T CRUISE
YOU DON’T BRUISE
Image of child physical development used here
Bruising in non-ambulatory Infants is incredibly rare!
2.2% in pre-cruisers
0.6 % in infants less than 6 months
Any bruising in a
non-ambulatory child should be evaluated for NAT or another underlying condition

## Slide 26
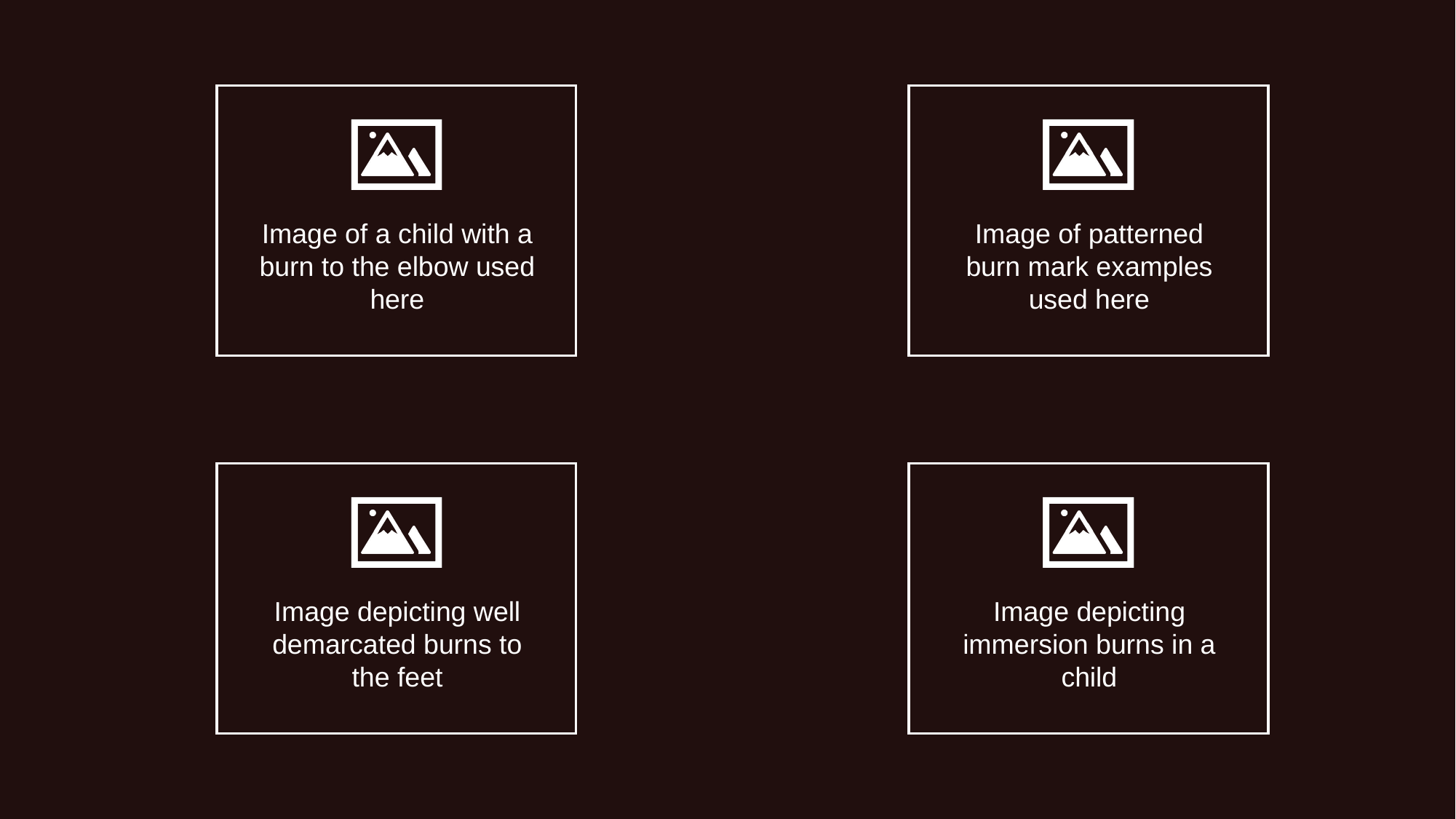

Image of patterned burn mark examples used here
Image of a child with a burn to the elbow used here
Image depicting well demarcated burns to the feet
Image depicting immersion burns in a child

## Slide 27
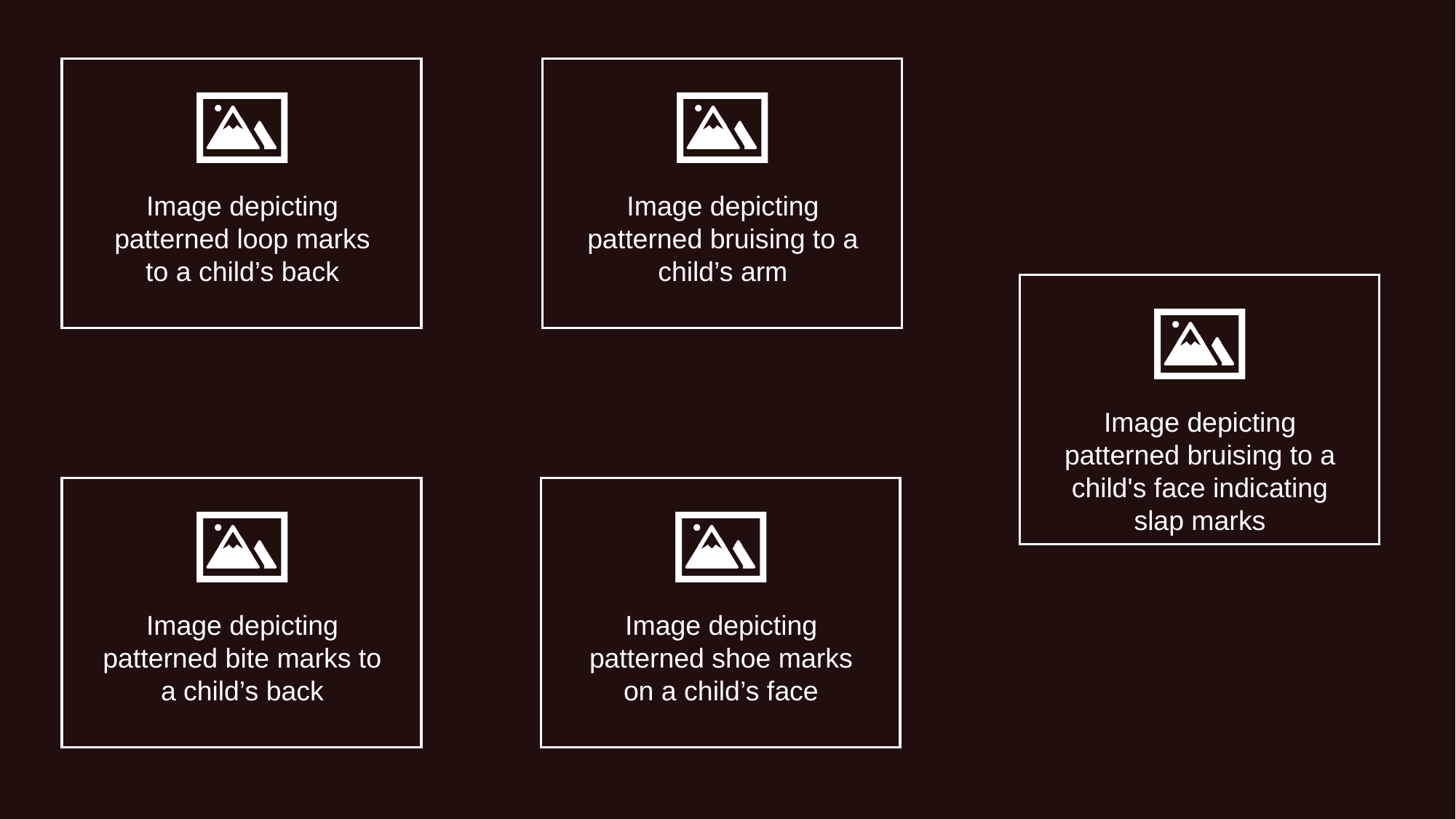

Image depicting patterned loop marks to a child’s back
Image depicting patterned bruising to a child’s arm
Image depicting patterned bruising to a child's face indicating slap marks
Image depicting patterned bite marks to a child’s back
Image depicting patterned shoe marks on a child’s face

## Slide 28
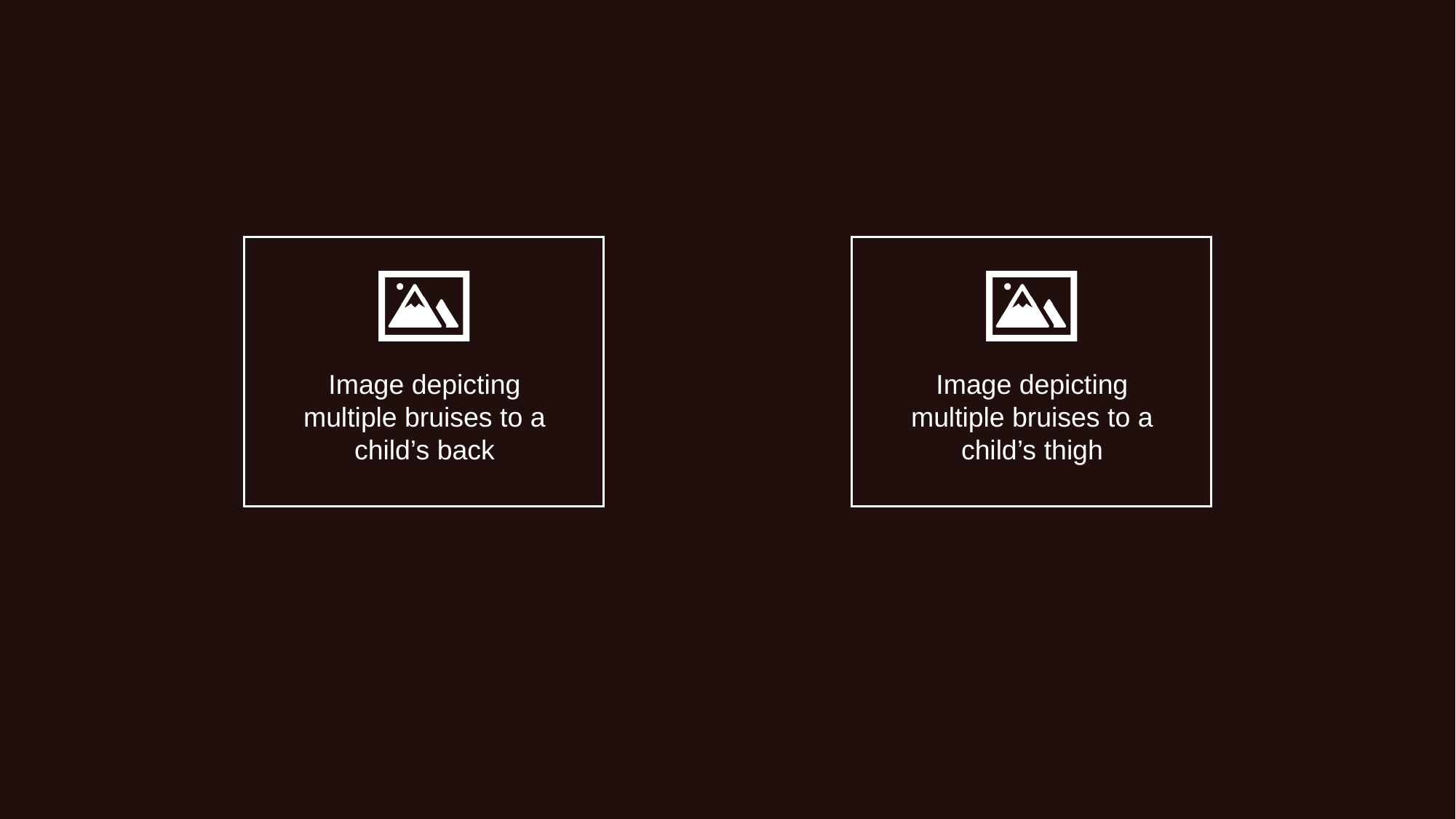

Image depicting multiple bruises to a child’s back
Image depicting multiple bruises to a child’s thigh

## Slide 29
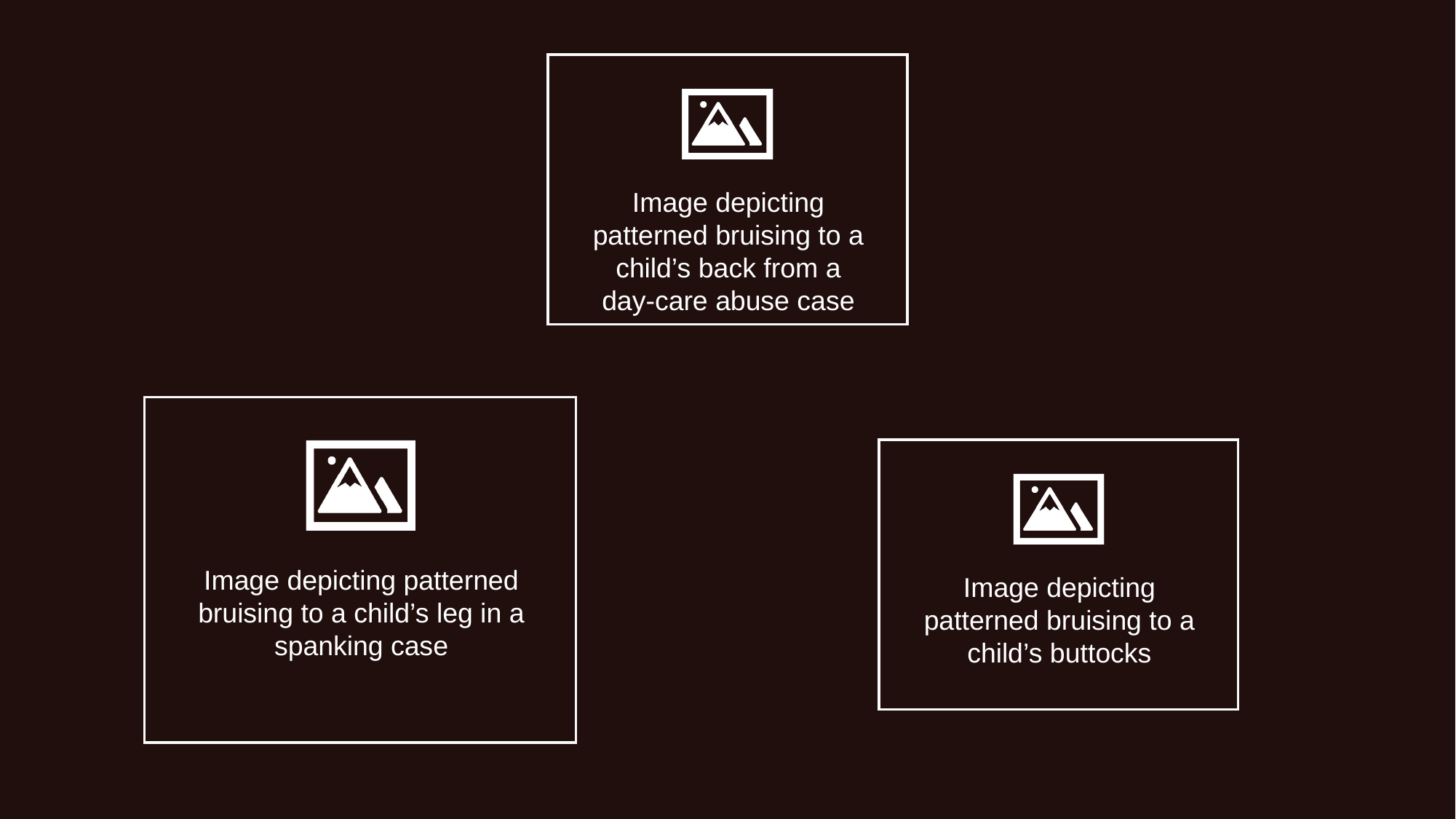

Image depicting patterned bruising to a child’s back from a day-care abuse case
Image depicting patterned bruising to a child’s leg in a spanking case
Image depicting patterned bruising to a child’s buttocks

## Slide 30
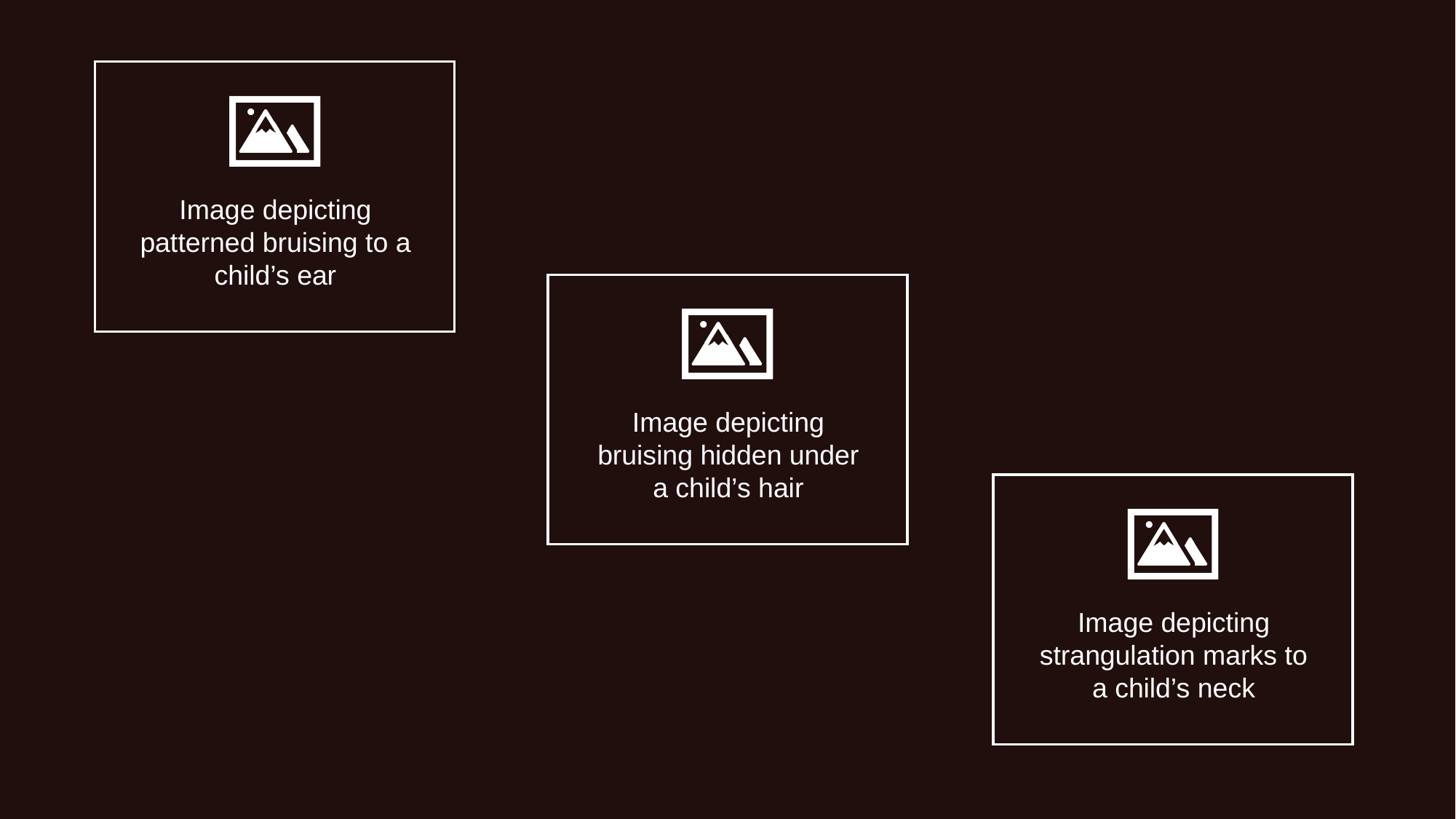

Image depicting patterned bruising to a child’s ear
Image depicting bruising hidden under a child’s hair
Image depicting strangulation marks to a child’s neck

## Slide 31
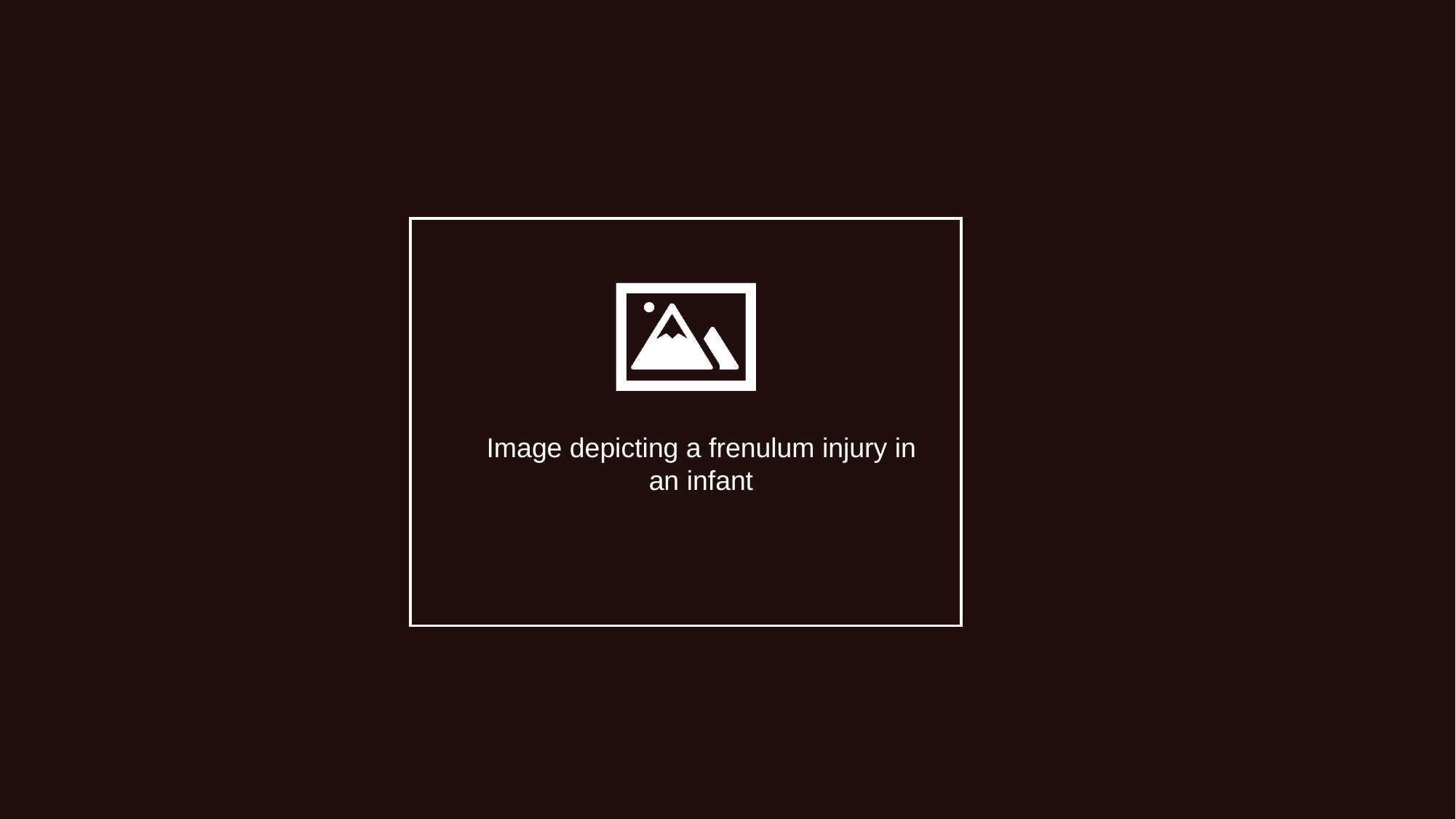

Image depicting a frenulum injury in an infant

## Slide 32
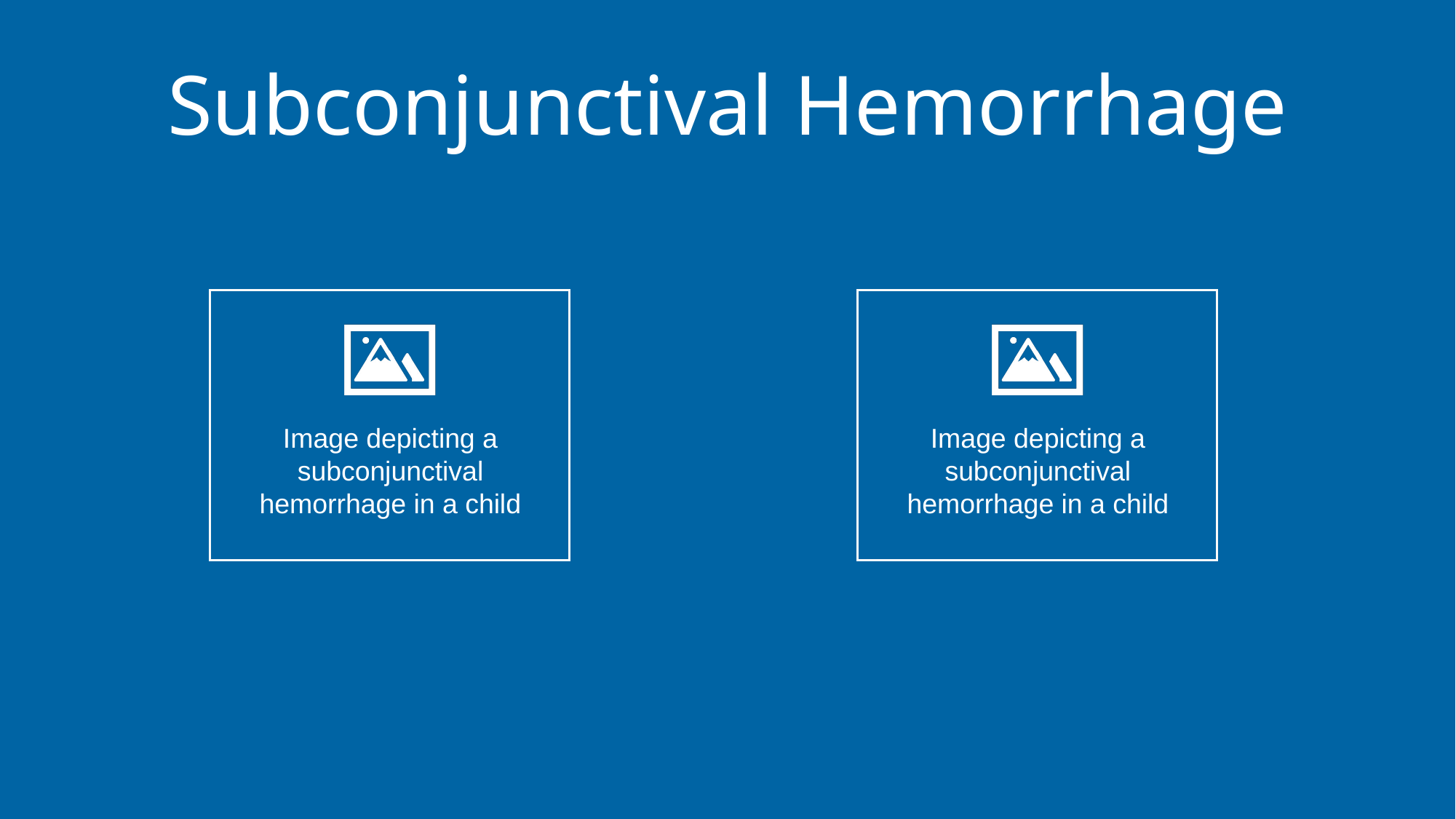

Subconjunctival Hemorrhage
Image depicting a subconjunctival hemorrhage in a child
Image depicting a subconjunctival hemorrhage in a child

## Slide 33
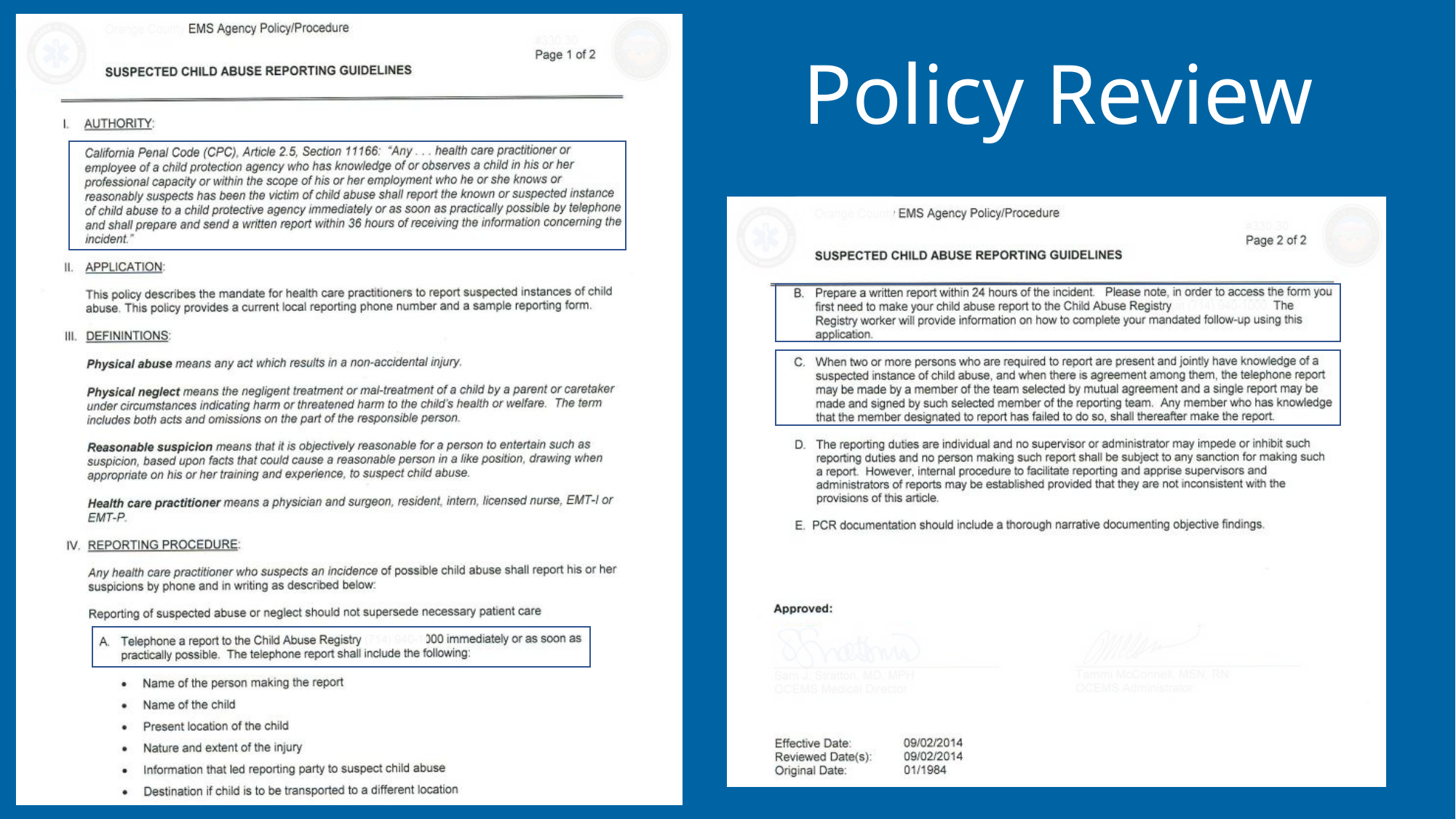

Policy Review

## Slide 34
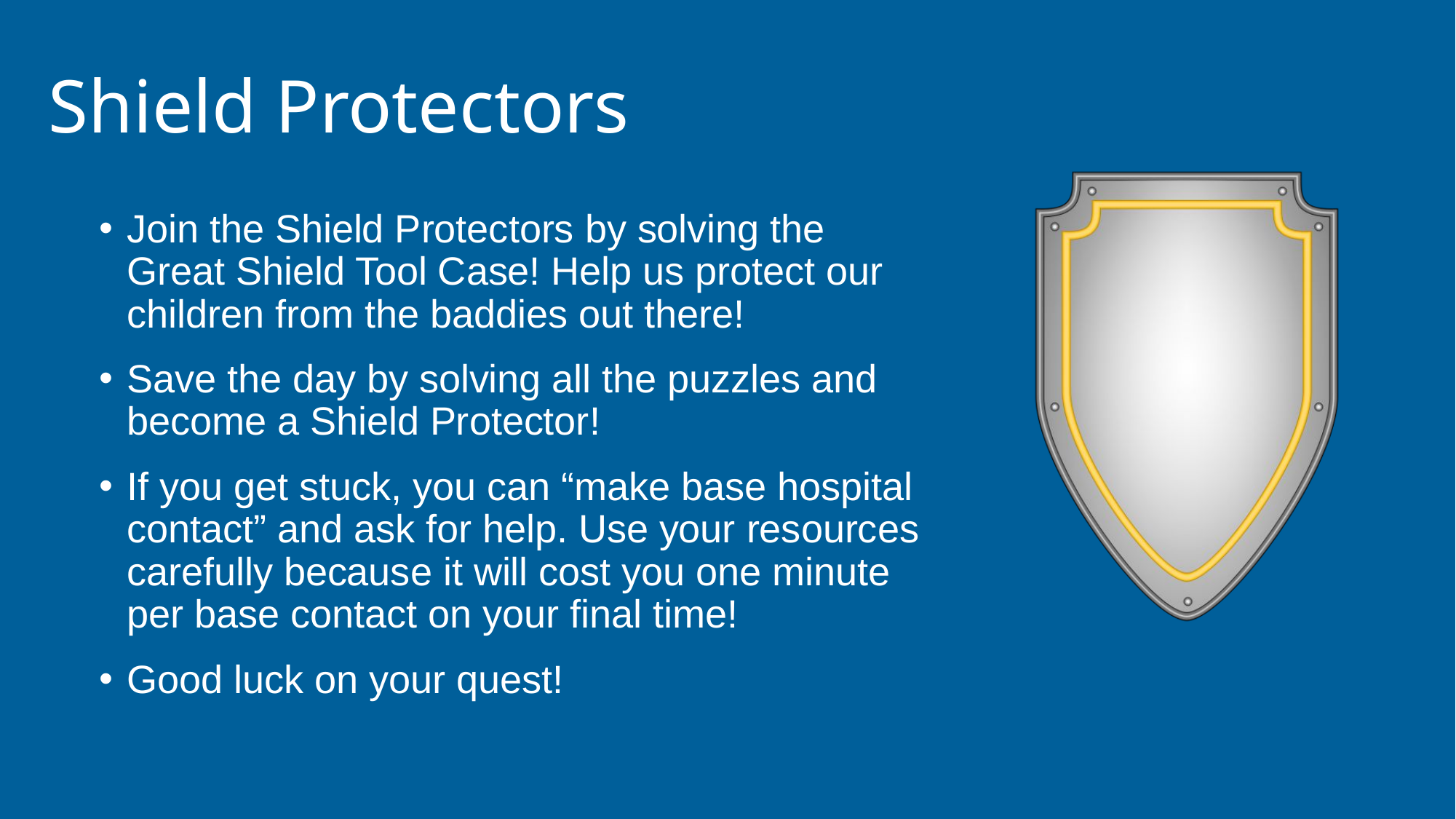

Shield Protectors
Join the Shield Protectors by solving the Great Shield Tool Case! Help us protect our children from the baddies out there!
Save the day by solving all the puzzles and become a Shield Protector!
If you get stuck, you can “make base hospital contact” and ask for help. Use your resources carefully because it will cost you one minute per base contact on your final time!
Good luck on your quest!
