# Supplementary material for: Child Maltreatment Education: Utilizing an Escape Room Activity to Engage Learners on a Sensitive Topic
Source: J Educ Teach Emerg Med. 2023 Jan 31;8(1):SG1–SG21. doi: 10.21980/J84H1C (PMC10332768; doi:10.21980/J84H1C)
Supplement: Supplementary file 2 [file jetem-8-1-sg1-appendixB1.pptx]

## Slide 1
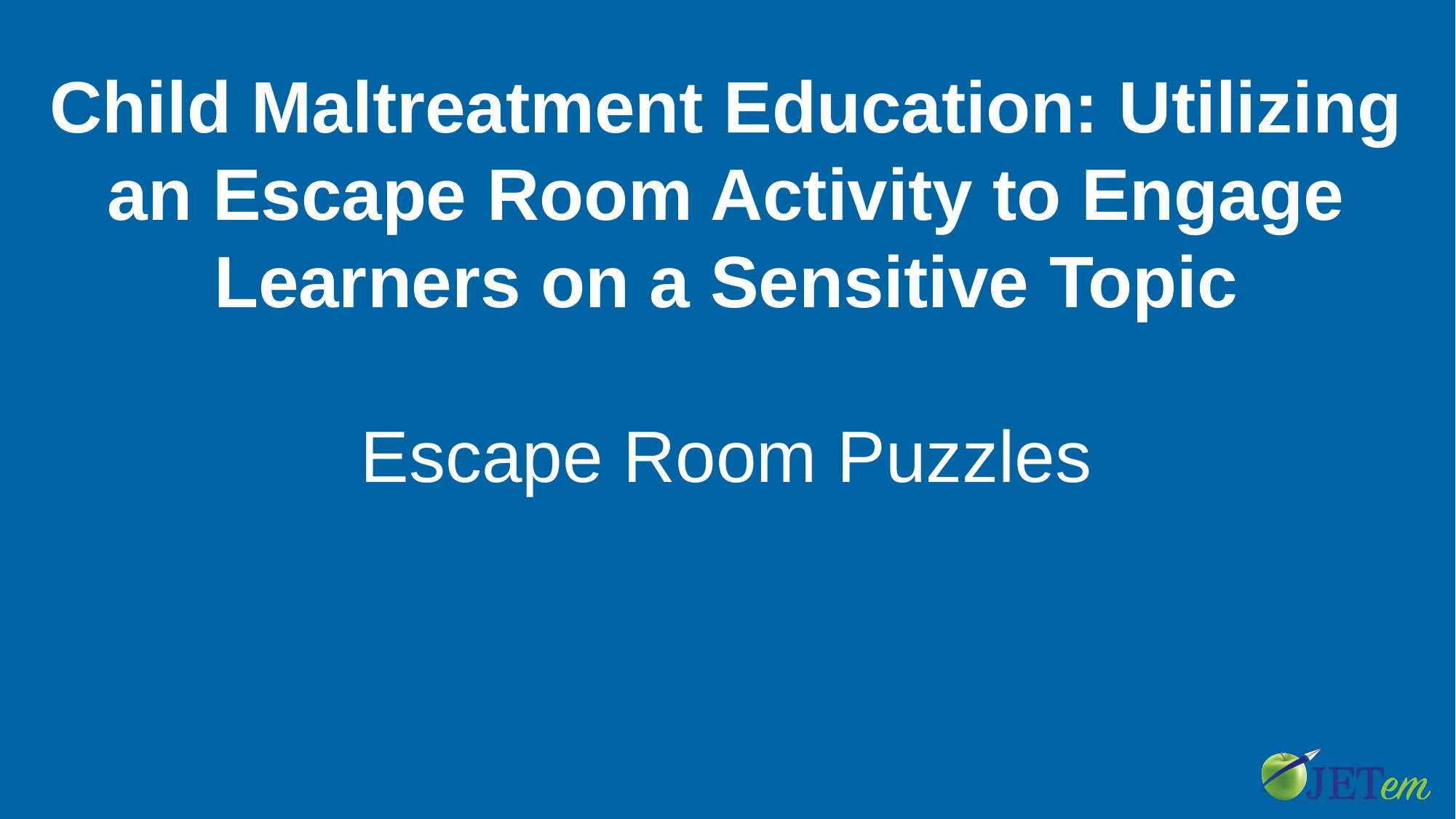

Child Maltreatment Education: Utilizing an Escape Room Activity to Engage Learners on a Sensitive Topic
Escape Room Puzzles

## Slide 2
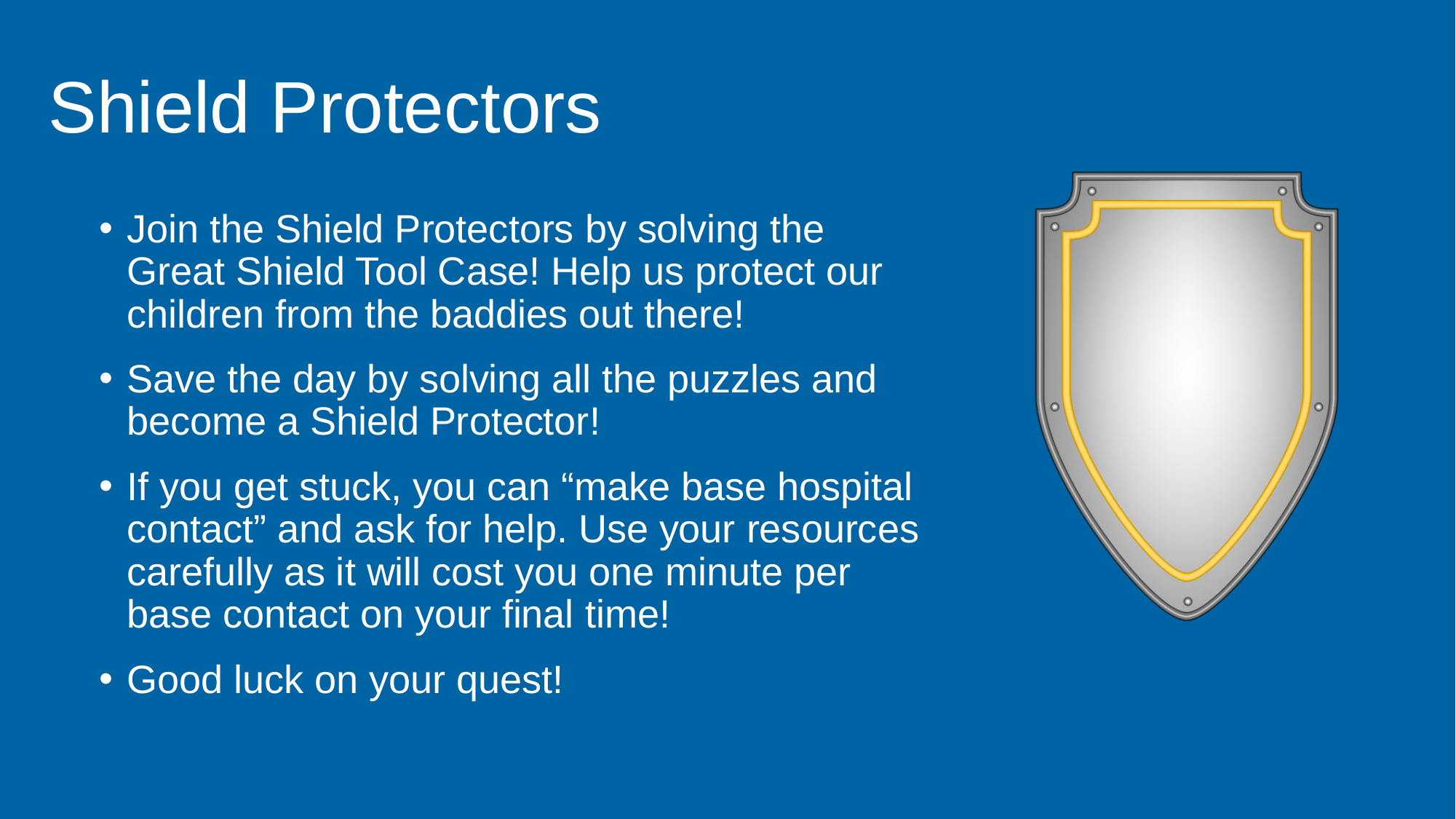

Shield Protectors
Join the Shield Protectors by solving the Great Shield Tool Case! Help us protect our children from the baddies out there!
Save the day by solving all the puzzles and become a Shield Protector!
If you get stuck, you can “make base hospital contact” and ask for help. Use your resources carefully as it will cost you one minute per base contact on your final time!
Good luck on your quest!

## Slide 3
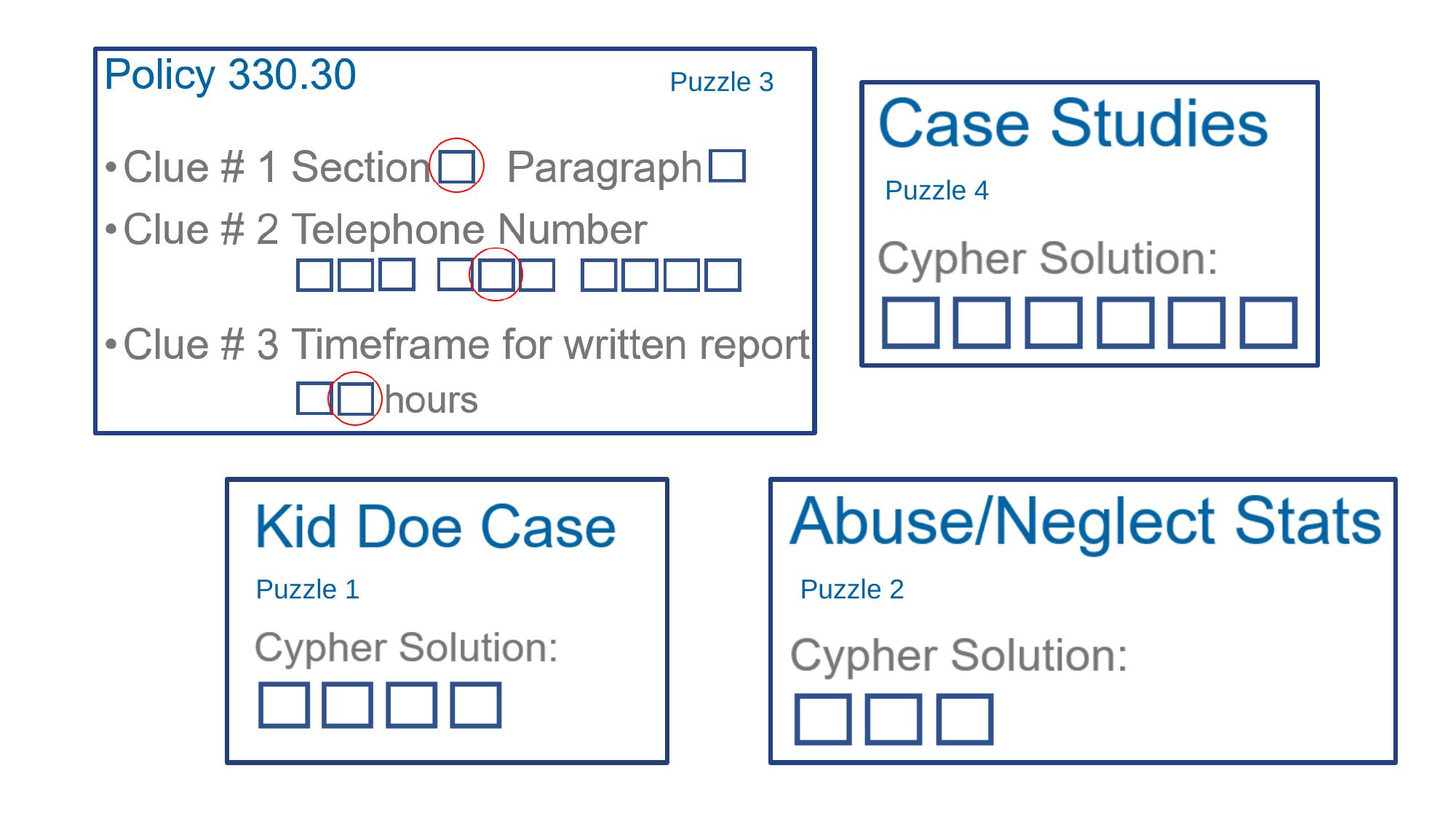

Puzzle 3
Puzzle 4
Puzzle 1
Puzzle 2

## Slide 4
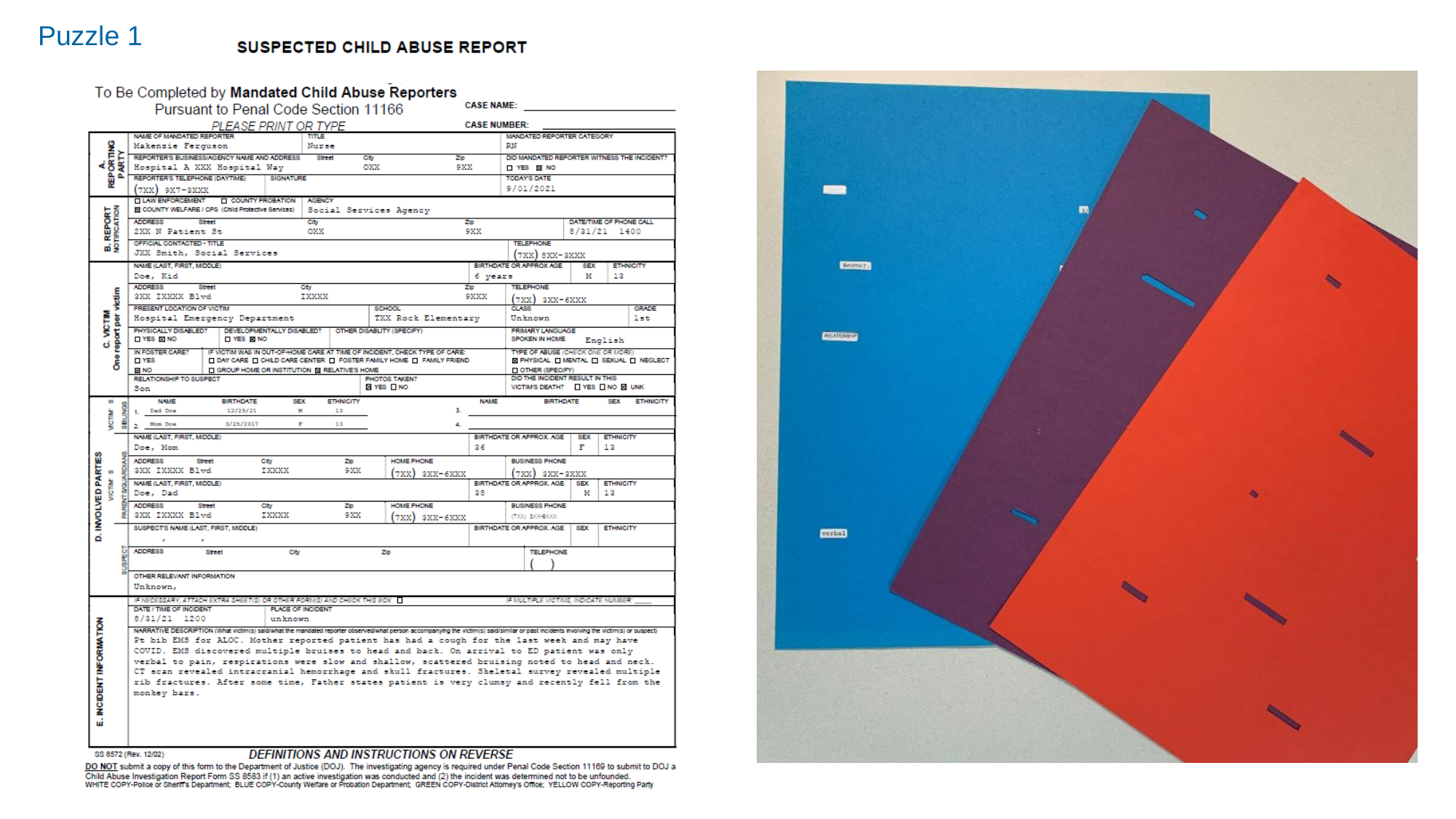

Puzzle 1

## Slide 5
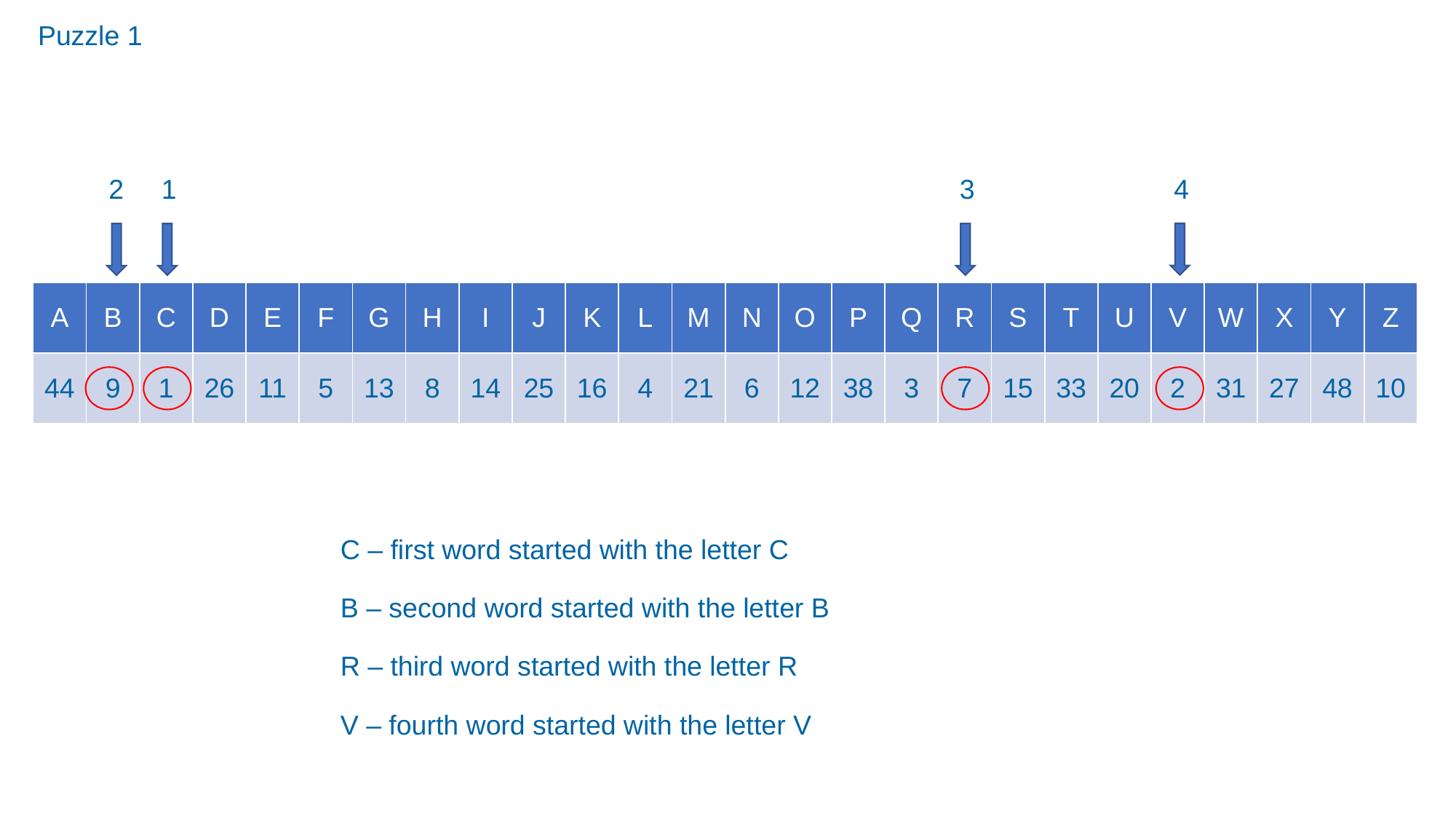

Puzzle 1
2
1
3
4
| A | B | C | D | E | F | G | H | I | J | K | L | M | N | O | P | Q | R | S | T | U | V | W | X | Y | Z |
| --- | --- | --- | --- | --- | --- | --- | --- | --- | --- | --- | --- | --- | --- | --- | --- | --- | --- | --- | --- | --- | --- | --- | --- | --- | --- |
| 44 | 9 | 1 | 26 | 11 | 5 | 13 | 8 | 14 | 25 | 16 | 4 | 21 | 6 | 12 | 38 | 3 | 7 | 15 | 33 | 20 | 2 | 31 | 27 | 48 | 10 |
C – first word started with the letter C
B – second word started with the letter B
R – third word started with the letter R
V – fourth word started with the letter V

## Slide 6
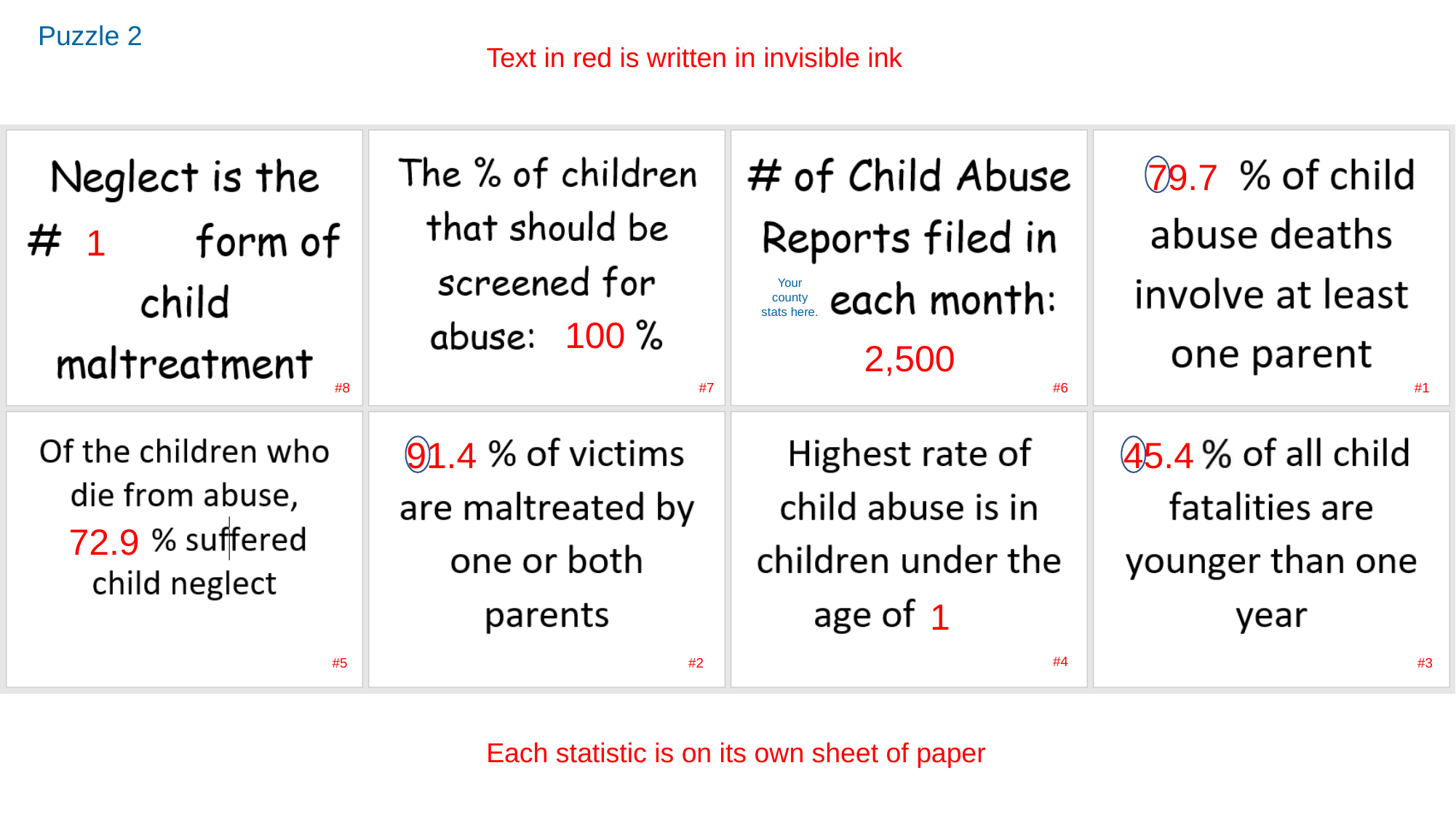

Puzzle 2
Text in red is written in invisible ink
79.7
1
Your county stats here.
100
2,500
#8
#7
#6
#1
45.4
91.4
72.9
1
#4
#5
#2
#3
Each statistic is on its own sheet of paper

## Slide 7
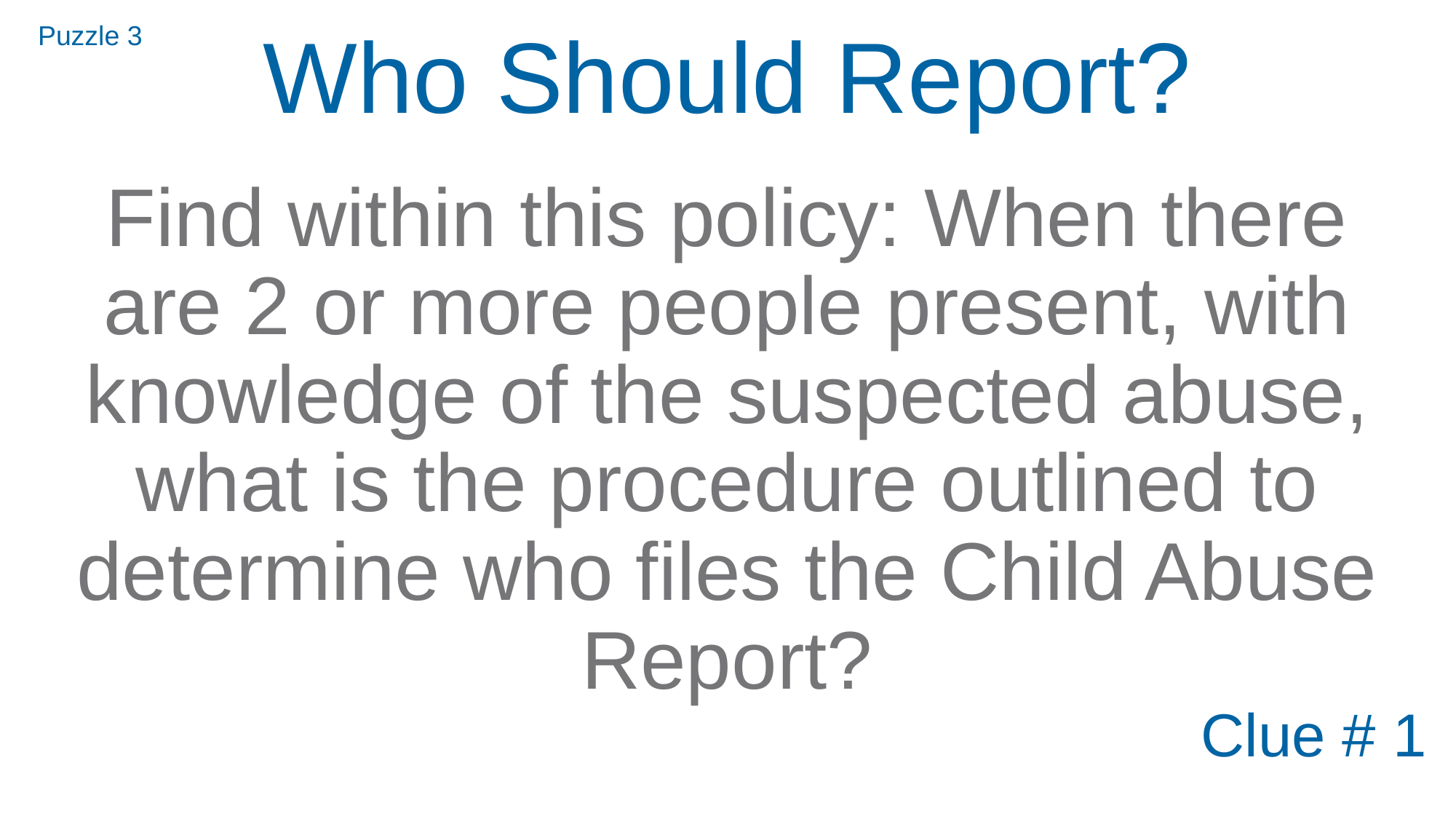

# Who Should Report?
Puzzle 3
Find within this policy: When there are 2 or more people present, with knowledge of the suspected abuse, what is the procedure outlined to determine who files the Child Abuse Report?
Clue # 1

## Slide 8
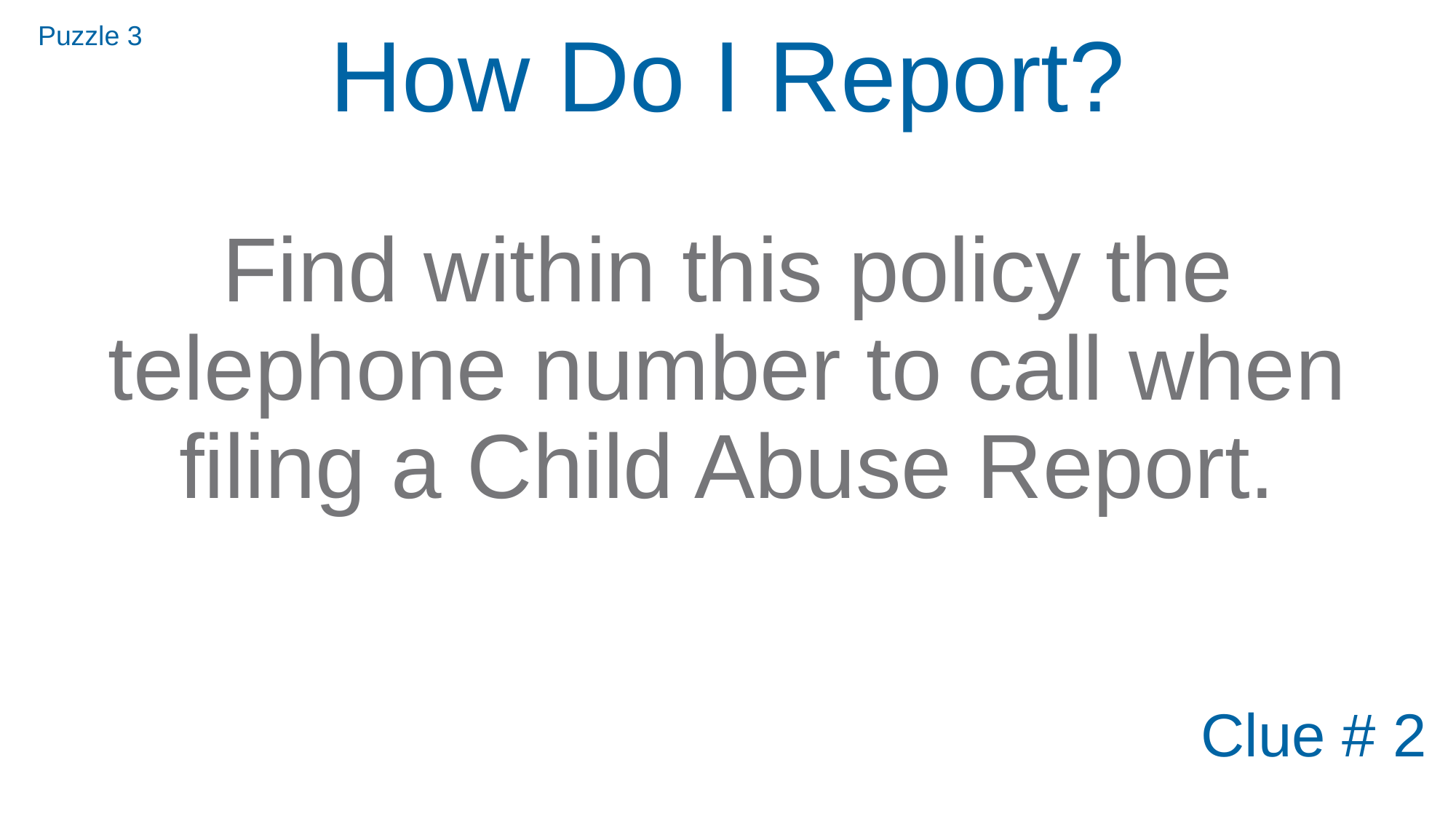

# How Do I Report?
Puzzle 3
Find within this policy the telephone number to call when filing a Child Abuse Report.
Clue # 2

## Slide 9
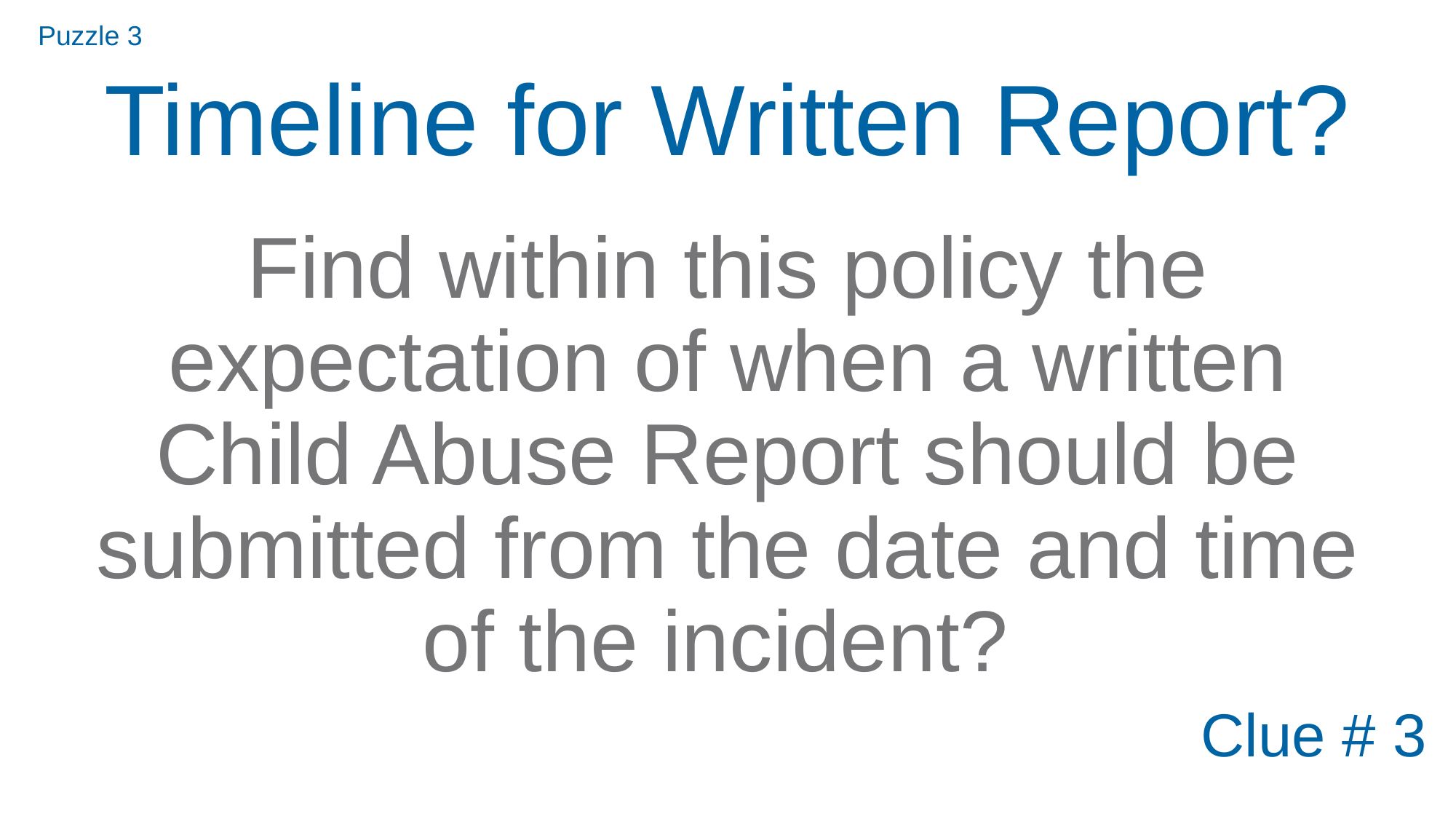

Puzzle 3
# Timeline for Written Report?
Find within this policy the expectation of when a written Child Abuse Report should be submitted from the date and time of the incident?
Clue # 3

## Slide 10
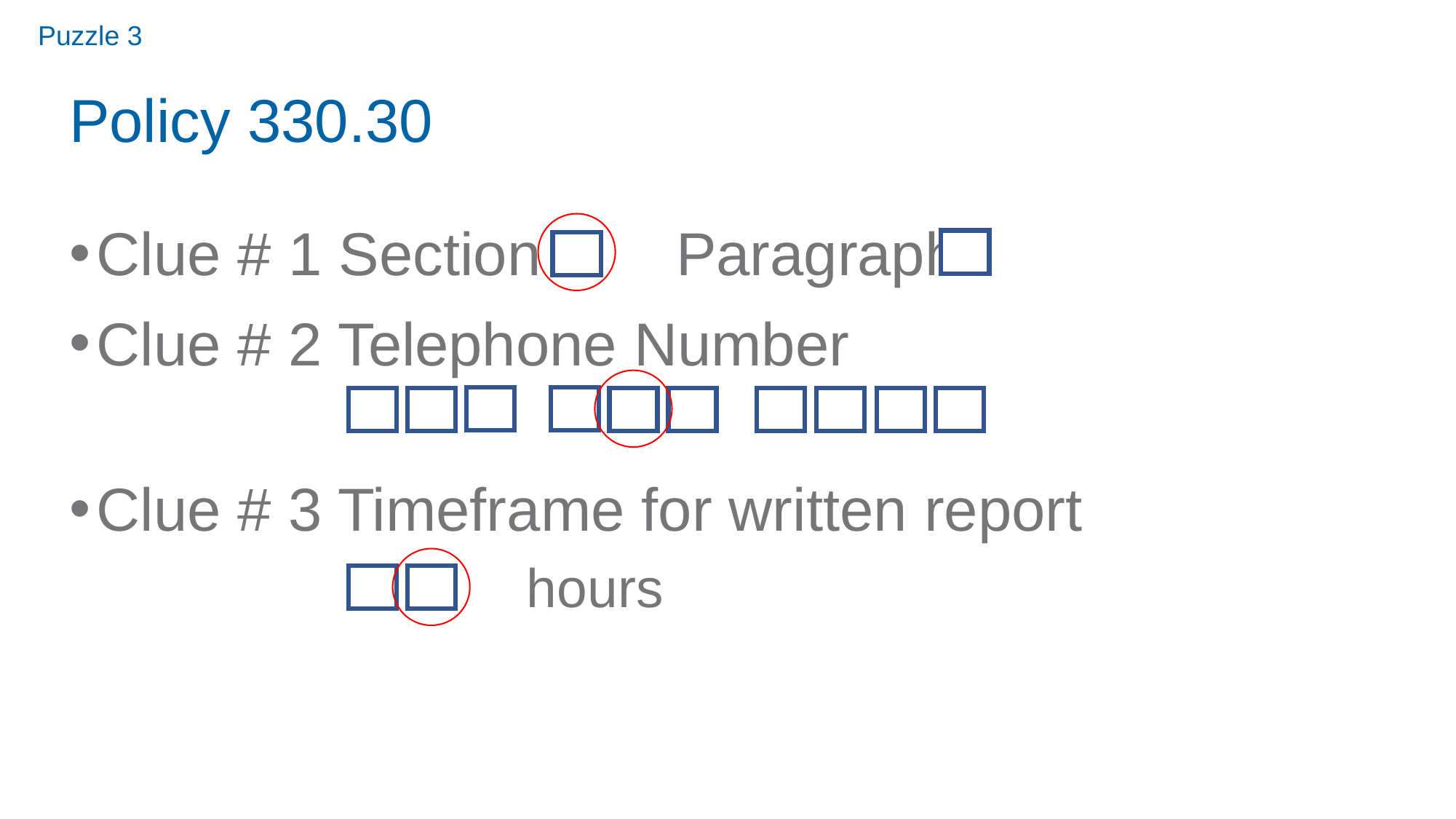

Puzzle 3
# Policy 330.30
Clue # 1 Section 	 Paragraph
Clue # 2 Telephone Number
Clue # 3 Timeframe for written report
			 hours

## Slide 11
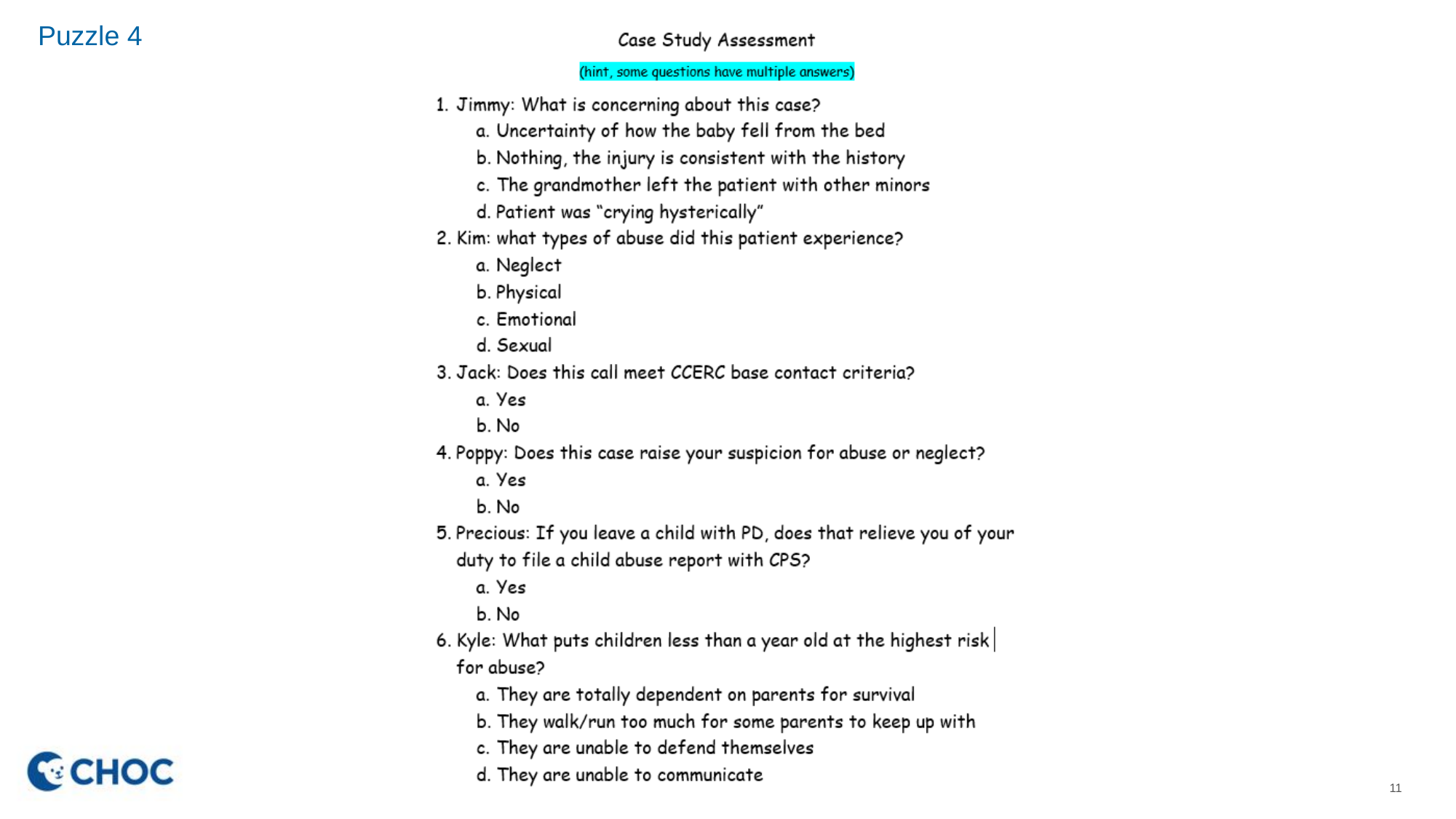

Puzzle 4

## Slide 12
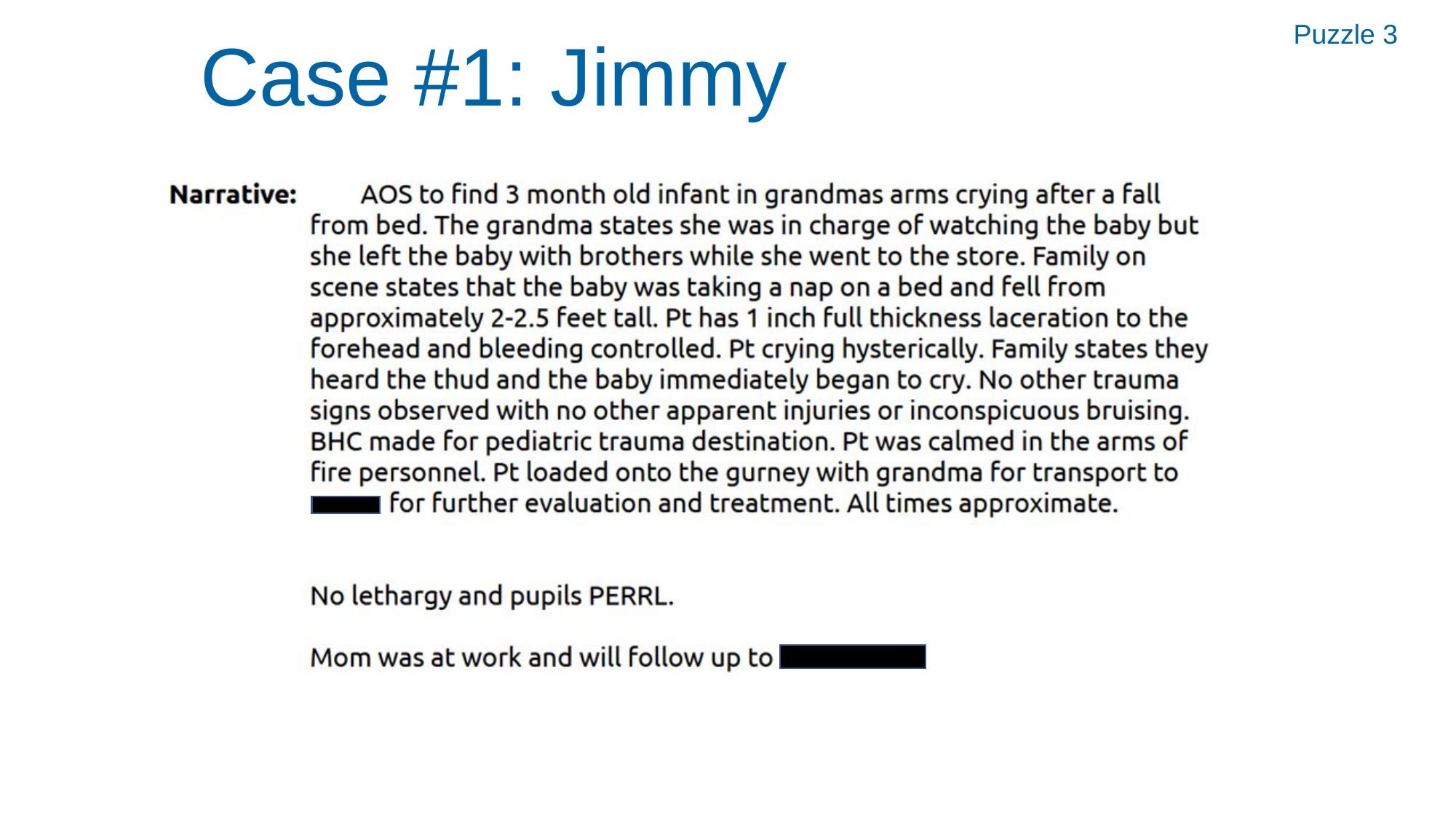

Puzzle 3
Case #1: Jimmy

## Slide 13
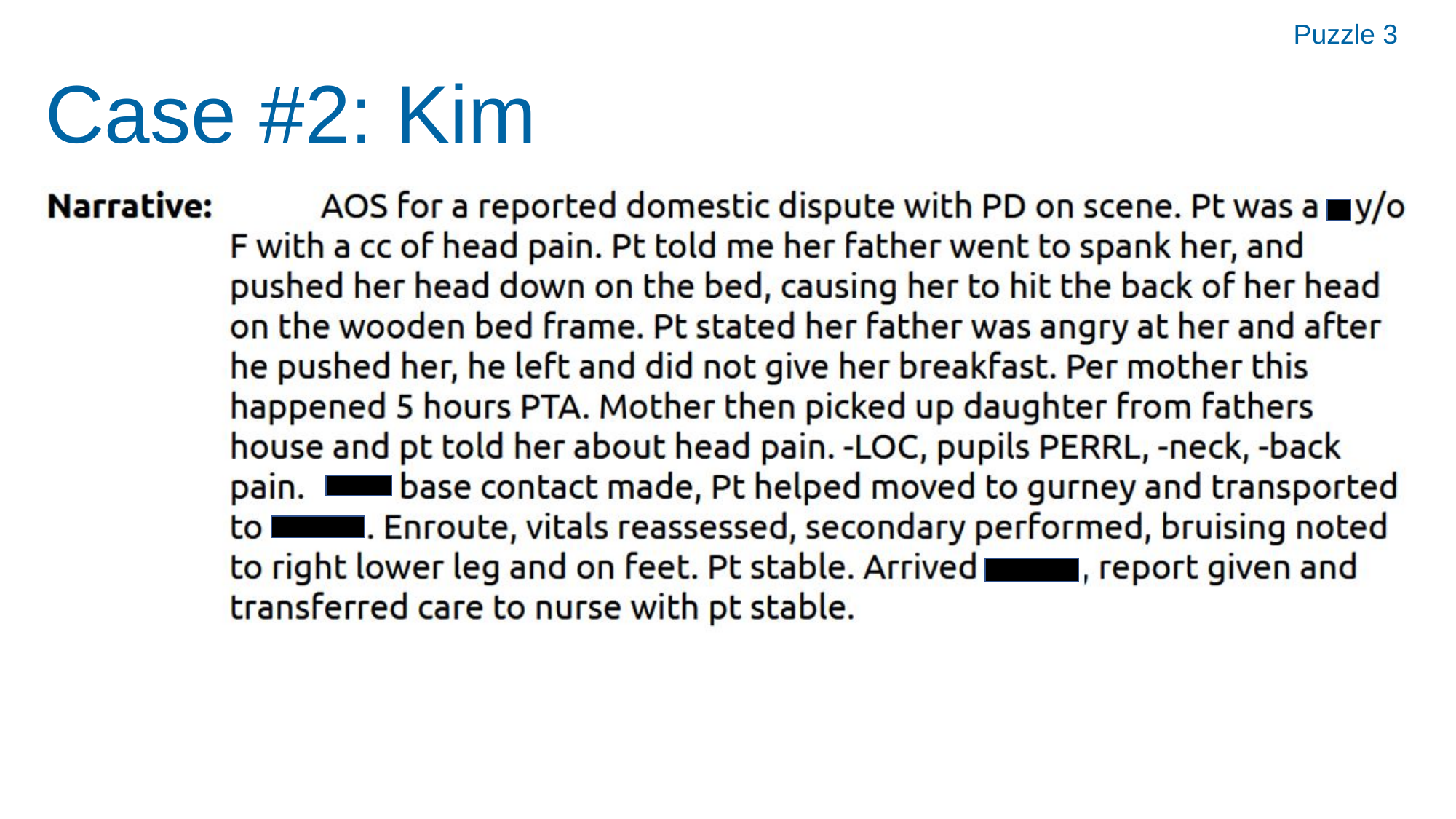

Puzzle 3
Case #2: Kim

## Slide 14
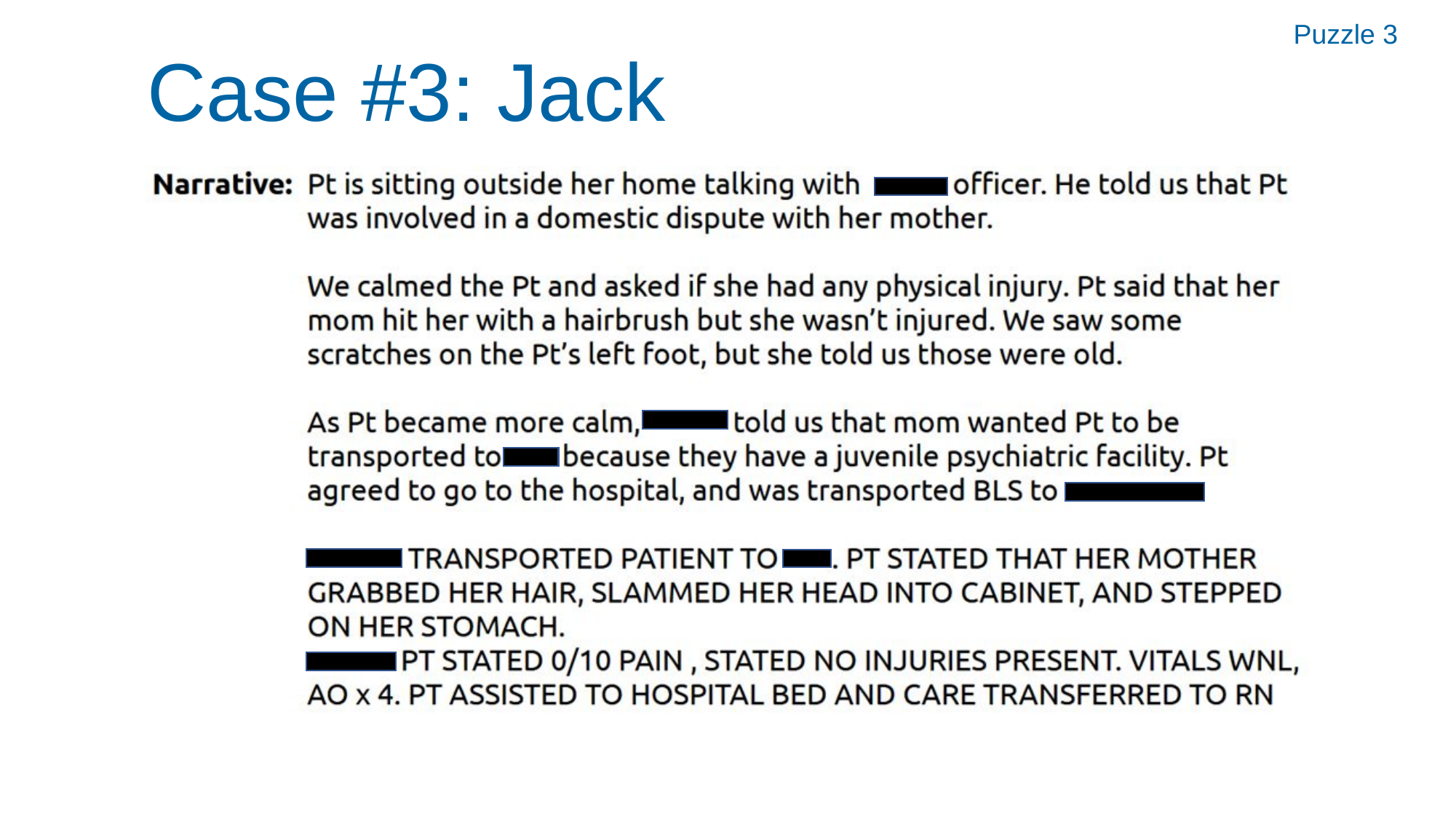

Puzzle 3
Case #3: Jack

## Slide 15
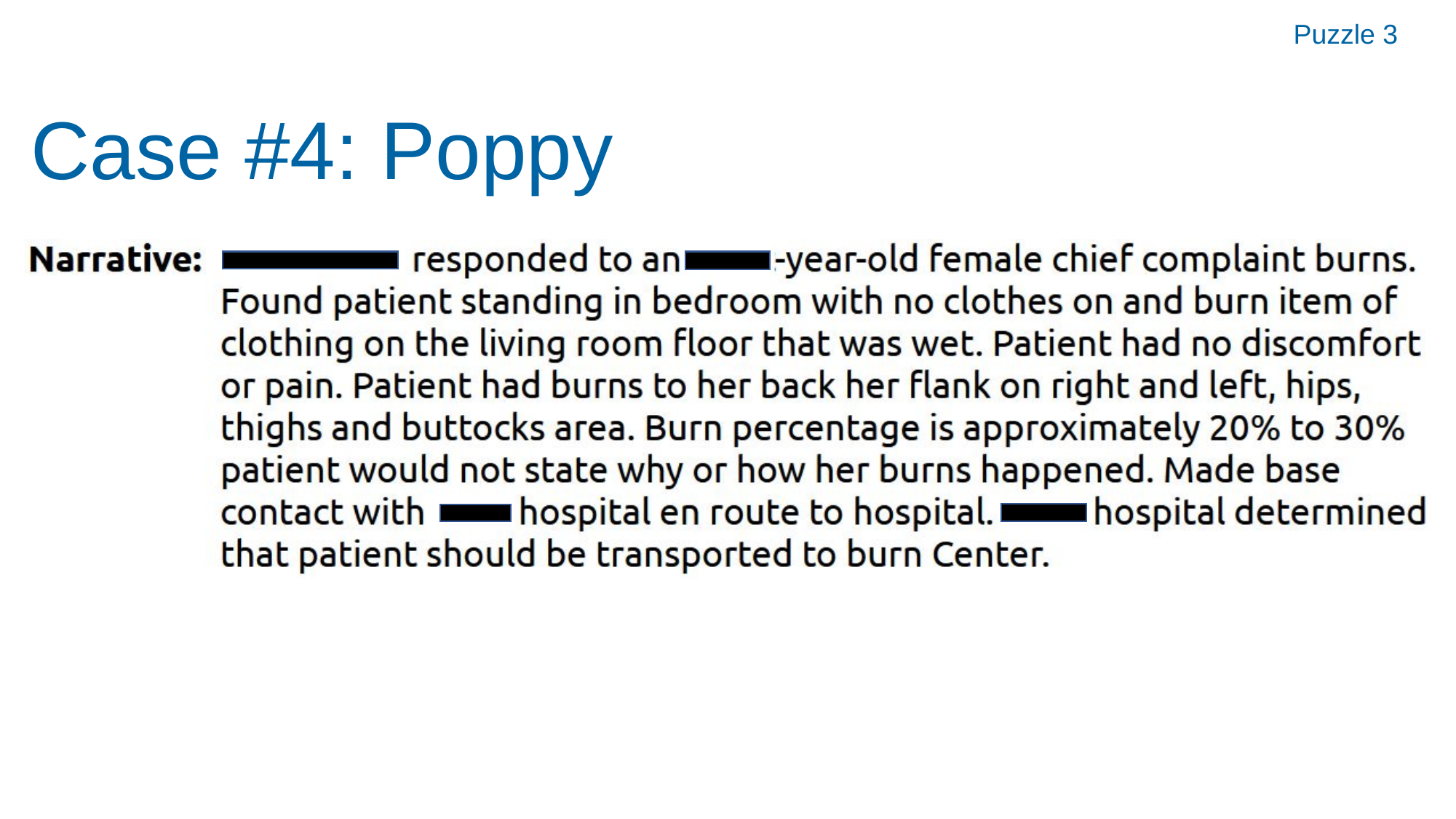

Puzzle 3
Case #4: Poppy

## Slide 16
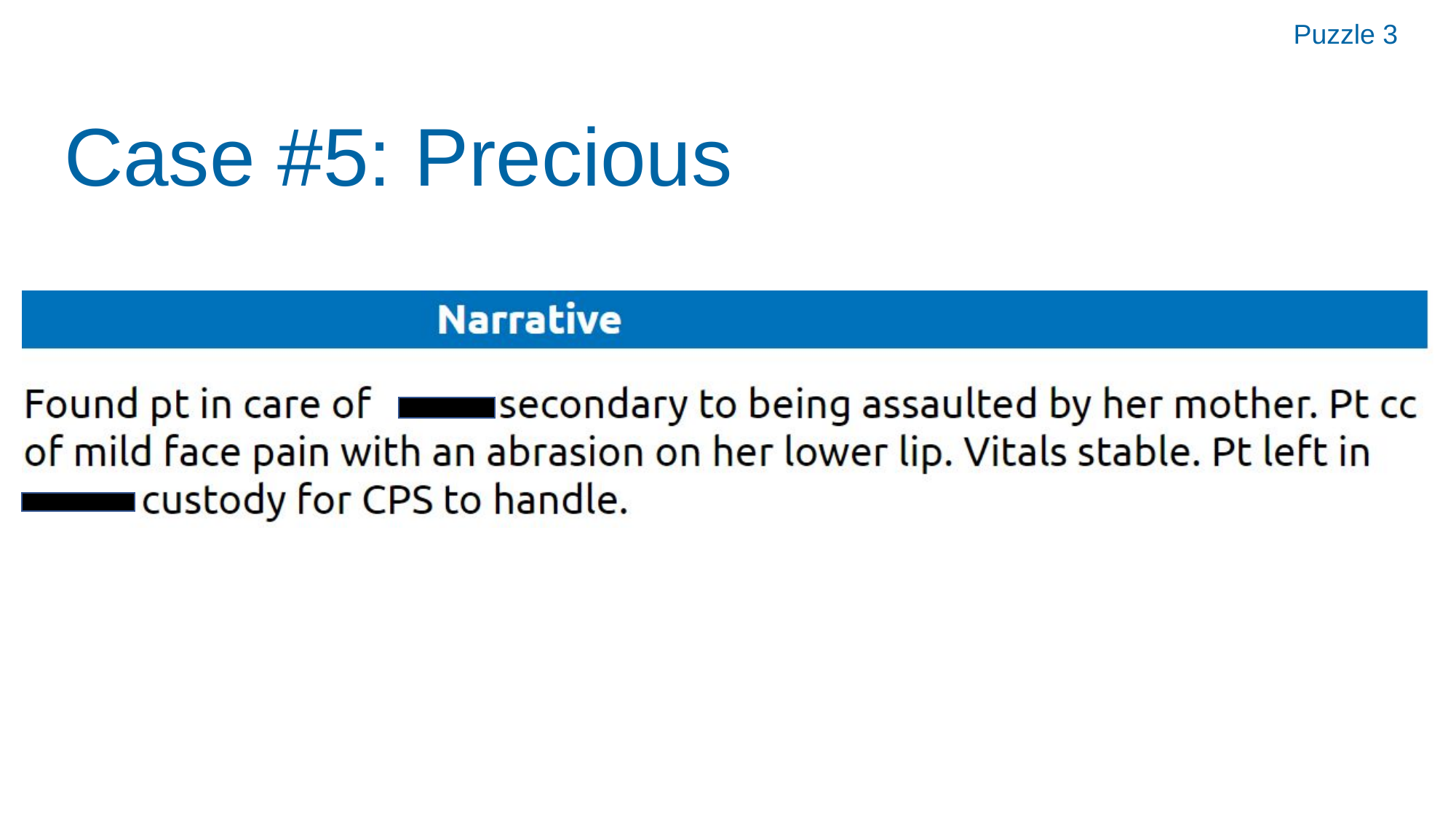

Puzzle 3
Case #5: Precious

## Slide 17
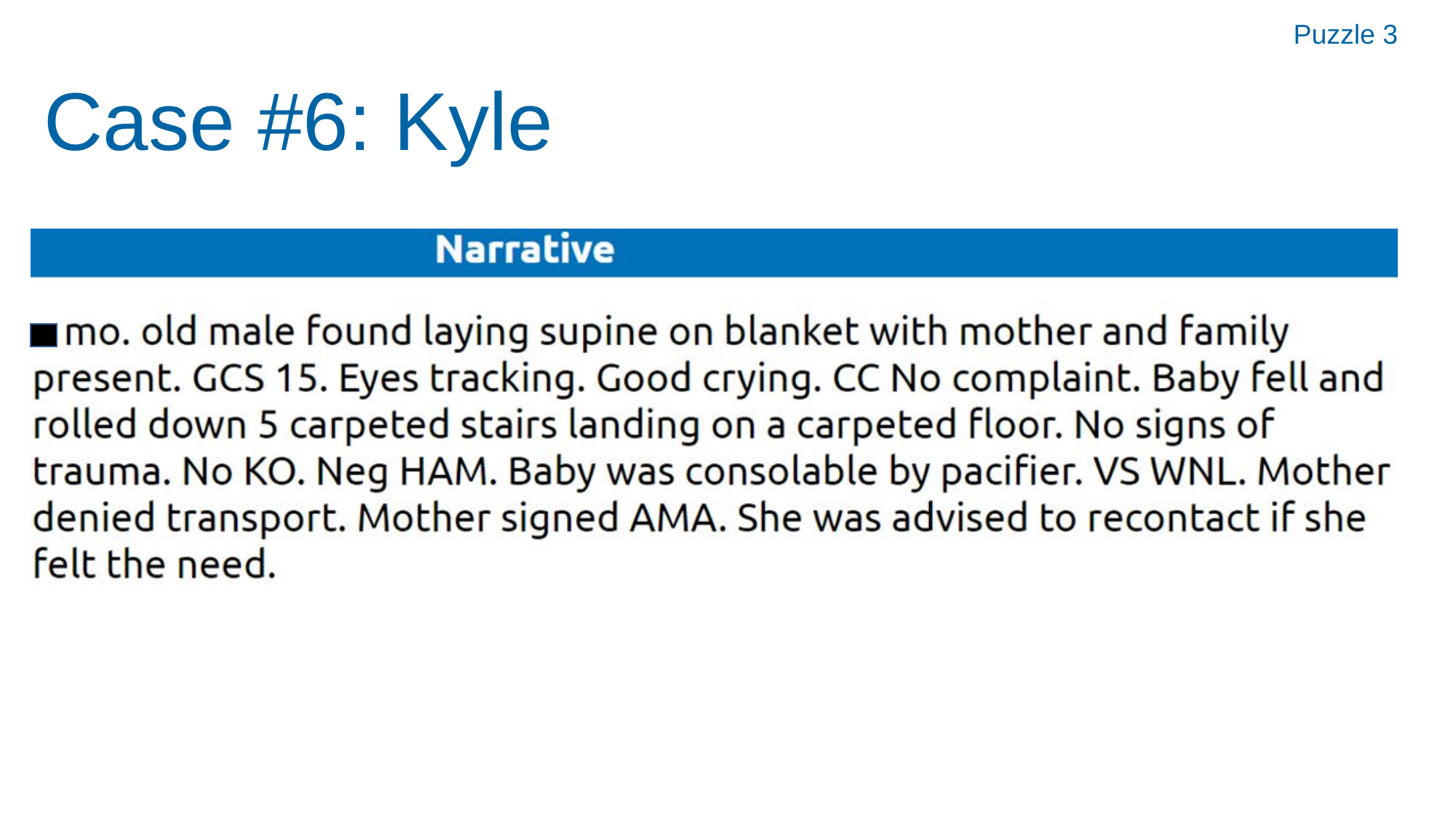

Puzzle 3
Case #6: Kyle

## Slide 18
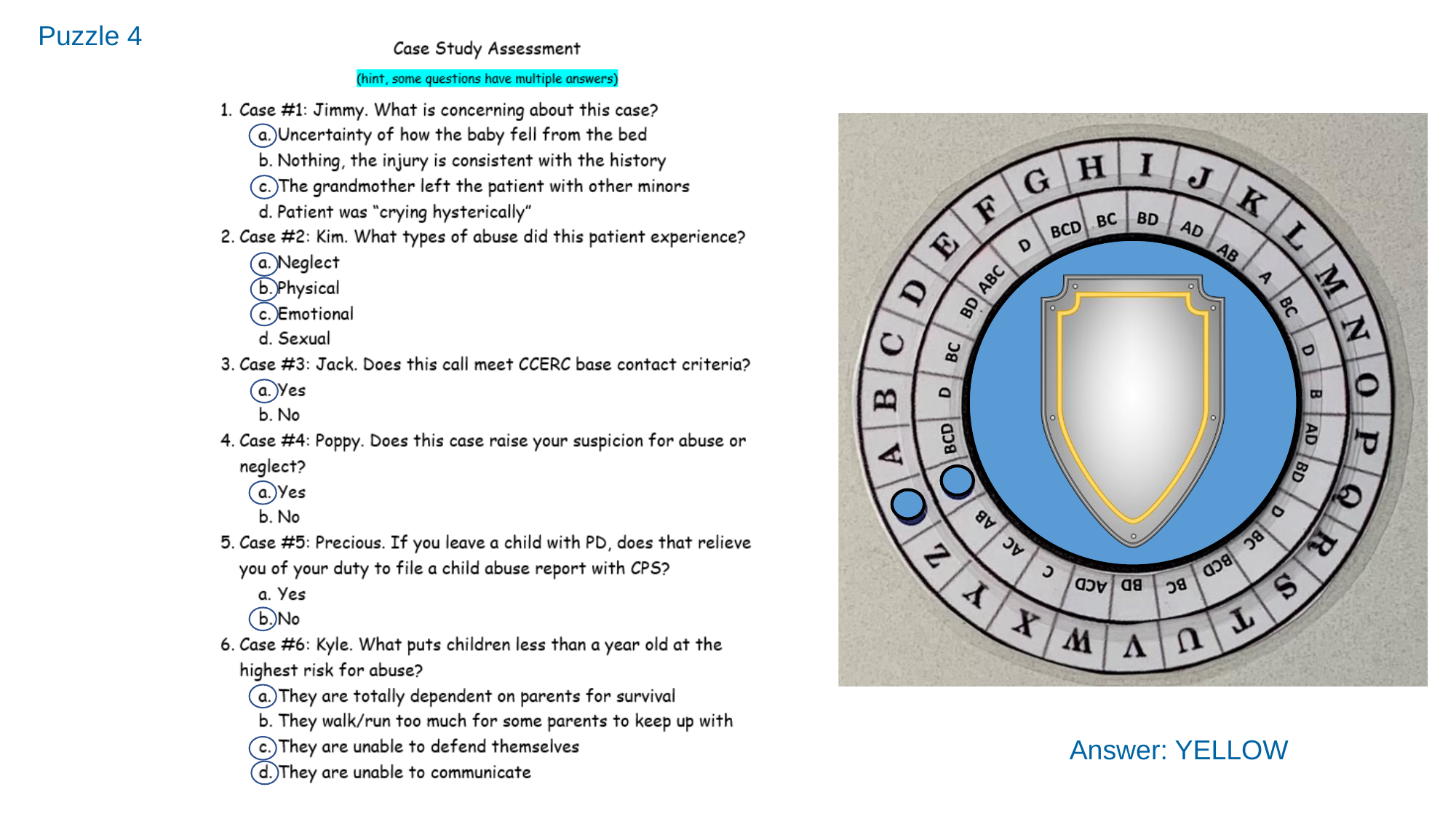

Puzzle 4
Answer: YELLOW

## Slide 19
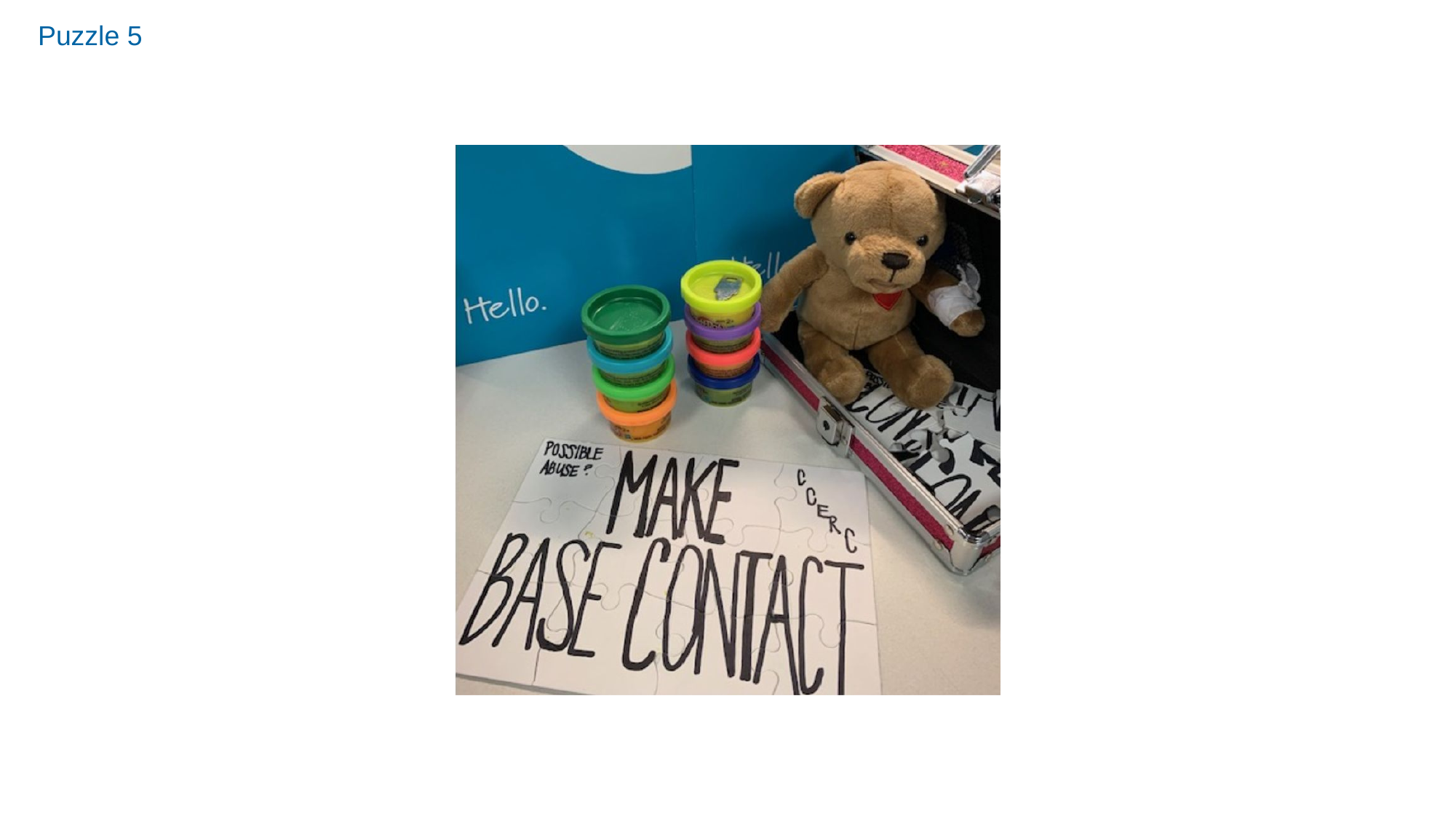

Puzzle 5
