# Supplementary material for: Child Maltreatment Education: Utilizing an Escape Room Activity to Engage Learners on a Sensitive Topic
Source: J Educ Teach Emerg Med. 2023 Jan 31;8(1):SG1–SG21. doi: 10.21980/J84H1C (PMC10332768; doi:10.21980/J84H1C)
Supplement: Supplementary file 3 [file jetem-8-1-sg1-appendixB2.docx]

Appendix B:

Instructions for the Escape Room


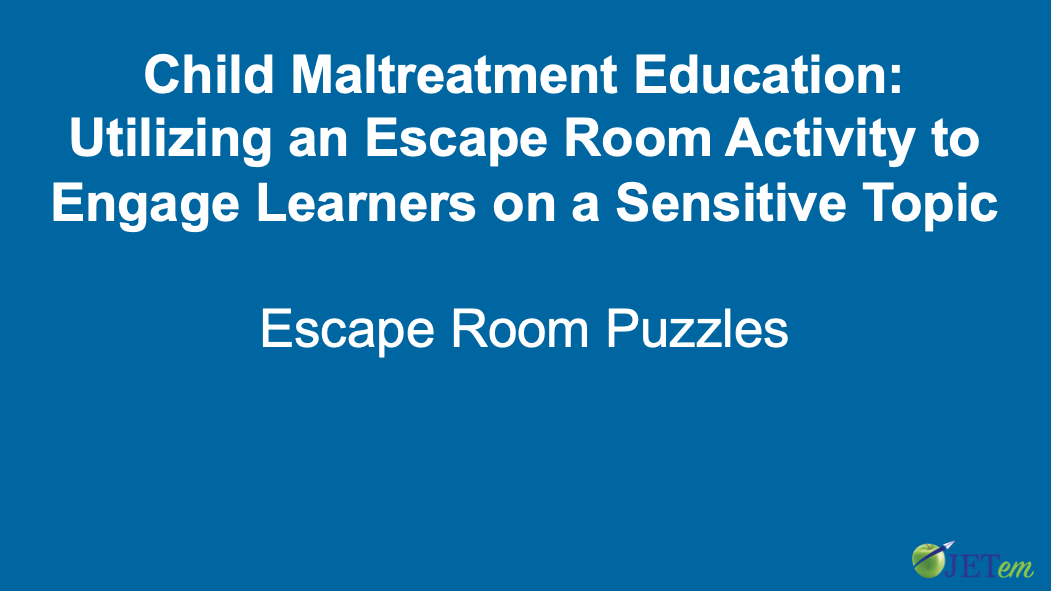


Please see associated PowerPoint file

**Instructions:**

Refer to “Escape Room Puzzles” PowerPoint. Adjust the slides for your institution. We recommend laminating the paper puzzles. This protects the sheets and allows the learners to write on the clues.

**Stations:**

There are 5 puzzles the learners must solve.

**Puzzle 1: Kid Doe Suspected Child Abuse Report**

**Materials:** You will need the fake Kid Doe Suspected Child Abuse Report and three different colors of card stock paper. Each sheet will have different windows cut out. In our room we used the blue card to find the 4 words we cut out. There are distractors cut out on the blue card, but they are numbers only. The two other colored sheets don’t reveal full words on the report. The learners will use the first letter of each word to find the 4-digit number lock combination. Refer to slides 3-4 in the Escape Room Puzzles PowerPoint.

**Instructions:** Puzzle number one is to decode a fictional Suspected Child Abuse Report Form.

1. The learner must identify the correct color cut-out card.
2. Use that card to find the correct four words in the report.
3. The first letter of each word corresponds with a number on the table cypher which unlocks the box containing the UV light.


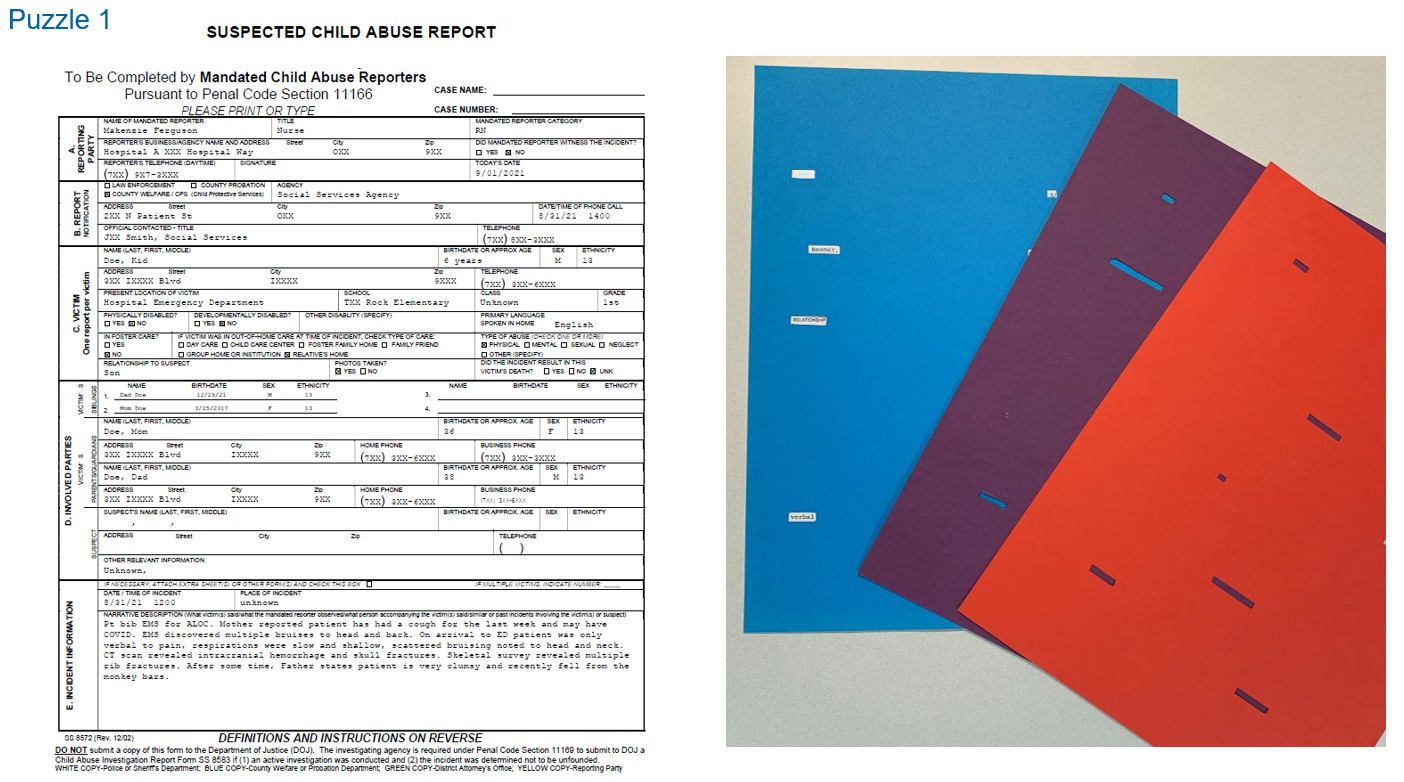


Puzzle number 1: Fictional Suspected Child Abuse Report with the color card decoders.


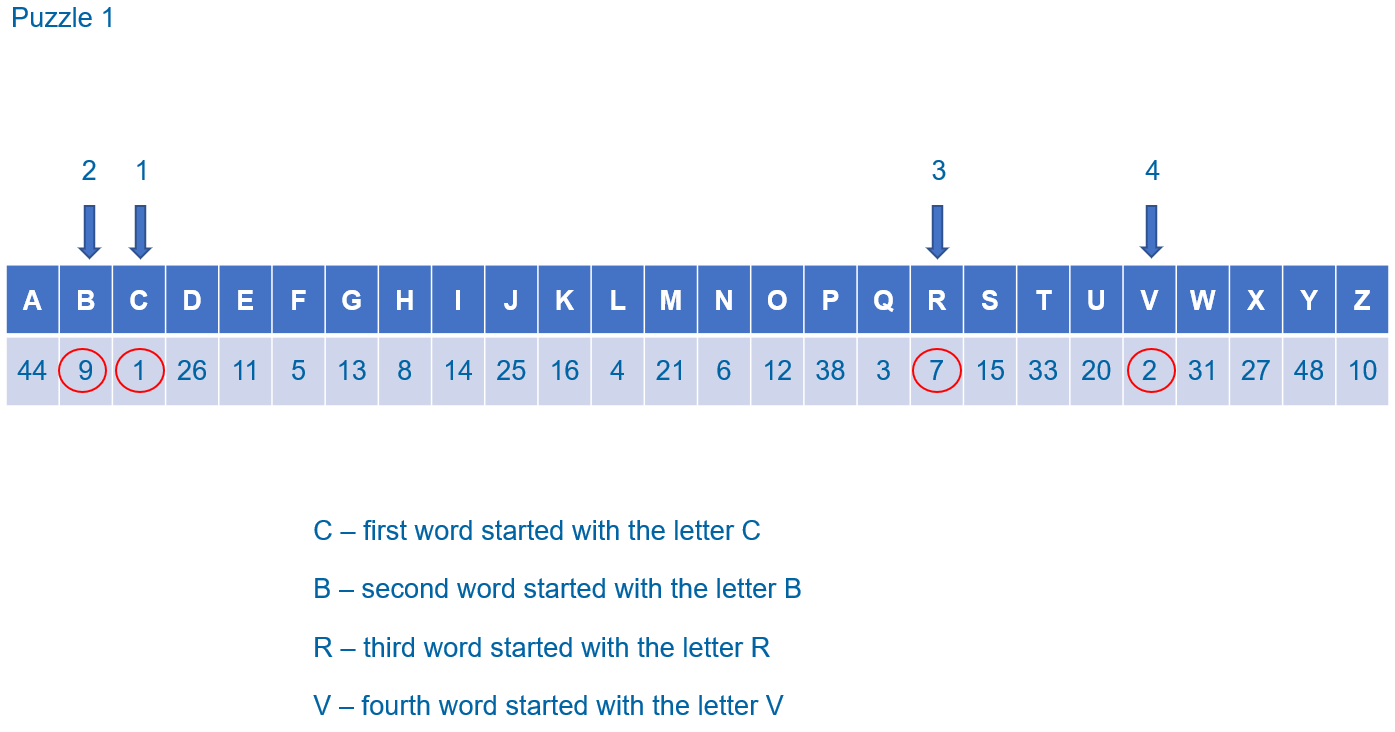


Puzzle number 1: Table cypher used to decode clues found in the fictional child abuse report.

**Puzzle 2: Child Abuse Stats and the Hidden Answers**

**Materials:** Wooden box locked with a 4-digit number combination. We set the lock to 1972. There is only one puzzle in this escape room with a 4-digit number lock combination. Inside the box is the UV light. Each statistic is printed on its own sheet of paper. We used the invisible ink to write the answers to the statistics on the sheets and then laminated them. The statistics written in invisible ink can be viewed using the UV light. The 8 different statistics are posted around the room on numbered cards. The numbers that open the next number combination lock are circled within the statistics. The order of the hidden numbers in the statistics is important in finding the correct 3-digit number combination of the next box. Refer to slide 5 in the Escape Room Puzzles PowerPoint.

**Instructions:** Puzzle number two is to find the hidden statistics written in invisible ink using the UV light.

1. The learners walk around the room shining their UV light on the statistics posted around the room.
2. Recognize that there are some numbers that are circled.
3. Recognize that the statistic cards are numbered in order.
4. That order will determine the sequence of the circled numbers that will open the next 3-digit number combination.


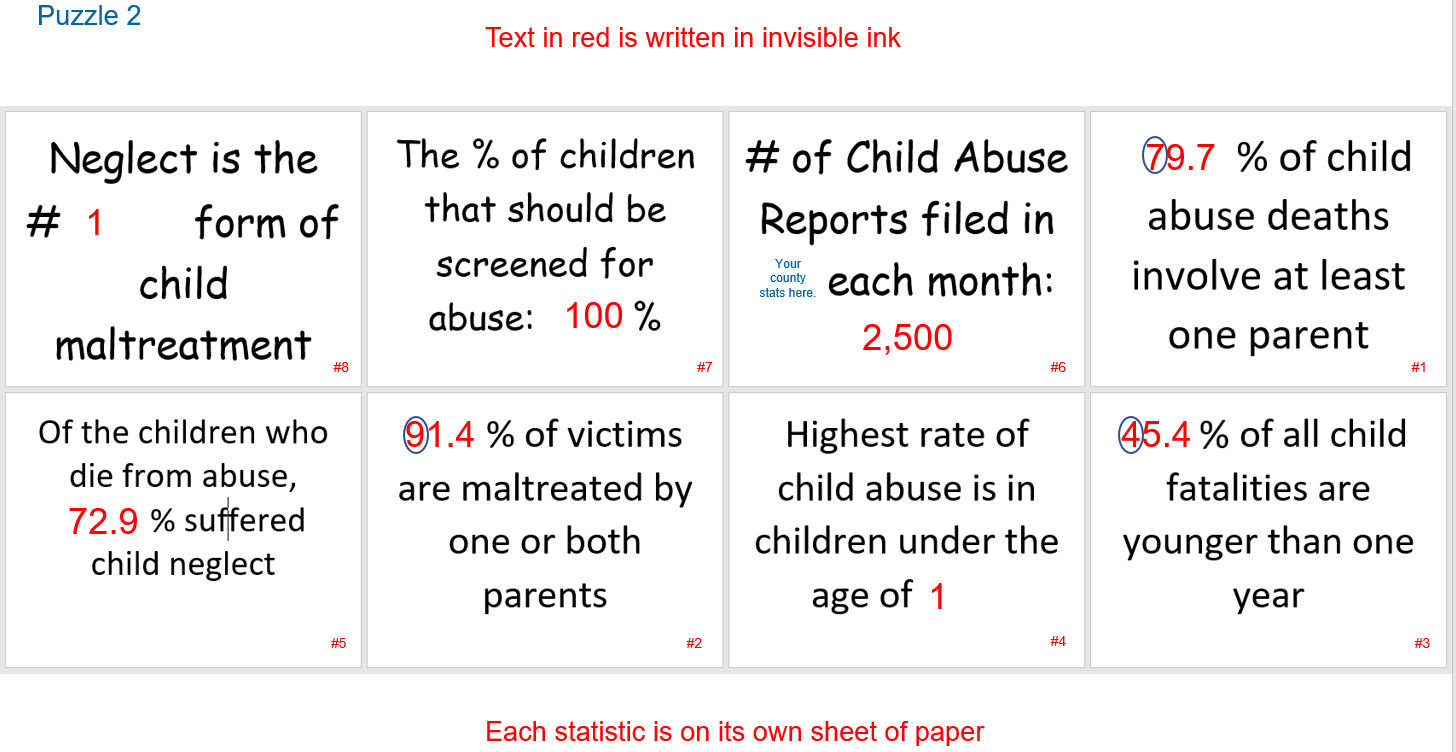


Puzzle number 2: Child abuse statistics with hidden codes to be place around the room.

**Puzzle 3: Local EMS Agency Policy on Suspected Child Abuse Reporting Guidelines**

**Materials:** We laminated the local EMS policy and the 3 questions that go along with the policy puzzle. Refer to slides 6-9 in the Escape Room Puzzles PowerPoint (several of these have blanks with PHI or county specific info that were removed for HIPAA). The laminated sheets are placed in the orange Plano marine dry box and locked with the 3-digit number combination lock.

**Instructions:** Puzzle number three is to find out information on local EMS reporting requirements.

1. Find within the policy the answer to the three questions and fill out the answers on the answer sheet.
2. Use the answer sheet to find the circled numbers that will open the next 3-digit number combination lock.


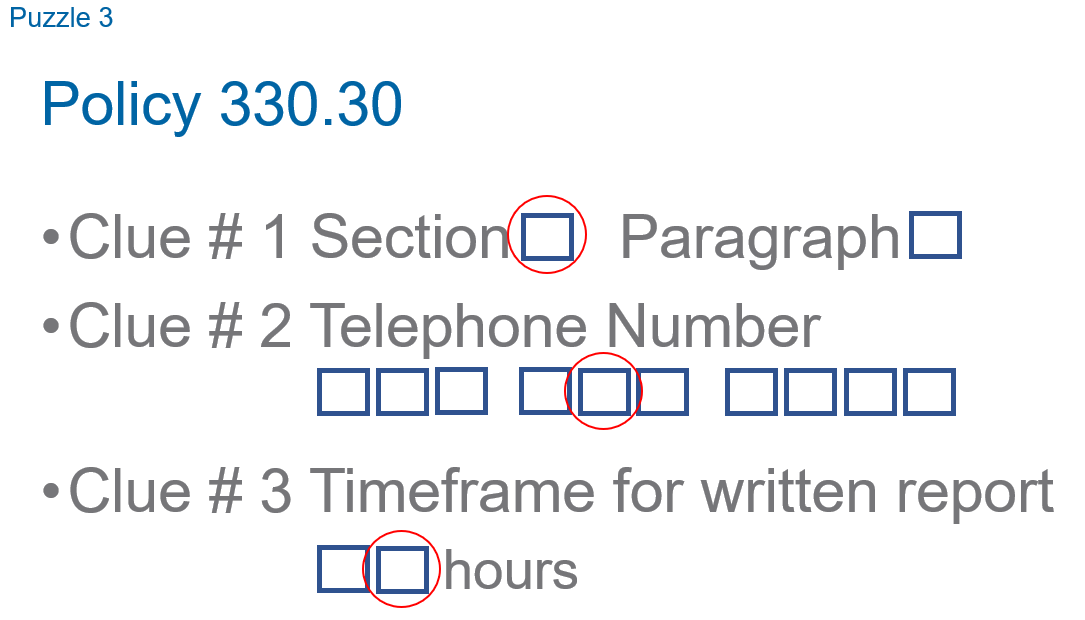


Puzzle number 3: Answer sheet.


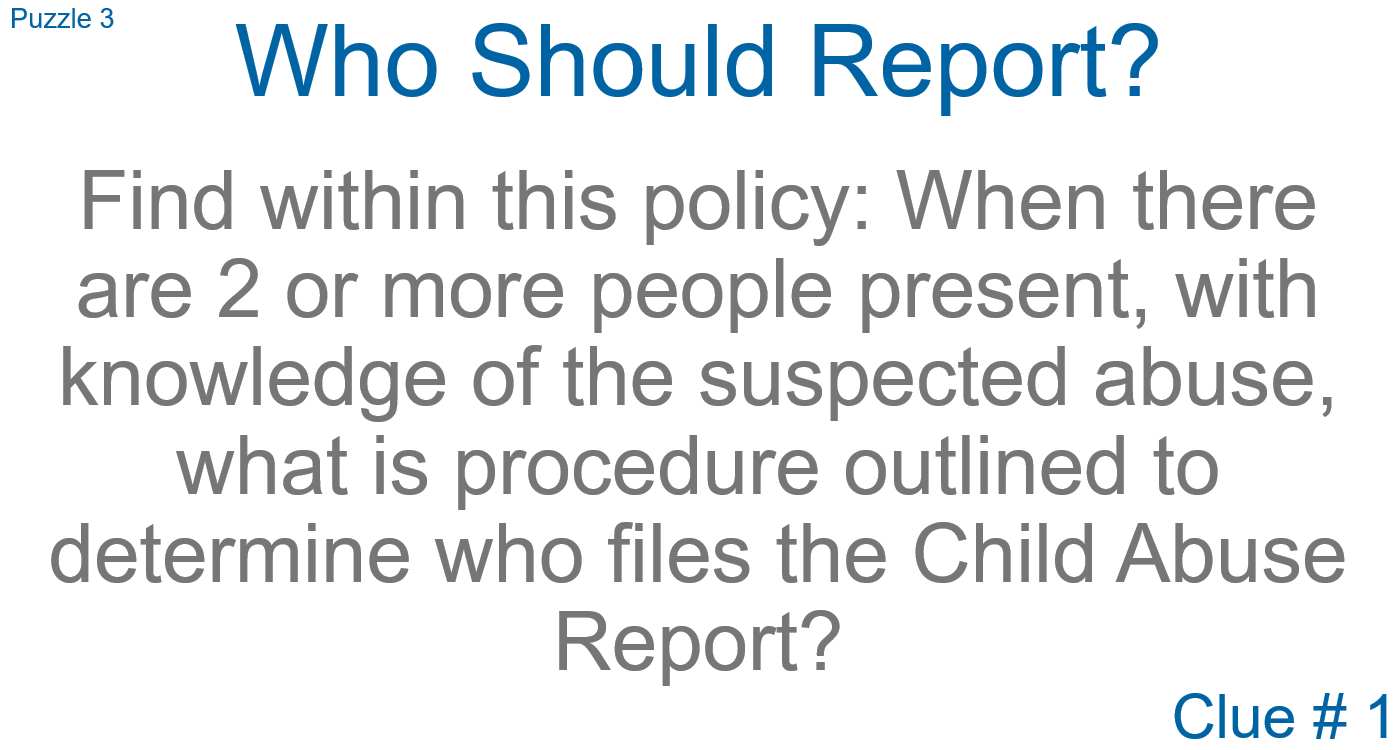


Puzzle number 3: Clue number 1.


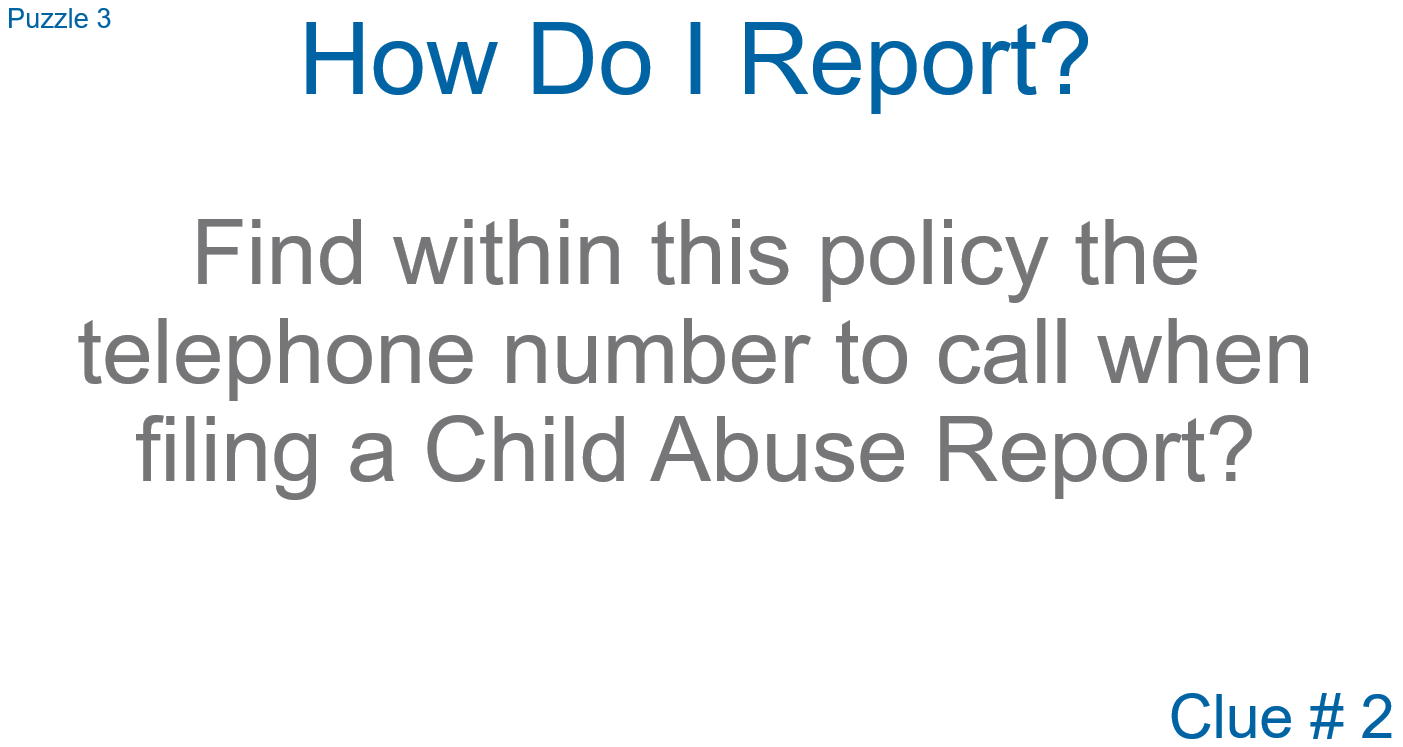


Puzzle number 3: Clue number 2.


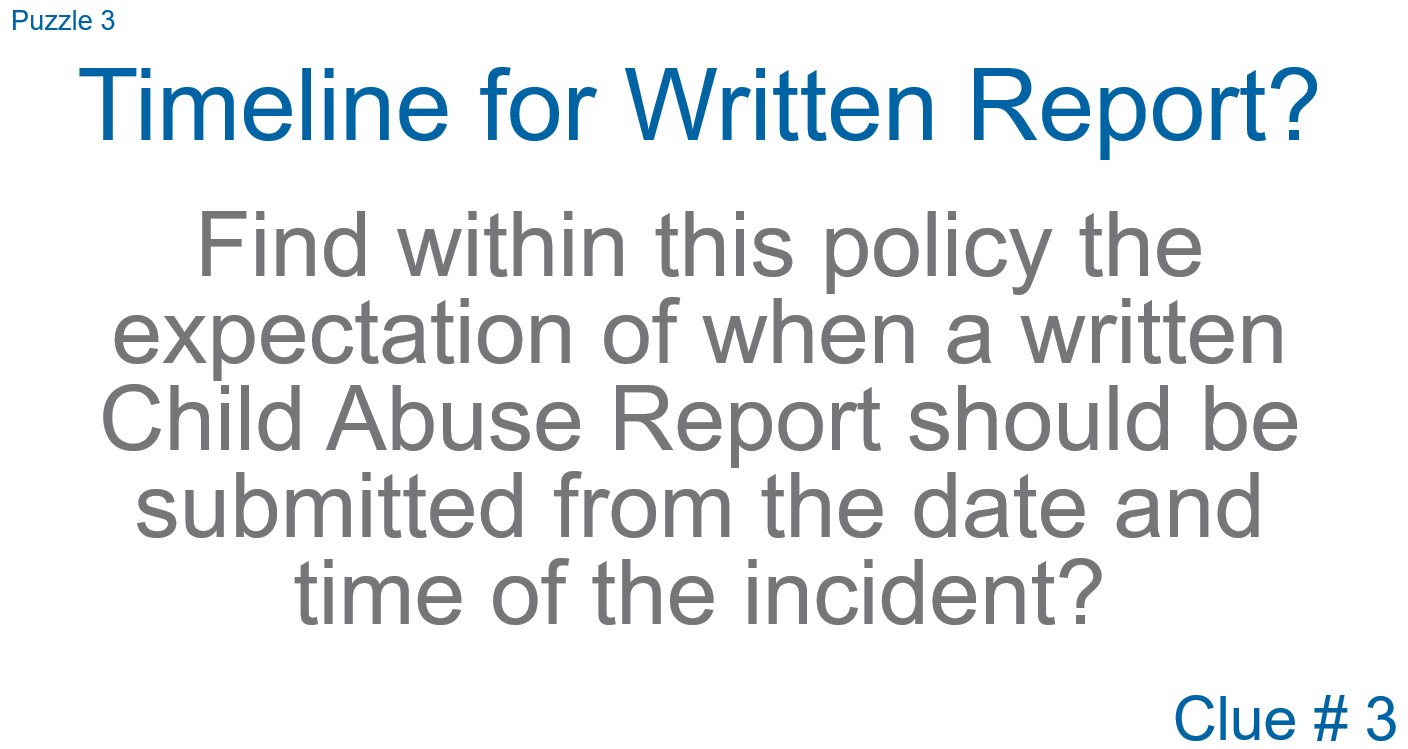


Puzzle number 3: Clue number 3.

**Puzzle 4: Shield Protector Case Studies**

**Materials:** We laminated the 6 case studies and the question sheet. We placed these documents in a brown envelope. The learner will also need the decoder wheel to solve the puzzle. Refer to slides 10-17 in the Escape Room Puzzles PowerPoint.

**Instructions:**

1. Answer the questions for each case study. The learners can circle their answers on the laminated sheet.
2. Use the decoder wheel to find the 6-lettered word on the answer sheet.


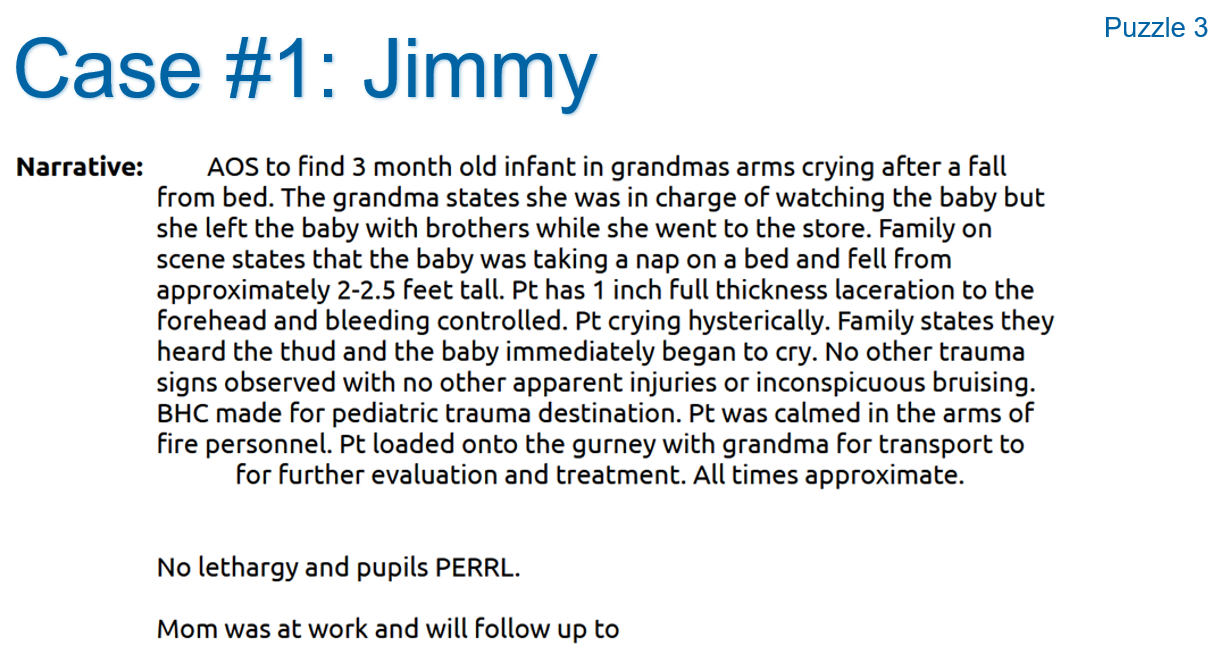


Puzzle number 4: Case study number 1.


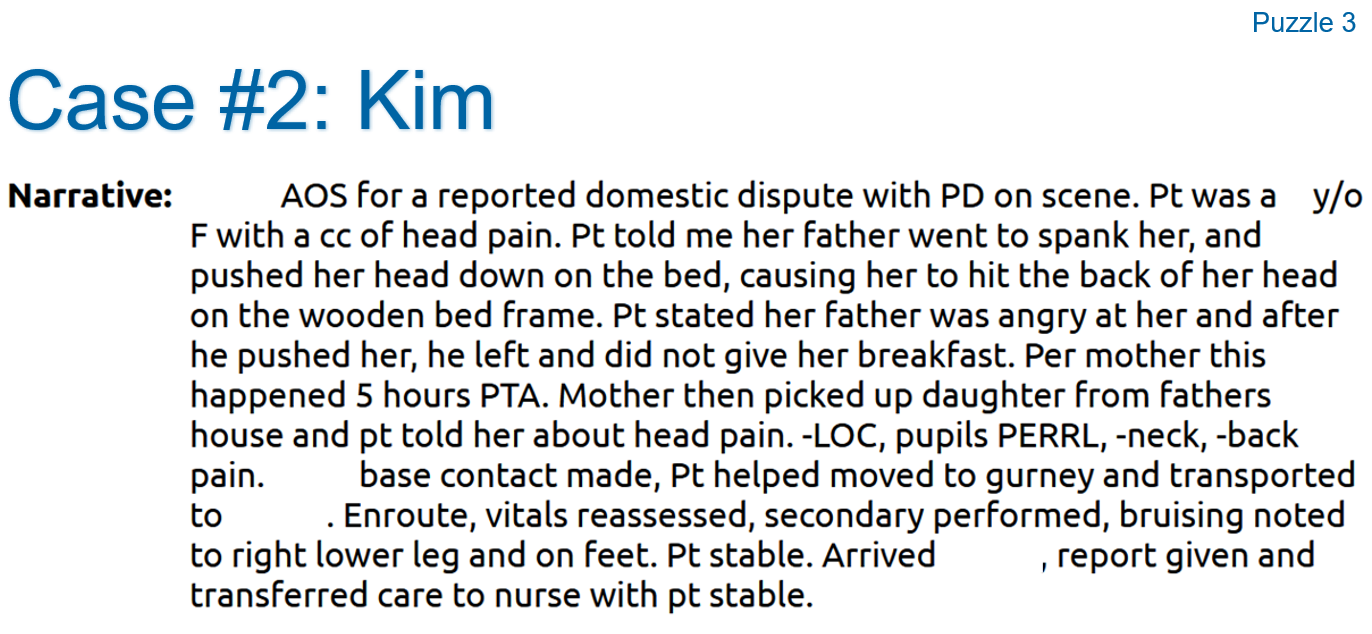


Puzzle number 4: Case study number 2.


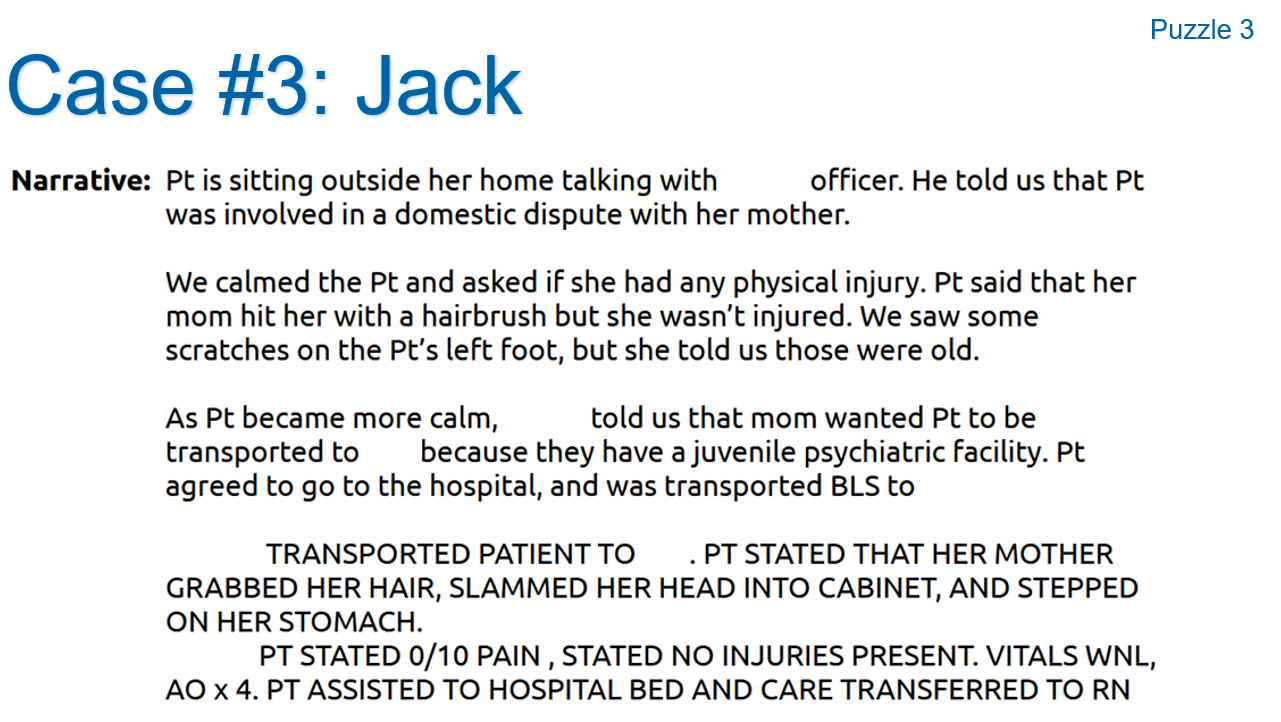


Puzzle number 4: Case study number 3.


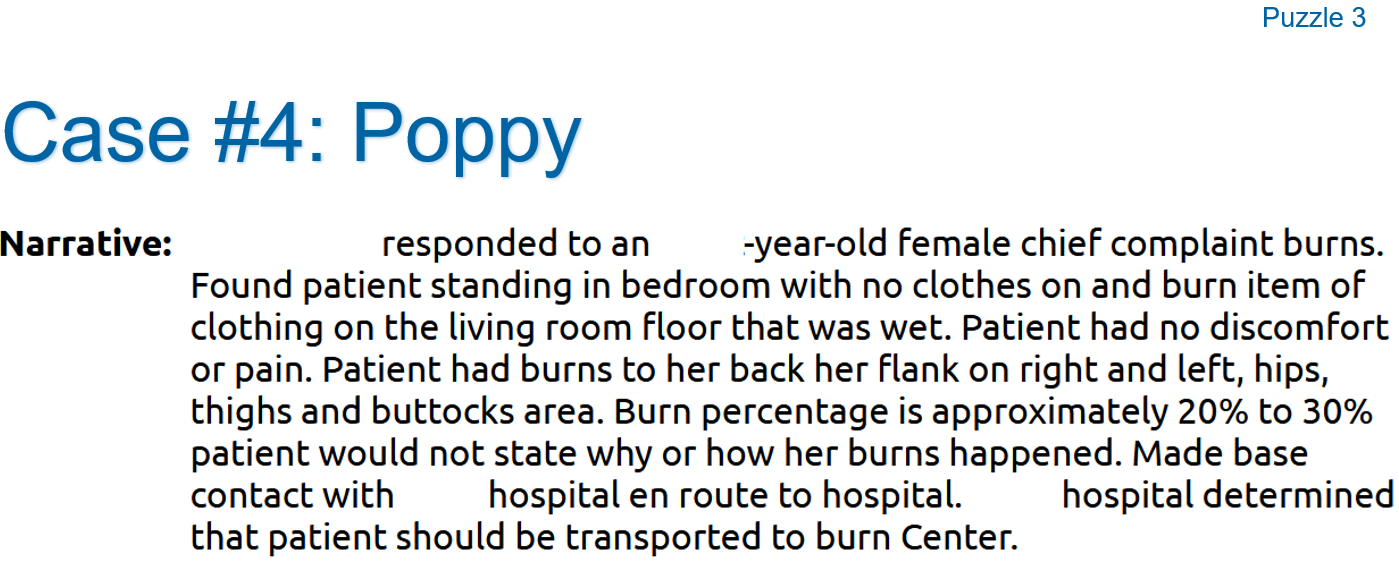


Puzzle number 4: Case study number 4.


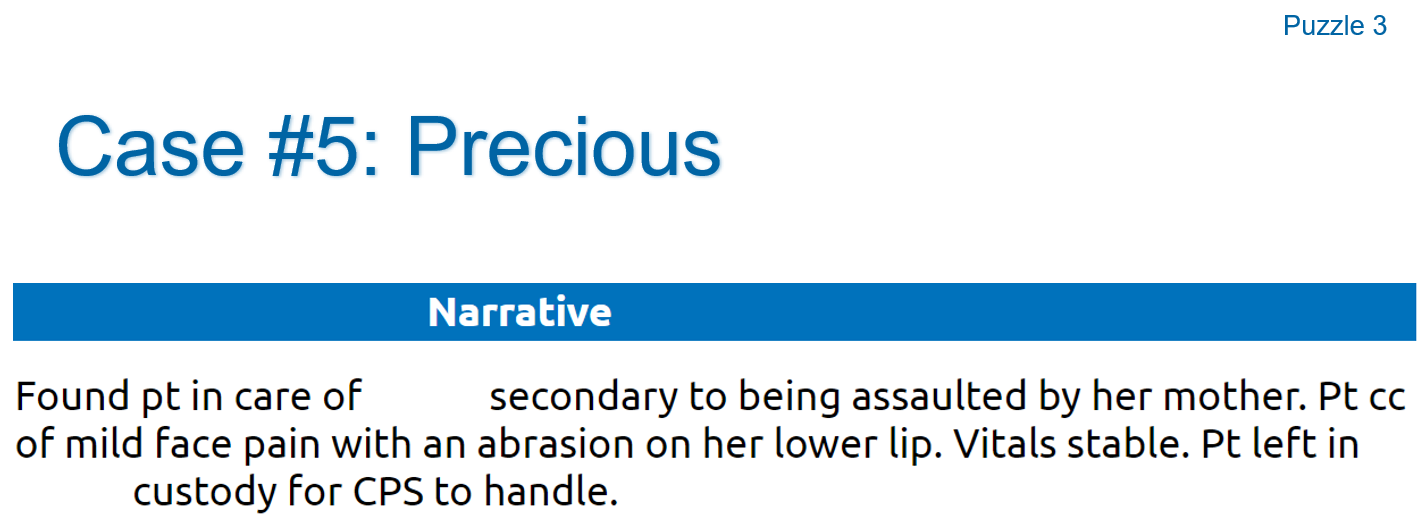


Puzzle number 4: Case study number 5.


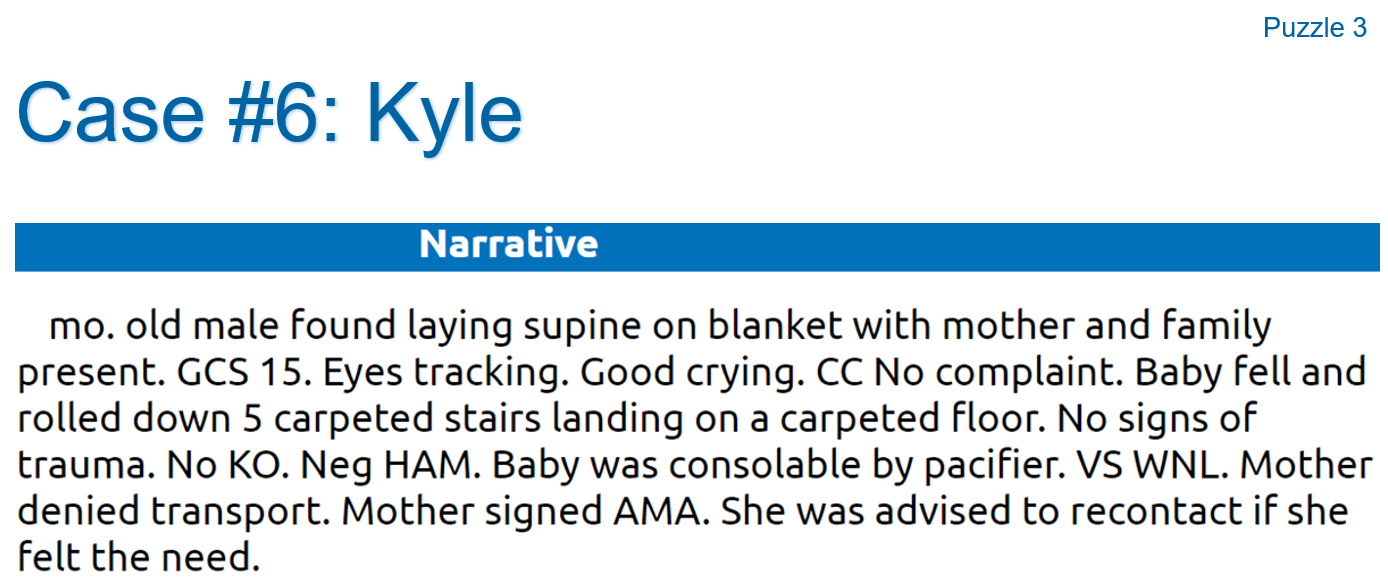


Puzzle number 4: Case study number 6.


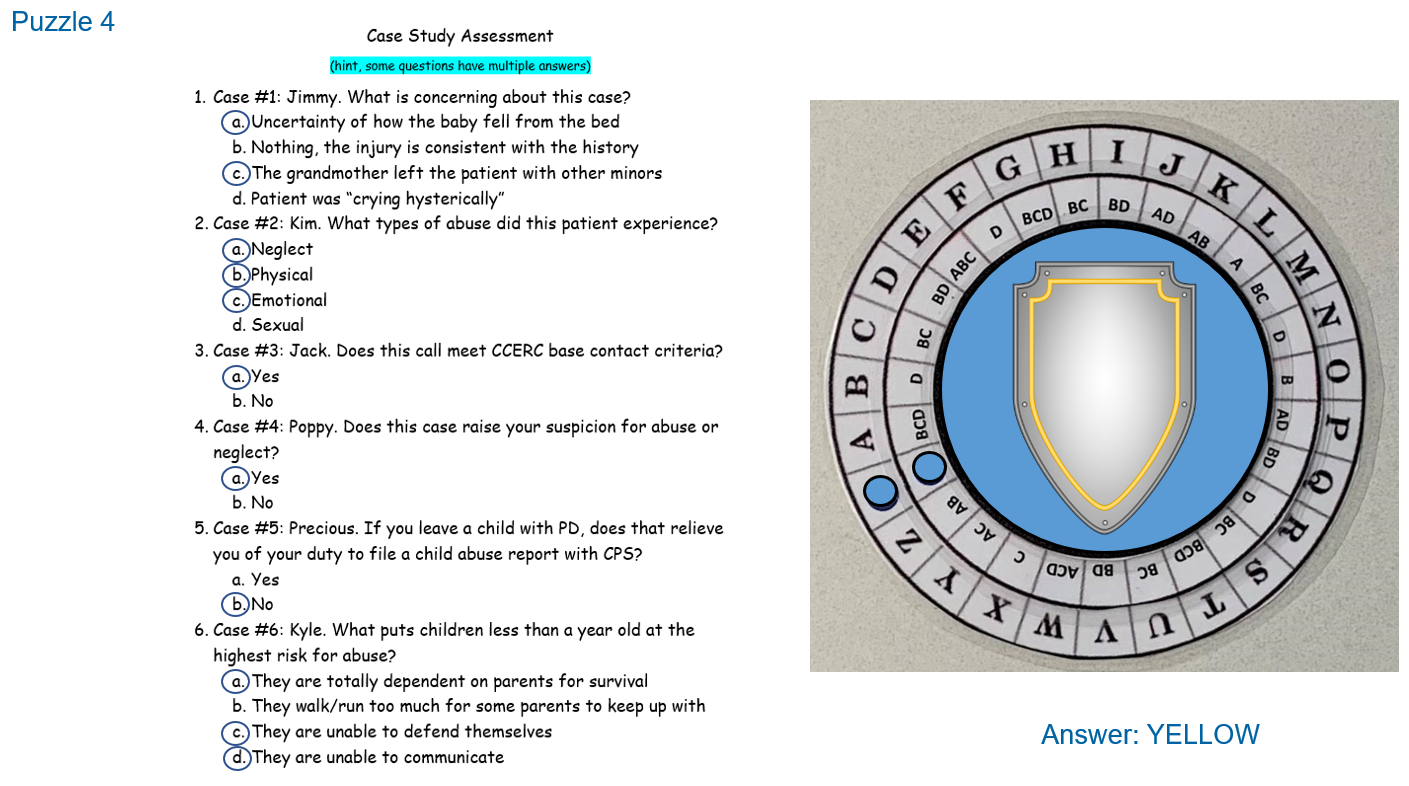


Puzzle number 4: Case study questions and decoder wheel.

**Puzzle 5: Yellow Play-doh Puzzle Clue**

**Materials:** On the table are different colored jars of Play-doh. The yellow Play-doh has a key hidden inside. The key opens the lock box with the puzzle pieces inside. You can draw or write a congratulatory message or a final take home point on the puzzle pieces.

**Instructions:**

1. The previous clue spells the word yellow. Inside the yellow Play-doh is a key that will unlock the final box.
2. The learner must open the box and successfully build the puzzle to escape the room.


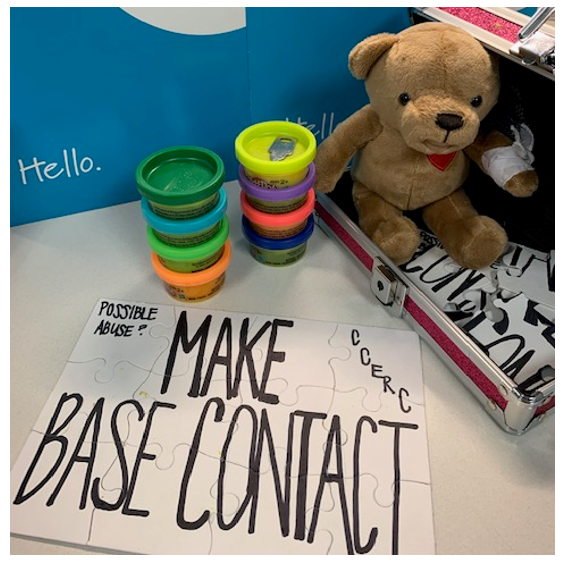


Puzzle number 5: The final puzzle solved
