# Supplementary material for: Child Maltreatment Education: Utilizing an Escape Room Activity to Engage Learners on a Sensitive Topic
Source: J Educ Teach Emerg Med. 2023 Jan 31;8(1):SG1–SG21. doi: 10.21980/J84H1C (PMC10332768; doi:10.21980/J84H1C)
Supplement: Supplementary file 4 [file jetem-8-1-sg1-appendixC.docx]

Appendix C:

Escape Room Answers

**Puzzle 1:** Use the decoder to find the selected 4 words. The first letter of each word corresponds with 1972 on the decoder.

**Puzzle 2:** 794

**Puzzle 3:**

- Question 1: Find within the policy: When there are 2 or more people present with knowledge of the suspected abuse, what is the procedure outlined to determine who files the Child Abuse Report? Answer: 1
- Question 2: Find within the policy the telephone number to call when filing a child abuse report. Answer is the 5^th^ number in the telephone number: 9.
- Question 3: Find within this policy your county’s EMS expectation of when a written child abuse report should be submitted from the date and time of the incident.

Answer is the 4 of the 24-hour answer.

**Puzzle 4:**

- Question 1: AC (Y)
- Question 2: ABC (E)
- Question 3: A (L)
- Question 4: A (L)
- Question 5: B (O)
- Question 6: ACD (W)

**Puzzle 5:** Build the puzzle
