# Supplementary material for: Child Maltreatment Education: Utilizing an Escape Room Activity to Engage Learners on a Sensitive Topic
Source: J Educ Teach Emerg Med. 2023 Jan 31;8(1):SG1–SG21. doi: 10.21980/J84H1C (PMC10332768; doi:10.21980/J84H1C)
Supplement: Supplementary file 5 [file jetem-8-1-sg1-appendixD.pptx]

## Slide 1
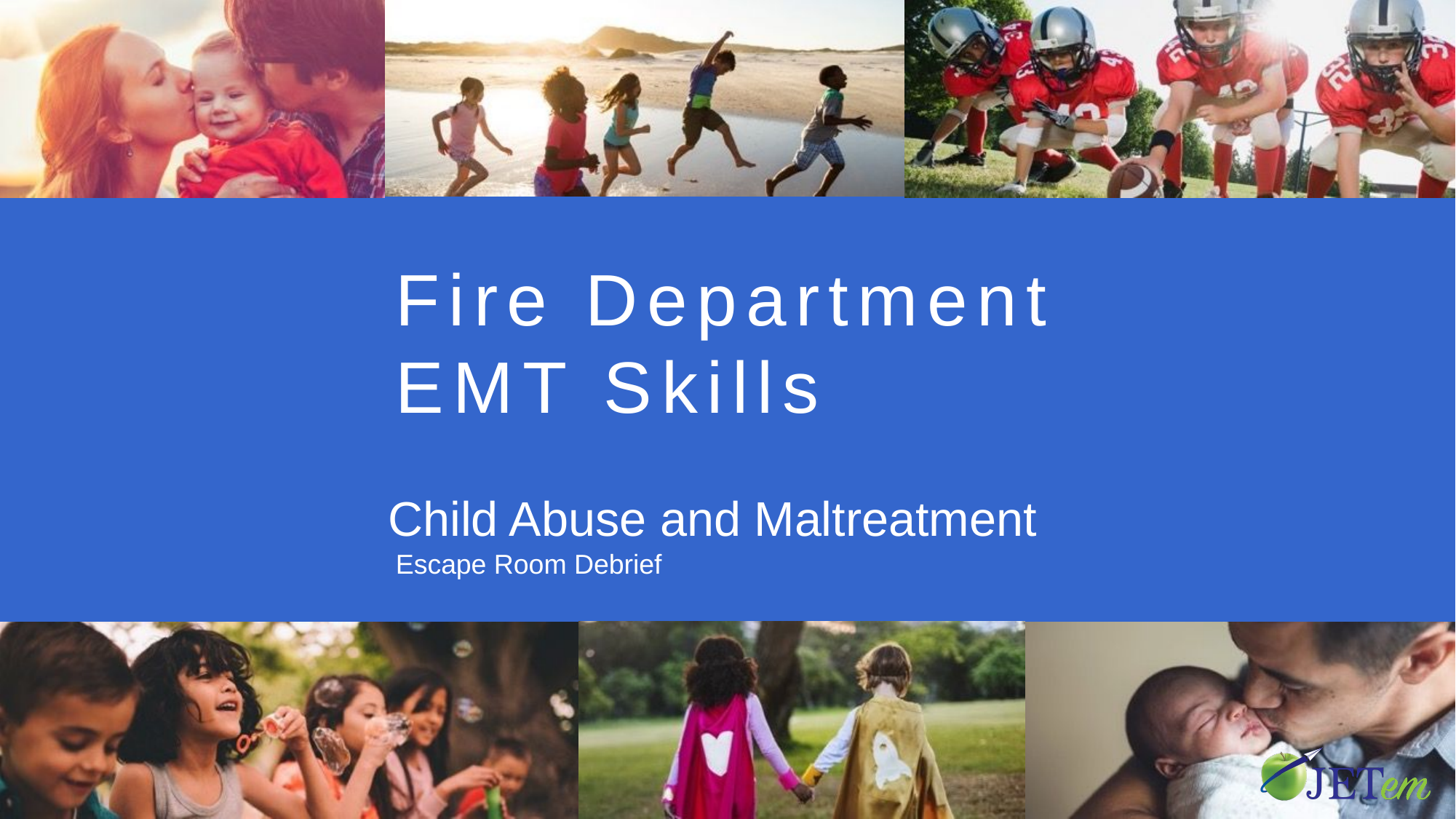

# Fire Department EMT Skills
Child Abuse and Maltreatment
Escape Room Debrief

## Slide 2
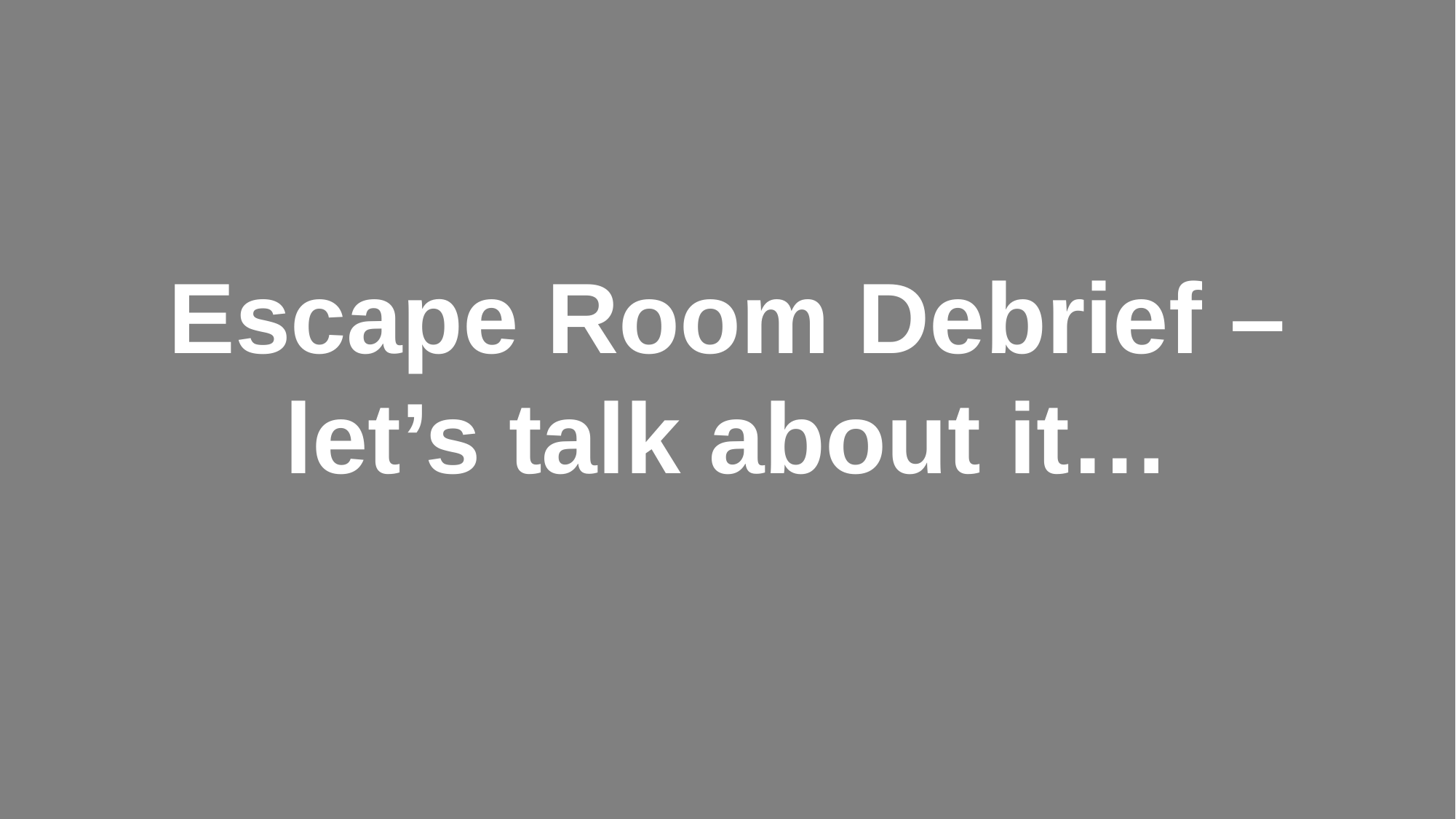

# Escape Room Debrief – let’s talk about it…

## Slide 3
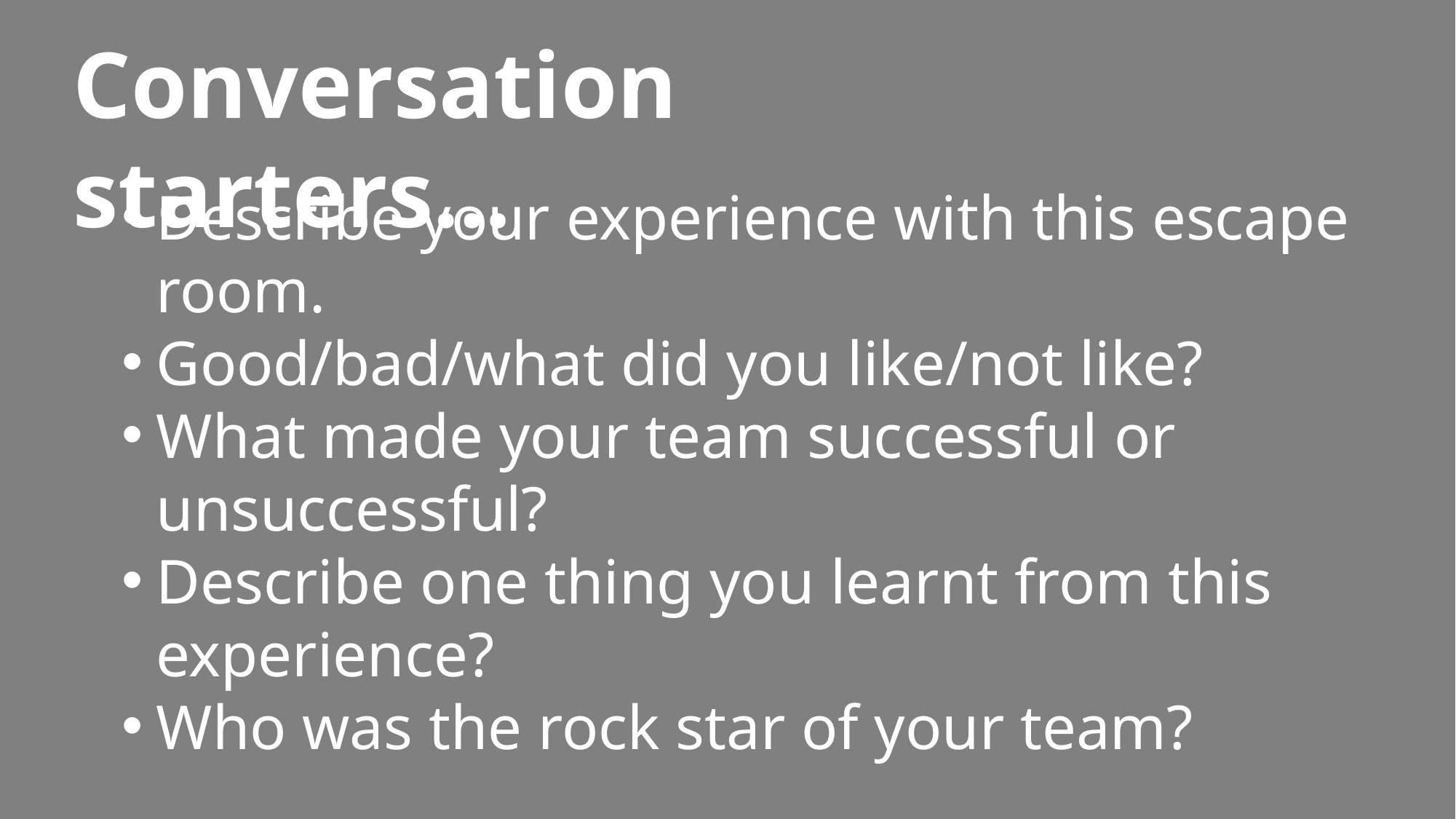

Conversation starters…
Describe your experience with this escape room.
Good/bad/what did you like/not like?
What made your team successful or unsuccessful?
Describe one thing you learnt from this experience?
Who was the rock star of your team?

## Slide 4
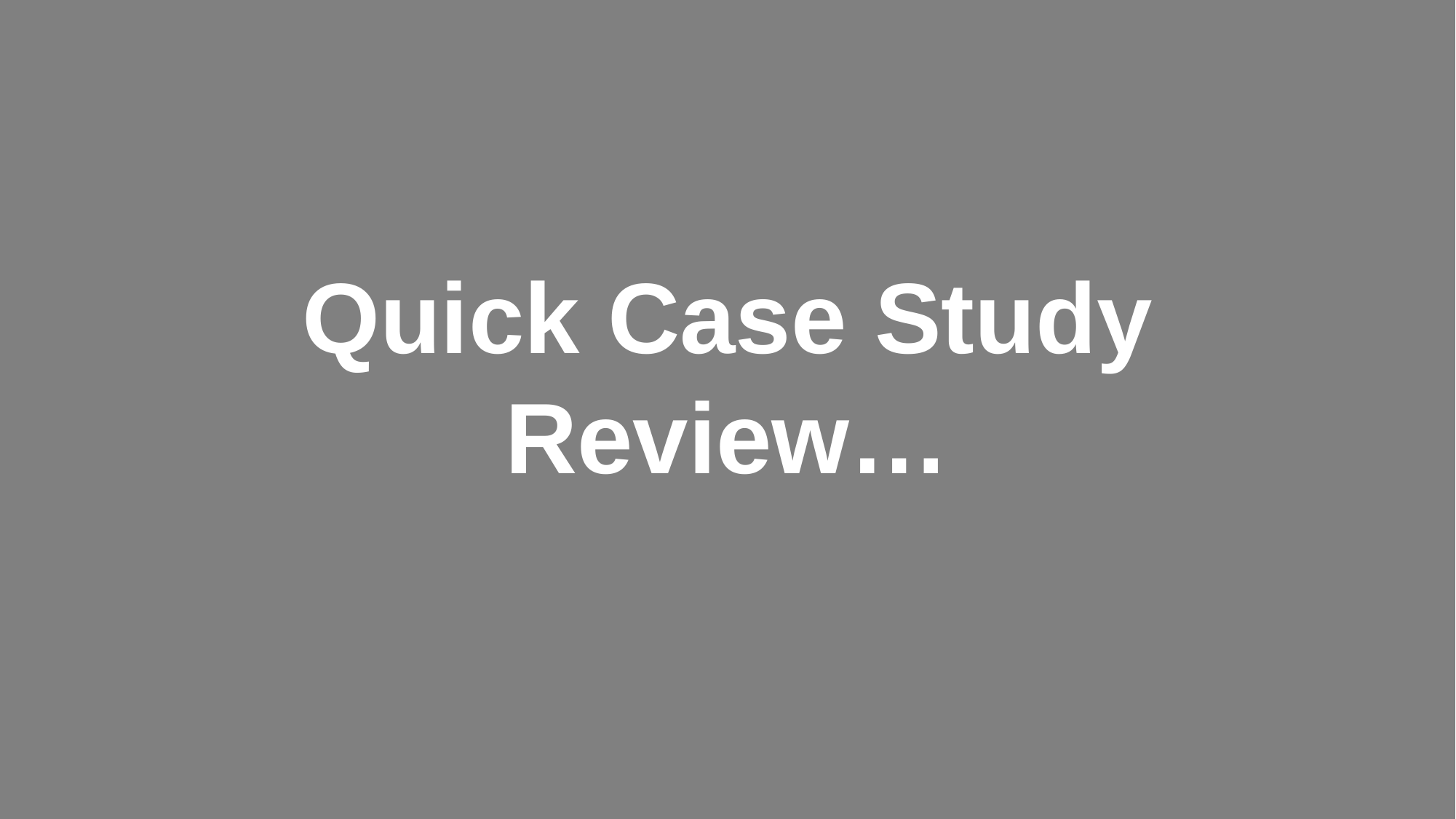

# Quick Case Study Review…

## Slide 5
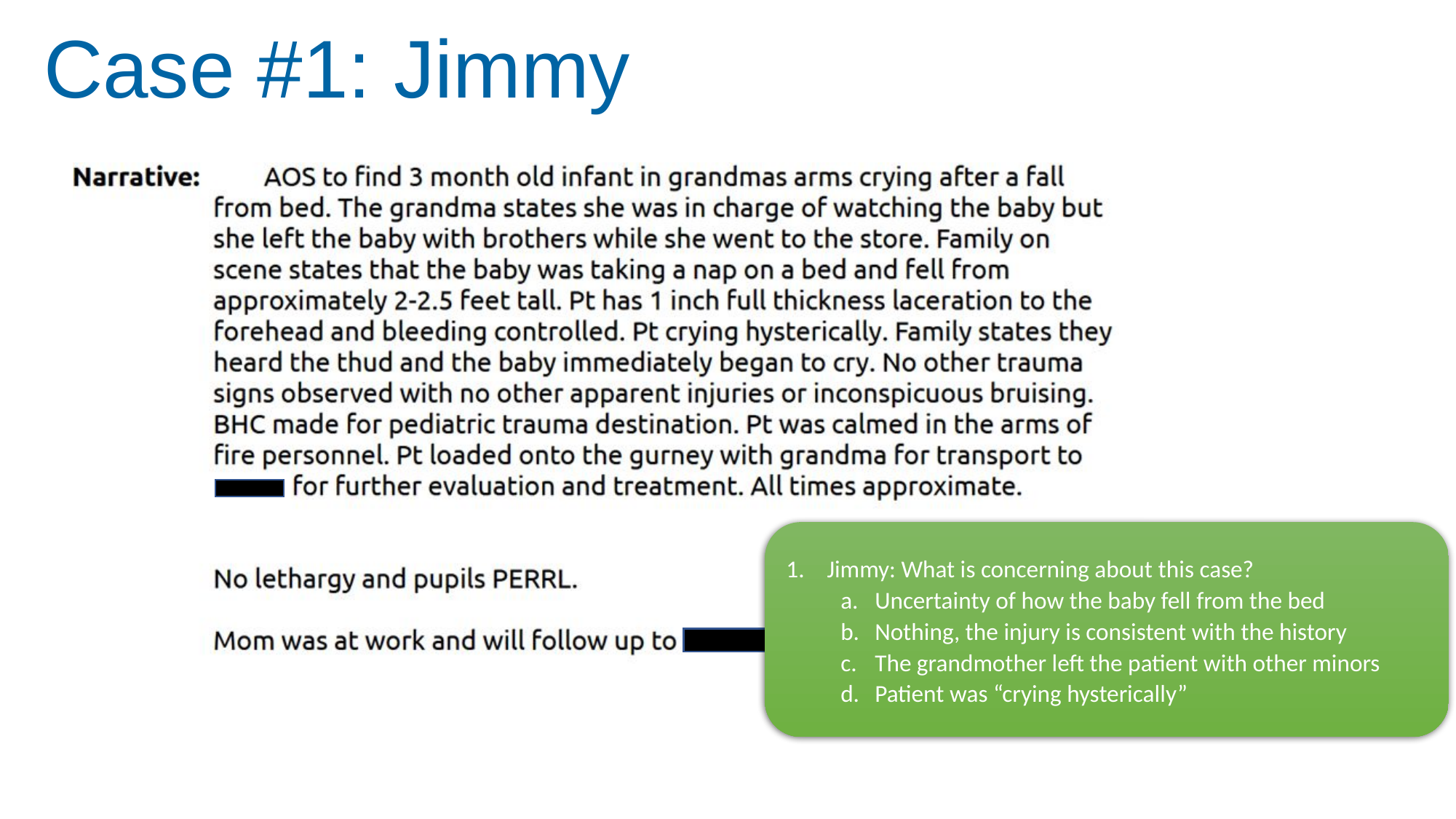

Case #1: Jimmy
Jimmy: What is concerning about this case?
Uncertainty of how the baby fell from the bed
Nothing, the injury is consistent with the history
The grandmother left the patient with other minors
Patient was “crying hysterically”

## Slide 6
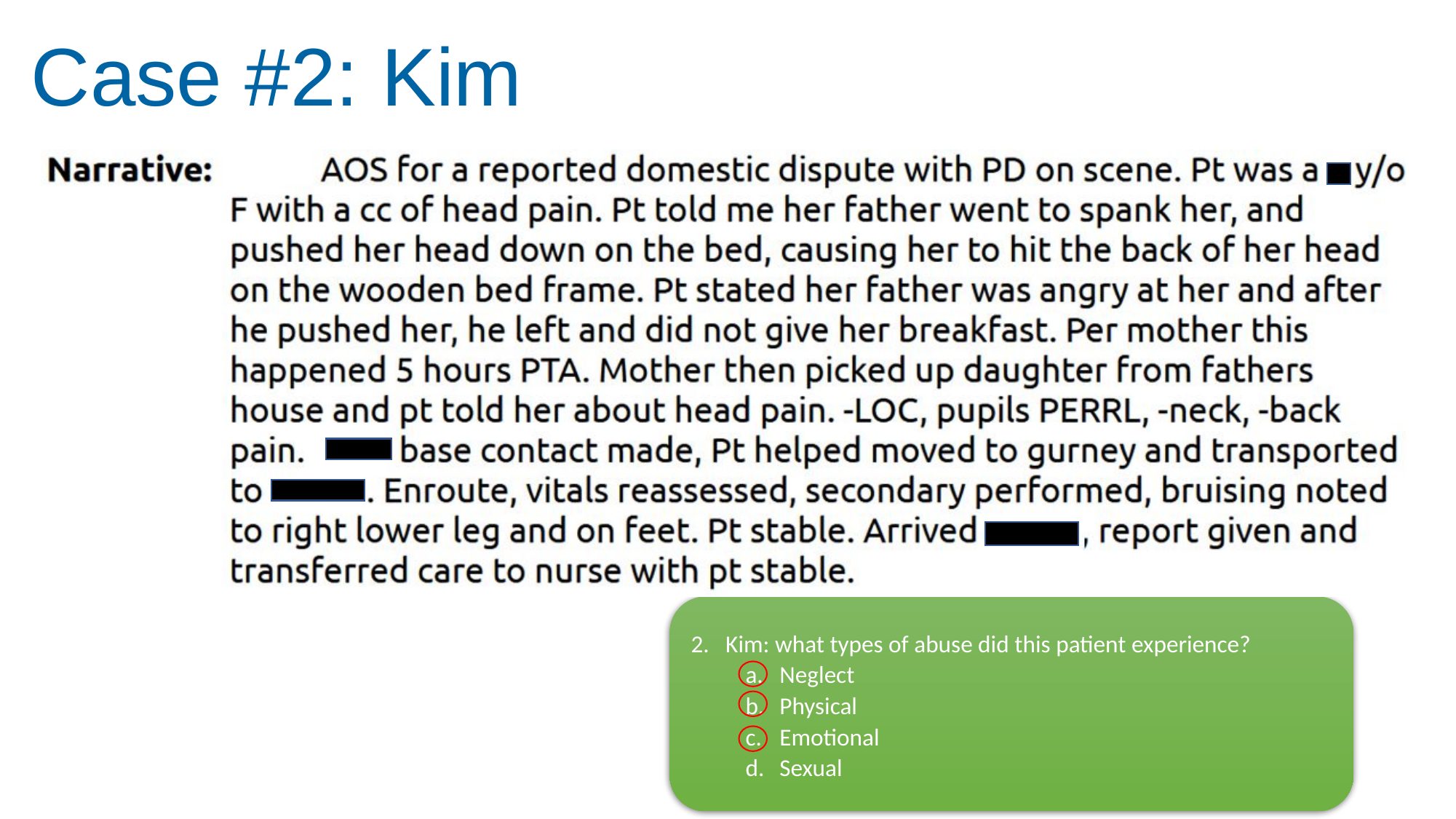

Case #2: Kim
2. Kim: what types of abuse did this patient experience?
Neglect
Physical
Emotional
Sexual

## Slide 7
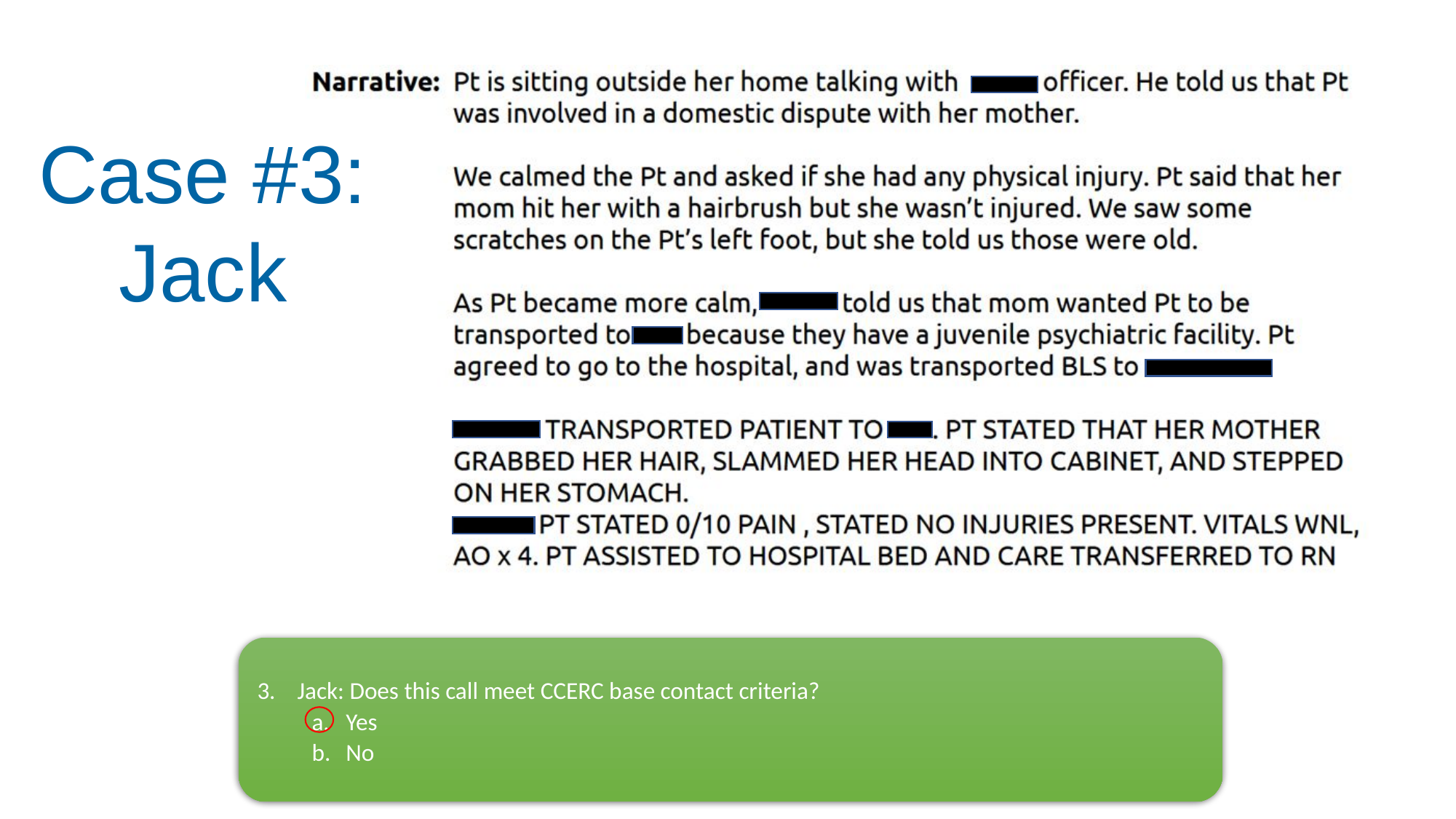

Case #3: Jack
3. Jack: Does this call meet CCERC base contact criteria?
Yes
No

## Slide 8
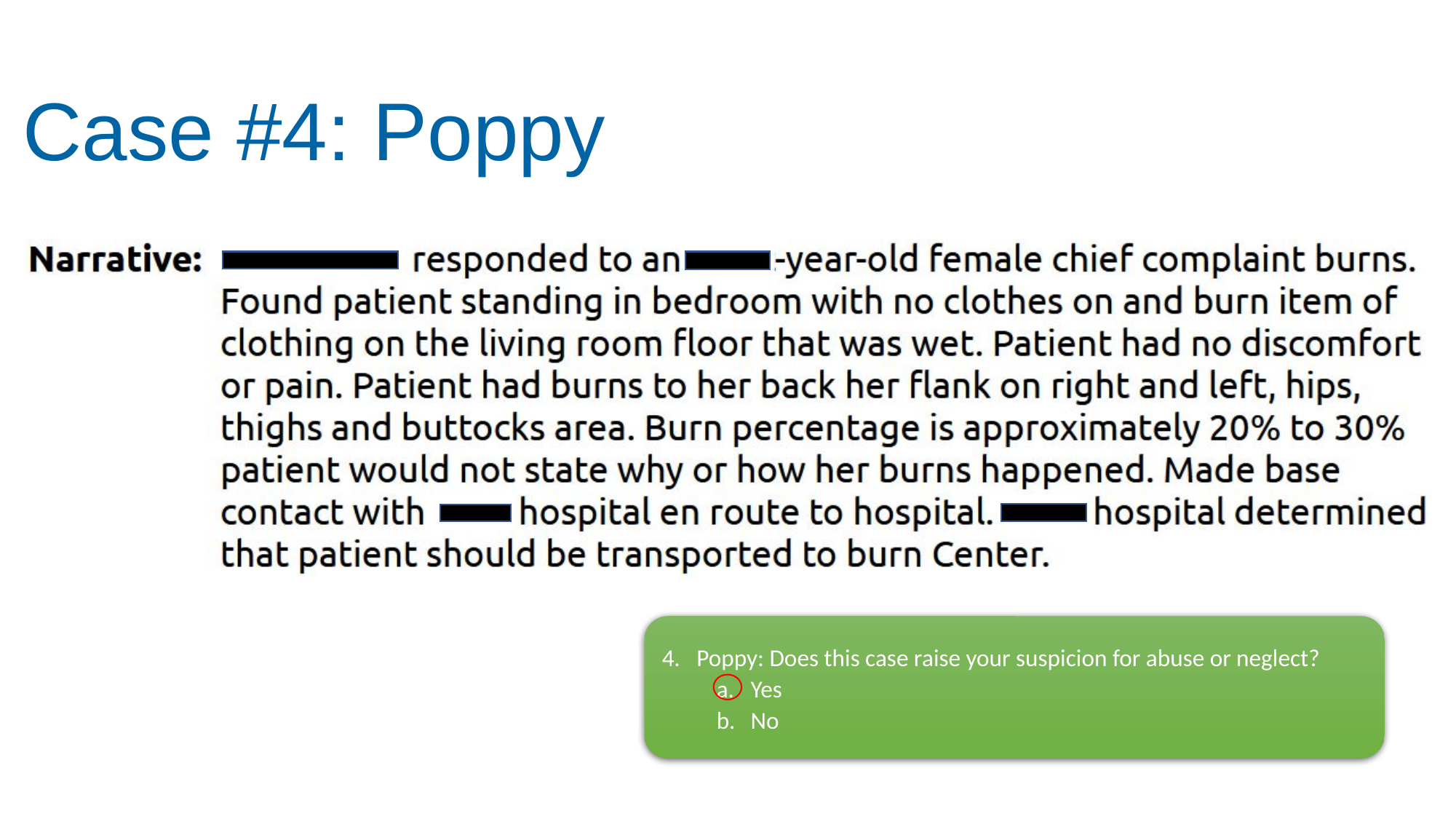

Case #4: Poppy
4. Poppy: Does this case raise your suspicion for abuse or neglect?
Yes
No

## Slide 9
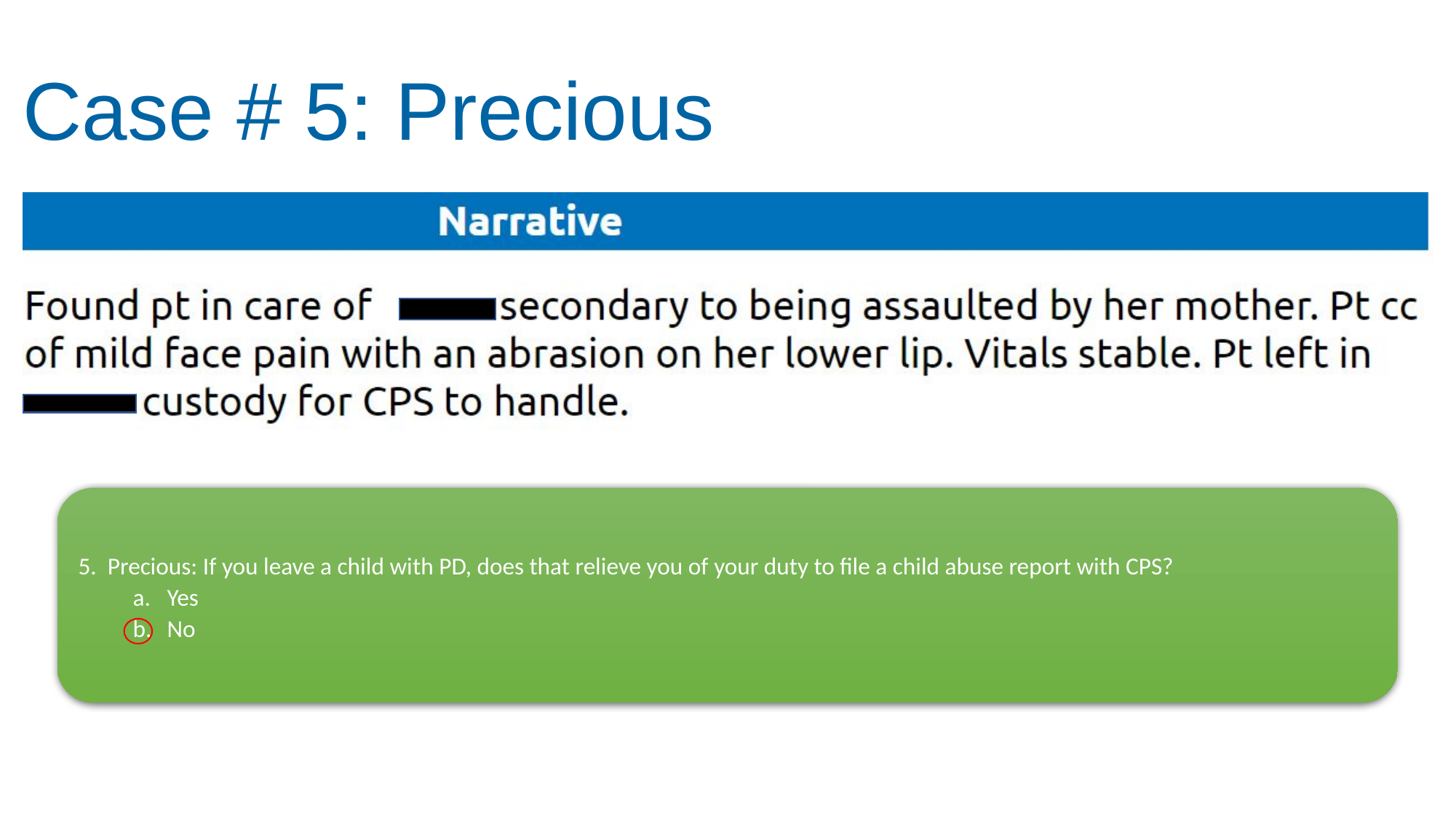

Case # 5: Precious
5. Precious: If you leave a child with PD, does that relieve you of your duty to file a child abuse report with CPS?
Yes
No

## Slide 10
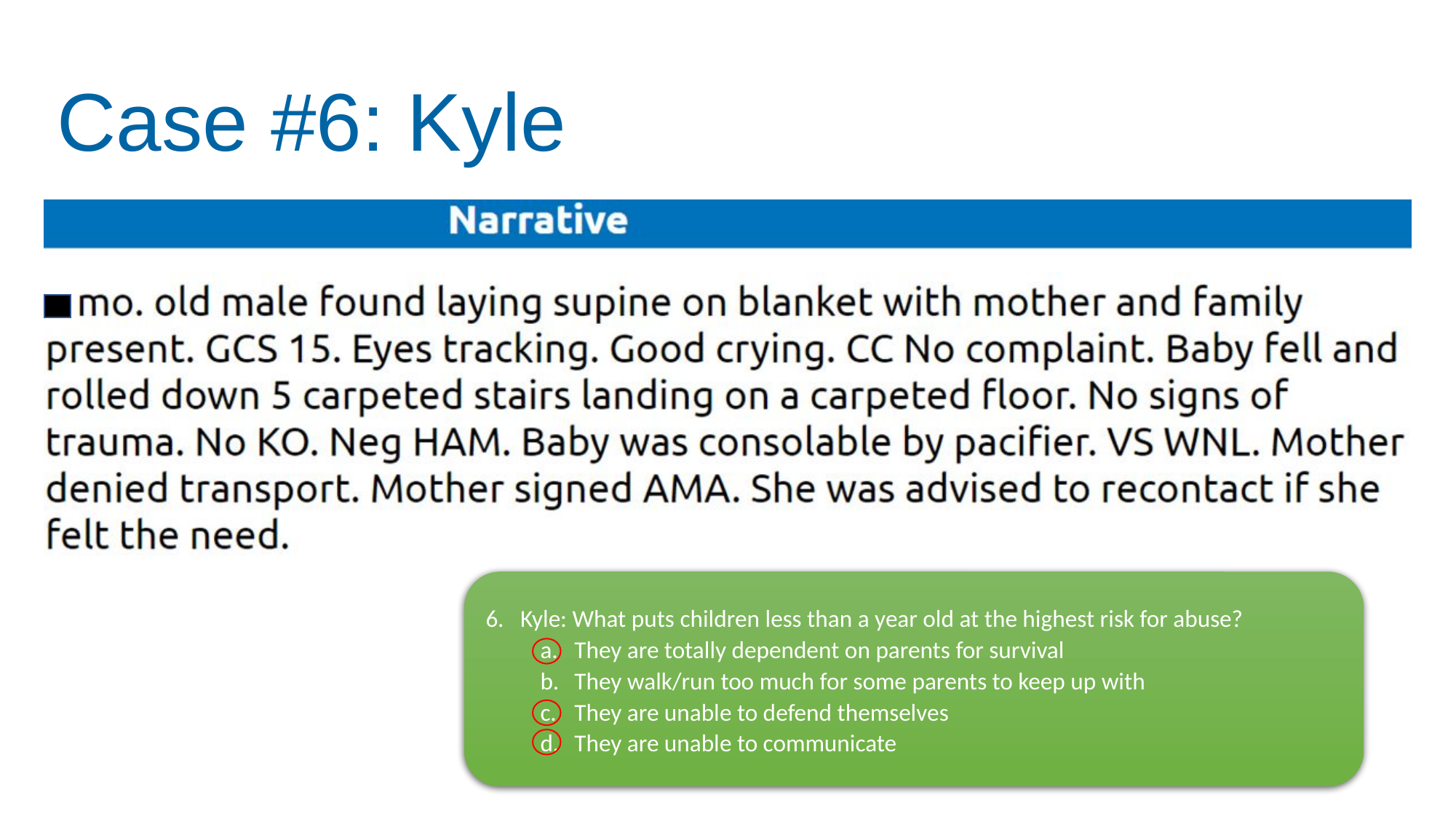

Case #6: Kyle
6. Kyle: What puts children less than a year old at the highest risk for abuse?
They are totally dependent on parents for survival
They walk/run too much for some parents to keep up with
They are unable to defend themselves
They are unable to communicate

## Slide 11
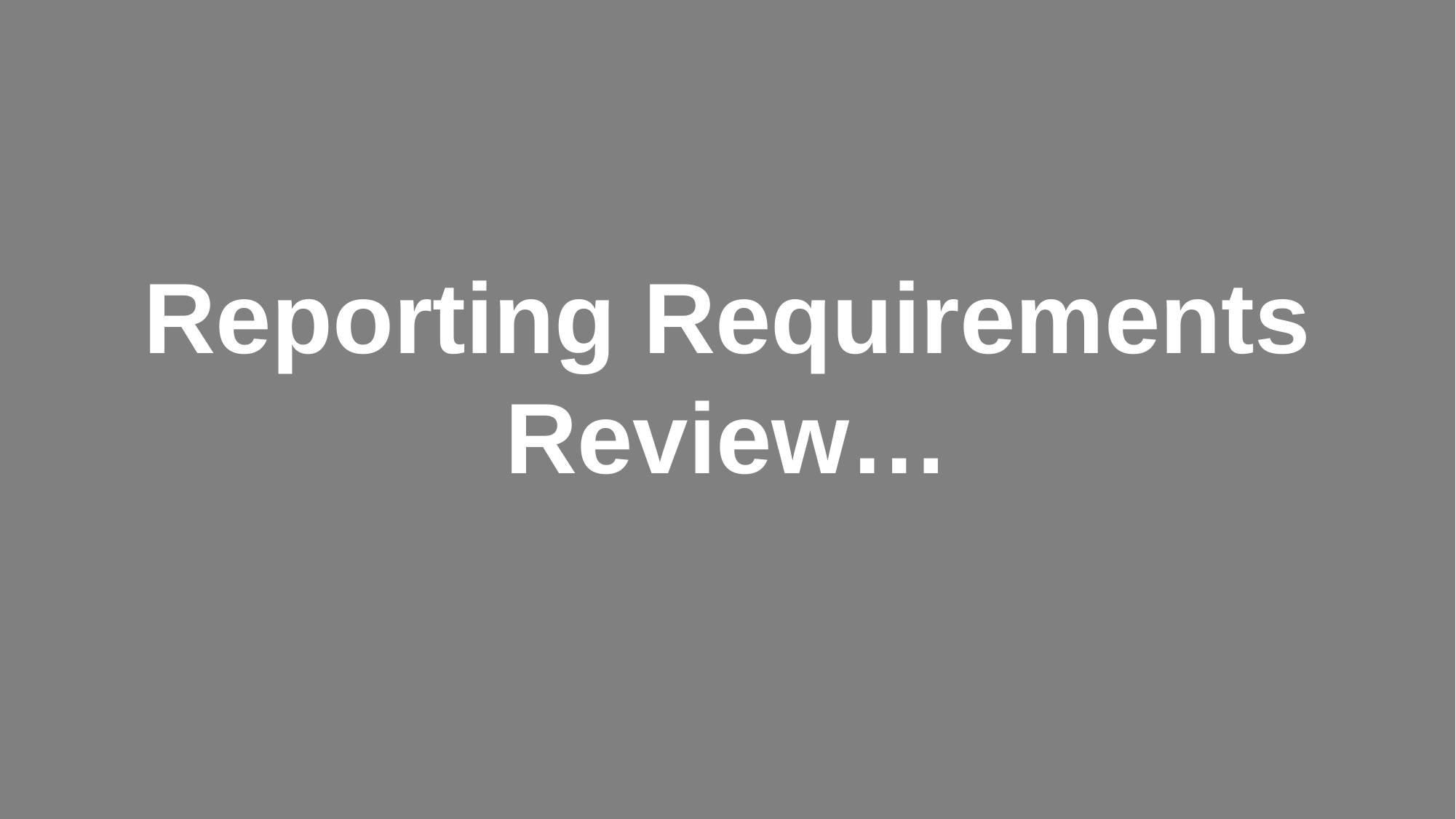

# Reporting Requirements Review…

## Slide 12
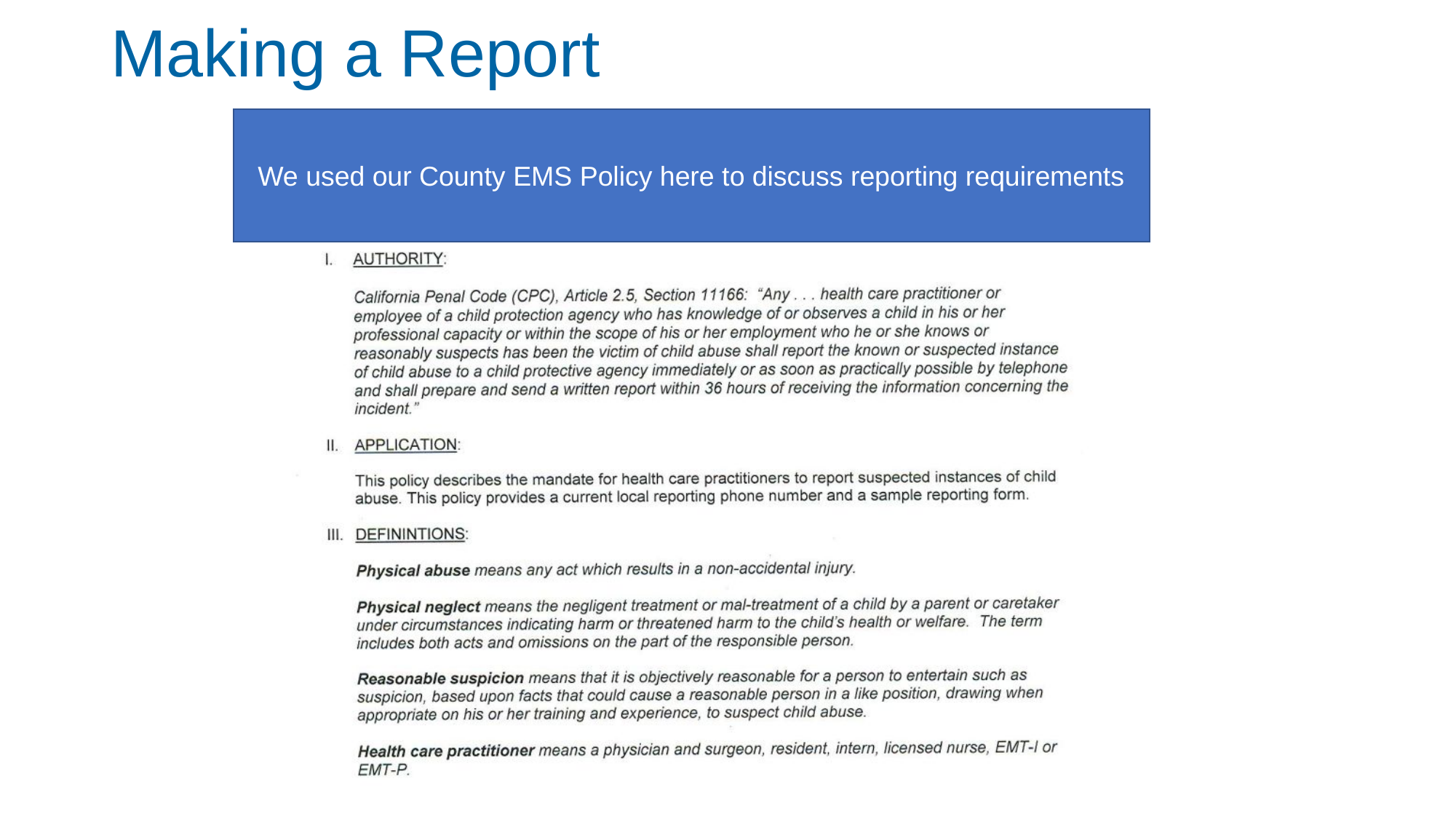

# Making a Report
We used our County EMS Policy here to discuss reporting requirements

## Slide 13
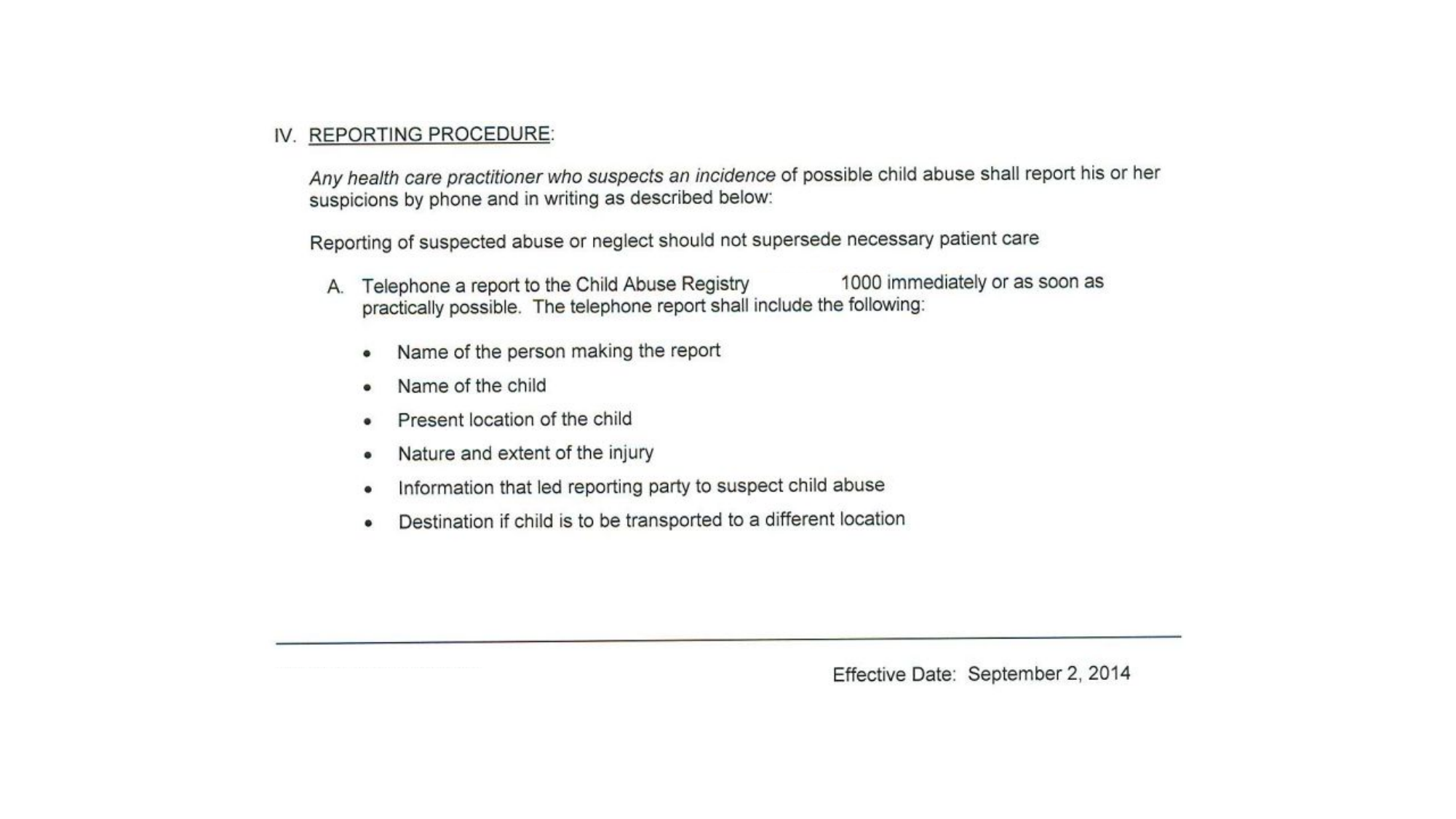

## Slide 14
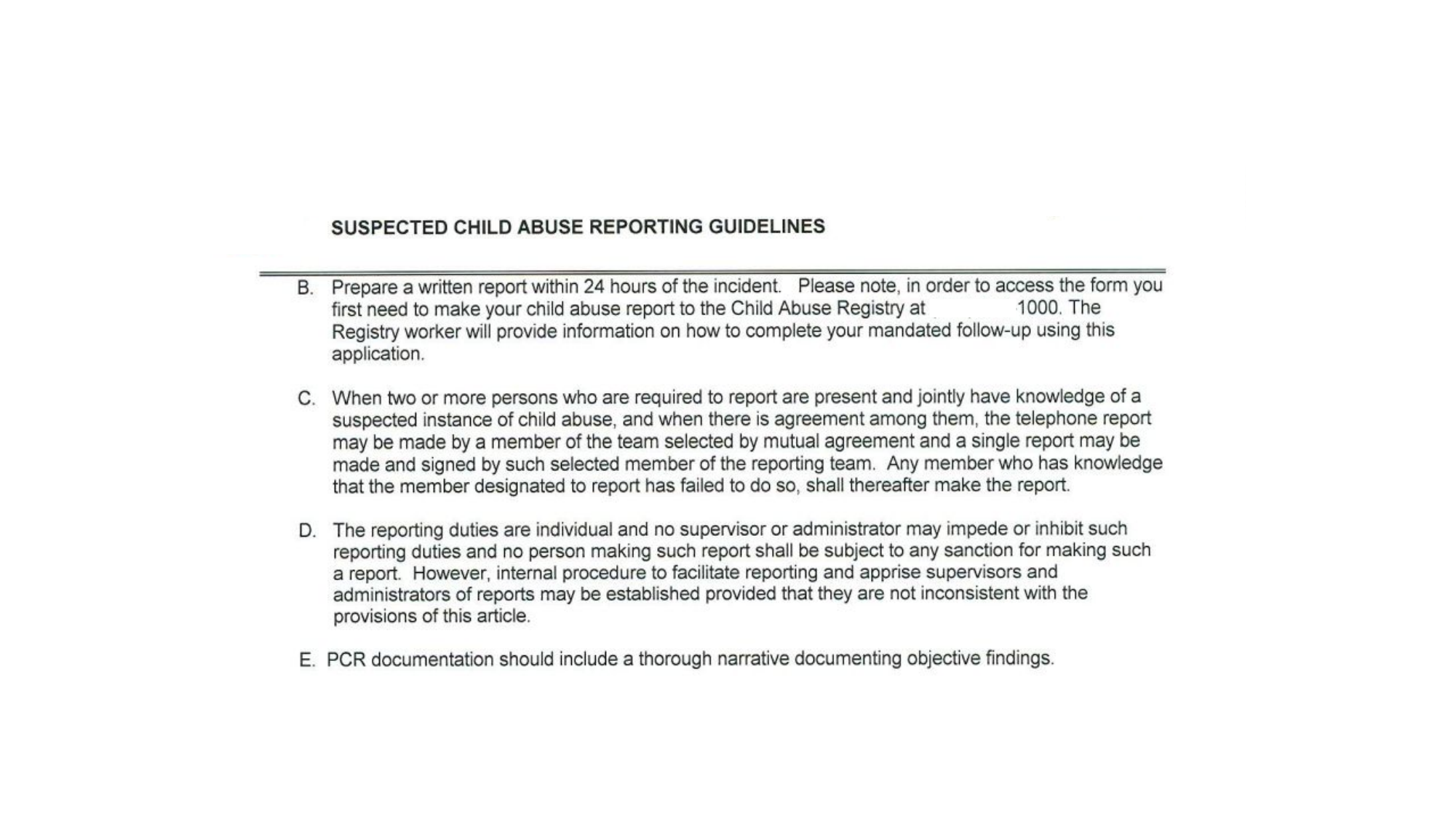

## Slide 15
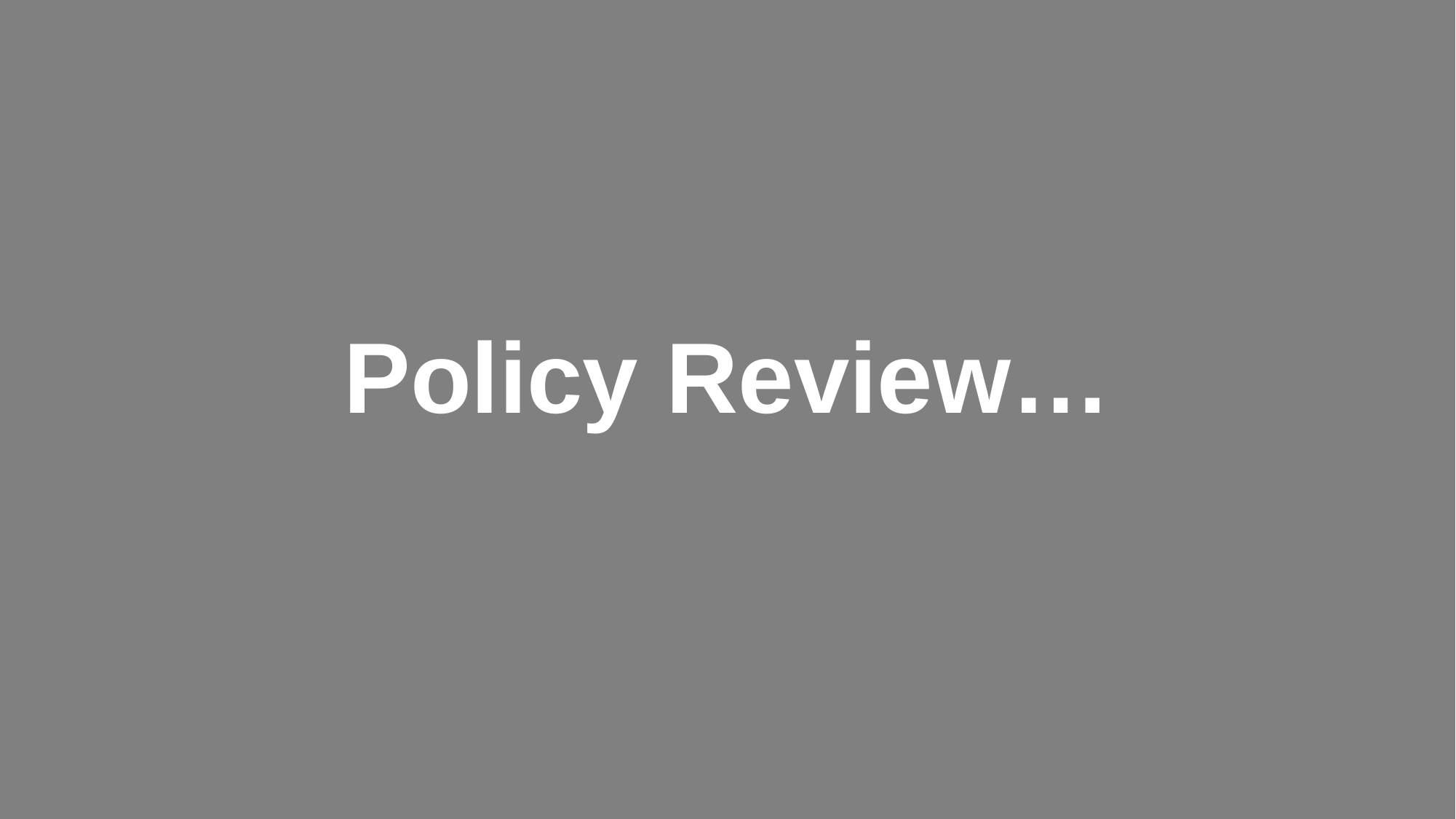

# Policy Review…

## Slide 16
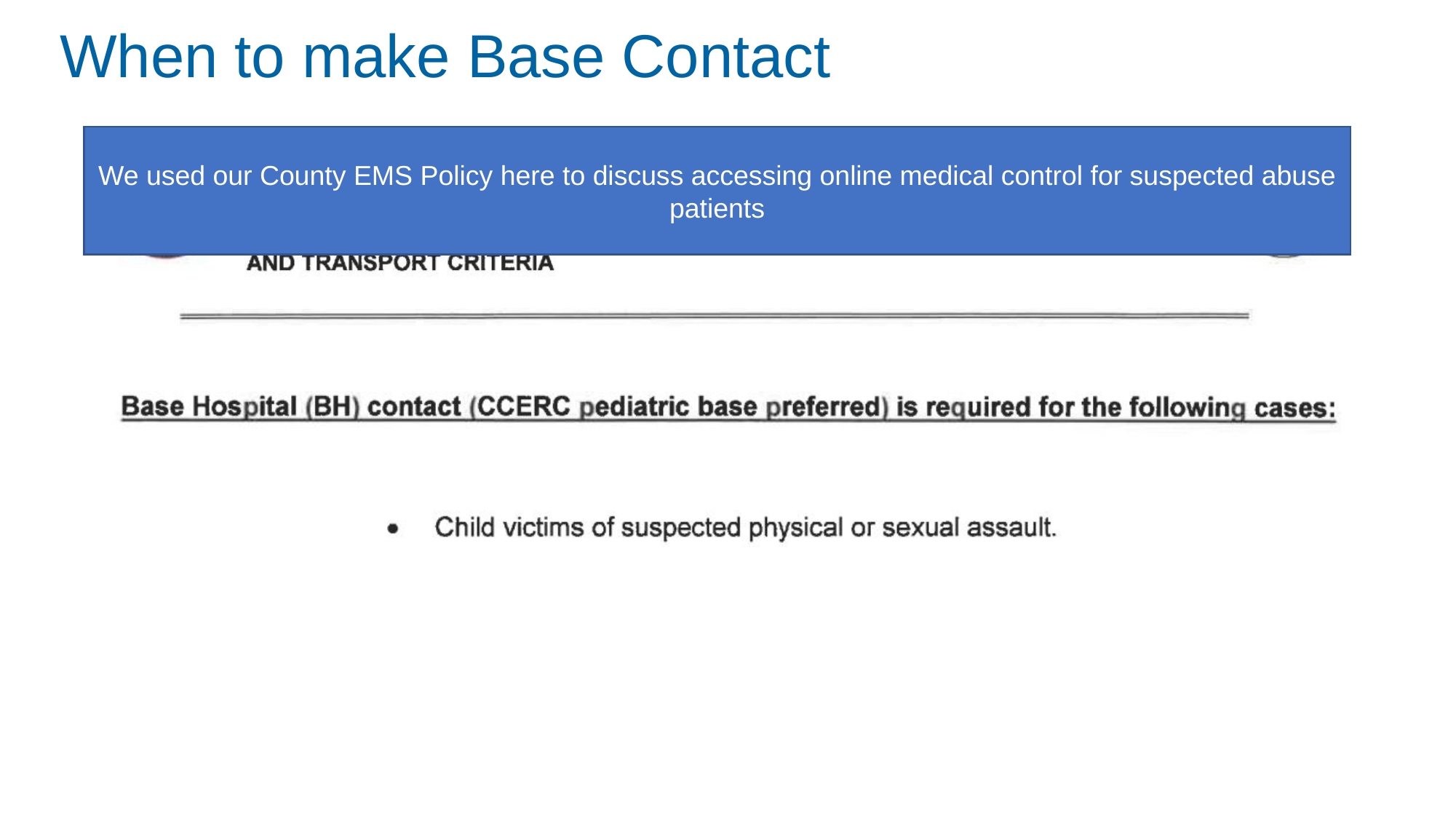

# When to make Base Contact
We used our County EMS Policy here to discuss accessing online medical control for suspected abuse patients
